# Integrative Meta-Analysis of Differential Gene Expression in Acute Myeloid Leukemia

**DOI:** 10.1371/journal.pone.0009466

**Published:** 2010-03-01

**Authors:** Brady G. Miller, John A. Stamatoyannopoulos

**Affiliations:** 1 Department of Hematology, University of Washington, Seattle, Washington, United States of America; 2 Department of Genome Sciences, University of Washington, Seattle, Washington, United States of America; Baylor College of Medicine, United States of America

## Abstract

**Background:**

Acute myeloid leukemia (AML) is a heterogeneous disease with an overall poor prognosis. Gene expression profiling studies of patients with AML has provided key insights into disease pathogenesis while exposing potential diagnostic and prognostic markers and therapeutic targets. A systematic comparison of the large body of gene expression profiling studies in AML has the potential to test the extensibility of conclusions based on single studies and provide further insights into AML.

**Methodology/Principal Findings:**

In this study, we systematically compared 25 published reports of gene expression profiling in AML. There were a total of 4,918 reported genes of which one third were reported in more than one study. We found that only a minority of reported prognostically-associated genes (9.6%) were replicated in at least one other study. In a combined analysis, we comprehensively identified both gene sets and functional gene categories and pathways that exhibited significant differential regulation in distinct prognostic categories, including many previously unreported associations.

**Conclusions/Significance:**

We developed a novel approach for granular, cross-study analysis of gene-by-gene data and their relationships with established prognostic features and patient outcome. We identified many robust novel prognostic molecular features in AML that were undetected in prior studies, and which provide insights into AML pathogenesis with potential diagnostic, prognostic, and therapeutic implications. Our database and integrative analysis are available online (http://gat.stamlab.org).

## Introduction

Acute myeloid leukemia (AML) is a heterogeneous disease with overall poor survival. The prognosis of AML is highly conditioned on the presence of specific cytogenetic and molecular abnormalities. Among AMLs with abnormal cytogenetics, the presence of t(8;21), t(15;17) or inv(16) is widely recognized as conferring favorable prognosis, while a variety of other chromosomal aberrations define a poor prognostic group.[1] However, the majority of AMLs are cytogenetically normal (CN) and collectively define an intermediate prognostic group. Within the CN group, several molecular abnormalities have been associated with prognosis. For example, *FLT3*-ITD carries a unfavorable prognosis, while both *NPM1* and *CEBPA* mutations confer a favorable prognosis.[2]

Systematic application of gene expression profiling to AML samples has revealed that major prognostic subgroups based on cytogenetics and molecular markers are recapitulated in large-scale gene expression patterns.[3] A large body of AML gene expression profiling studies has emerged together with reported correlations with pathogenesis, diagnosis, risk classification, and outcome prediction.[4], [5], [6], [7], [8], [9], [10], [11], [12], [13], [14], [15], [16], [17], [18], [19], [20], [21], [22], [23], [24], [25], [26], [27], [28], [29], [30], [31], [32], [33] However, these studies have not been systematically compared. Such a comparison has the potential to test the extensibility of conclusions based on single studies, and may provide further insights into AML pathogenesis while exposing potential diagnostic and prognostic markers and therapeutic targets.

*A priori*, there are two general approaches to comparing gene expression profiling studies. The first and most rigorous approach requires normalization and re-analysis of raw expression data. However, this approach is not practical in cases where raw data are not available from a significant number of studies or is in an unusable form. Indeed, a recent review revealed that only one third of published papers have deposited raw data that are considered robust enough to allow valid multi-study comparisons.[34] An alternative approach focuses on comparative analysis of the published lists of significantly over-expressed or under-expressed genes.[35] This type of analysis involves discovery of gene intersections in published lists, and has been effectively utilized in a variety of contexts such as identification of biomarkers in thyroid and colorectal cancer.[36], [37] Although several tools and repositories have been developed to facilitate identification of significant gene intersections[38], [39], [40], the heterogeneity of the published gene lists for AML require development of a novel approach that will allow a fine-grained comparison and analysis.

**Figure 1:**
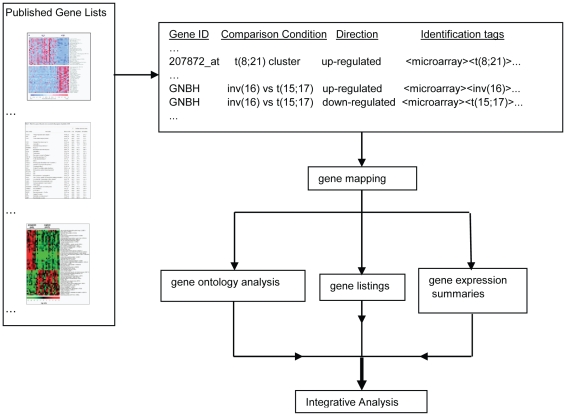
Tag-based classification method flowchart.

In this paper we describe a systematic, fine-grained multi-study comparison of heterogeneous differentially expressed gene sets emerging from 25 expression profiling studies of AML published between 1999 and 2008. Our approach includes collection of the published gene lists, standardized annotation of each listed gene with identification tags, and a functional analysis of the gene lists that are associated with each identification tag (**Figure 1**). We identified high interest genes in AML along with genes and functional gene ontology (GO) categories associated with prognosis and common AML subtypes. We discovered many robust novel prognostic molecular features that were undetected in prior studies. Our results provide novel insights into AML pathogenesis with potential diagnostic, prognostic, and therapeutic implications.

## Results

### Categorization of Differentially Expressed Genes

A total of 15,809 expression features were available from 25 studies, utilizing 10 different microarray platforms, and comprising a total of 2,744 patient samples (**Table 1**). Of the 15,809 expression features, 7,416 were classified as up-regulated, 6,419 were classified as down-regulated, and 1,974 were not classified with respect to an expression direction. A total of 14,385 (91%) expression features could be mapped to a gene symbol in the UCSC hg18 database, which comprised a total of 4,918 genes.

**Table 1:** Acute Myelogenous Leukemia expression profiling studies included in analysis.

Reference	Platform	Disease	No. of samples	No. of differentially expressed features	No. of differentially expressed mapped features
**Golub et al, 1999^4^**	Affymetrix HU6000	AML/ALL	72	100	78
**Okutsu et al, 2002^6^**	Custom cDNA 23,040 clones	AML	76	491	355
**Schoch et al, 2002^7^**	Affymetrix U95Av2	AML	37	150	140
**Debernardi et al, 2003^8^**	Affymetrix U95Av2	AML	28	77	75
**Kohlmann et al, 2003^9^**	Affymetrix U95Av2 Affymetrix U133A	AML/ALL	90	156	147
**Yagi et al, 2003^10^**	Affymetrix U95Av2	AML	54	1,910	1,753
**Bullinger et al, 2004^11^**	Custom cDNA 39,711 clones	AML	116	1,040	855
**Lacayo et al, 2004^12^**	Custom cDNA 42,749 clones	AML	100	436	329
**Neben et al, 2004^13^**	Custom cDNA 4211 clones	AML	29	170	162
**Ross et al, 2004^14^**	Affymetrix U133A	AML	150	713	682
**Valk et al, 2004** * 15	Affymetrix U133A	AML	293	779	745
**Vey et al, 2004^16^**	DiscoveryChip cDNA 9,039 clones	AML	55	197	119
**Alcalay et al, 2005^17^**	Affymetrix U133A	AML	78	554	541
**Gutierrez et al, 2005^18^**	Affymetrix U133A	AML	43	181	167
**Heuser et al, 2005^20^**	Custom cDNA 41,424 clones	AML	137	178	146
**Haferlach et al, 2005^19^**	Affymetrix U133A	AML	35	20	19
**Neben et al, 2005^21^**	Custom cDNA 4,211 clones	AML	110	250	250
**Verhaak et al, 2005^22^**	Affymetrix U133A	AML	275	568	555
**Radmacher et al, 2006^25^**	Affymetrix U133plus2.0	AML	64	314	304
**Wilson et al, 2006^26^**	Affymetrix U95Av2	AML	170	705	674
**Gal et al, 2006^23^**	Affymetrix U133A	AML	5	822	724
**Bullinger et al, 2007^27^**	Custom cDNA 39,711 clones	AML	93	4,120	3,828
**Eisele et al, 2007^28^**	Affymetrix U133A	AML	11	82	82
**Mullighan et al, 2007^33^**	Affymetrix U133A	AML	93	1,188	1,095
**Wouters et al, 2007^31^**	Affymetrix U133plus2.0	AML	530	608	560
**Total**	10 platforms		2,744	15,809	14,385

*Included further analysis of data by Verhaak 2005^22^ and Wilson 2006^26^.

### Standardized Annotation of Gene Expression Features

We annotated each expression feature with standardized identification tags and comparison conditions. The identification tags are a set of descriptors that describe the context of the expression feature, such as the experiment type (RT-PCR or microarray) and the results including prognostic category associations. The database contained 91 unique identification tags (**Table S1**). The comparison conditions describe the samples that are compared in each experiment and the database contained 78 unique comparison conditions (**Table S2**).

### Genes Associated with AML

We then identified genes that were reported in multiple studies. Of the total 4,918 genes, 1,686 (34.3%) were reported in more than one study. We ranked genes that were listed in at least 8 studies by number of references, number of expression platforms, and number of expression features (**Table 2**). Although most of these genes have been associated with AML elsewhere in the literature, several genes (*VCAN* and *PGDS*) were only described in AML cell lines and a surprising number of the genes (*HLA-DPA1*, *ITM2A*, *RBPMS*, *RGS10*, *RNASE2* and *TRH*) were not specifically described in AML. *VCAN* is a component of the extracellular matrix modulating cell adhesion, cell proliferation, cell migration, and extracellular matrix assembly.[41] High expression of *VCAN* has been found in many malignancies, such as melanomas, ovarian, breast, and lung tumors,[41] and in the acute monocytic leukemia cell line, THP-1.[42] *PGDS* is an enzyme that catalyzes the conversion of PGH2 to PGD2, which is a prostaglandin involved in vasodilation, bronchoconstriction, inhibition of platelet aggregation, and recruitment of inflammatory cells.[43] PGDS expression has been reported in two megakaryoblastic cell lines, CMK and Dami.[43] *TRH* is a neurotransmitter/neuromodulator in the central and peripheral nervous system and is released by the hypothalamus to regulate the biosynthesis of TSH in the anterior pituitary gland.[44] *HLA-DPA1* is a HLA class II gene involved in antigen presentation, and has been associated with esophageal squamous dysplasia[45] and pilocytic astrocytomas[46]. *RNASE2* is a cationic ribonuclease toxin found in eosinophil granules[47] and reported to have chemotactic[48] and antiviral[49] activities. *RBPMS* is a RNA-binding protein with an unclear specific function and at least 12 different splice variants.[50] *ITM2A* is a type II transmembrane glycoprotein expressed in vesicles and on the cell surface and has been noted to be up-regulated during T-cell activation.[51] *ITM2A* has been associated with chrondrogenic[52] and myogenic differentiation[53]. *RGS10* acts as a GTPase-activating protein via modulation of Gαi and Gαz signaling[54], and promotes chrondrogenic differentiation in mice.[55] Expression of *RGS10* has been noted in lymphocytes[56] and rat platelets[57].

**Table 2:** Genes most frequently published in AML expression studies.

Rank	Gene symbol	No. of references	No. of platforms	No. of differentially expressed features	Gene name
**1**	**HOXB2**	12	6	32	homeobox B2
**2**	**PBX3**	12	5	31	pre-B-cell leukemia homeobox 3
**3**	**HOXA9**	11	4	35	homeobox A9
**4**	**POU4F1**	11	3	29	POU class 4 homeobox 1
**5**	**TSPAN7**	10	5	16	tetraspanin 7
**6**	**MYH11**	10	3	38	myosin, heavy chain 11, smooth muscle
**7**	**RUNX1T1**	10	3	34	runt-related transcription factor 1; translocated to, 1 (cyclin D-related)
**8**	**TRH**	10	3	21	thyrotropin-releasing hormone
**9**	**HLA-DPA1**	10	3	20	major histocompatibility complex, class II, DP alpha 1
**10**	**HOXB5**	9	5	32	homeobox B5
**11**	**SPARC**	9	5	16	secreted protein, acidic, cysteine-rich (osteonectin)
**12**	**HOXA10**	9	4	34	homeobox A10
**13**	**RNASE2**	9	4	18	ribonuclease, RNase A family, 2 (liver, eosinophil-derived neurotoxin)
**14**	**CD34**	9	3	16	CD34 molecule
**15**	**MEIS1**	9	3	16	Meis homeobox 1
**16**	**RUNX3**	8	5	23	runt-related transcription factor 3
**17**	**VCAN**	8	5	22	versican proteoglycan
**18**	**RBPMS**	8	4	21	RNA binding protein with multiple splicing
**19**	**HOXA4**	8	4	18	homeobox A4
**20**	**MN1**	8	4	16	meningioma (disrupted in balanced translocation) 1
**21**	**PRAME**	8	4	11	preferentially expressed antigen in melanoma
**22**	**JAG1**	8	3	20	jagged 1 (Alagille syndrome)
**23**	**ITM2A**	8	3	18	integral membrane protein 2A
**24**	**RGS10**	8	3	17	regulator of G-protein signaling 10
**25**	**PGDS** *	8	2	12	prostaglandin D2 synthase, hematopoietic

The genes reported in at least eight independent studies are presented here. In order of preference, the genes are ranked by the number of independent studies, the number of unique platforms, and the total number of differentially expressed features.

*Gene symbol is not approved by HUGO Gene Nomenclature Committee.

### Concordant Gene Expression Identified in Multiple Studies

We then identified prognostic categories that were reported in greater than 3 independent studies and stratified these by number of genes, differential expression direction, and number of independent studies (**Table 3**). This analysis revealed the existence of genes in categories of AML that were strictly up-regulated or down-regulated across multiple studies.

**Table 3:** Number of genes and independent publications with selected prognostic categories.

Tag (total genes) (total references)		No. genes in 1 study	No. genes in 2 studies	No. genes in 3 studies	No. genes in 4 studies	No. genes in 5 studies	No. genes in 6 studies	No. genes in 7 studies	No. genes in 8 studies
***poor prog*** (1727) (12)	all genes	1559	138	27	3				
	up-regulated	586	28	2					
	down-regulated	943	55	10	1				
***good prog*** (1638) (11)	all genes	1484	134	18	1	1			
	up-regulated	925	62	9	1				
	down-regulated	528	24	2					
***NPM1 mut*** (1169) (5)	all genes	978	147	32	11	1			
	up-regulated	541	42	18	7	1			
	down-regulated	436	96	13	2				
***t(15;17)*** (230) (9)	all genes	188	25	9	7			1	
	up-regulated	115	17	5	6				
	down-regulated	56	8	4	1			1	
***inv(16)*** (1322) (9)	all genes	1197	88	23	7	6			1
	up-regulated	533	44	12	7	2		1	
	down-regulated	285	15	4	1				
***t(8;21)*** (1195) (9)	all genes	1057	92	23	15	4	1	2	1
	up-regulated	253	32	13	4	1	2	1	
	down-regulated	552	27	5	2				
***11q23*** (482) (5)	all genes	459	19	4					
	up-regulated	65	1						
	down-regulated	45	3						
***FLT3-ITD*** (235) (4)	all genes	224	10	1					
	up-regulated	135	2						
	down-regulated	67							
***normal cyto*** (519) (6)	all genes	480	36	3					
	up-regulated	173	1						
	down-regulated	206	6						

The AML prognosis and subtype identification tags reported in greater than 3 independent studies are shown with the number of genes listed by number of independent studies and differential expression direction. Identification tag descriptions can be found in Table S1. Note that the following tags are abbreviated: poor prog is poor prognosis, good prog is good prognosis, NPM1 mut is NPM1 mutation, and normal cyto is normal cytogenetics.

### Hierarchical Cluster Analyses of Differentially Expressed Genes

**Figure 2:**
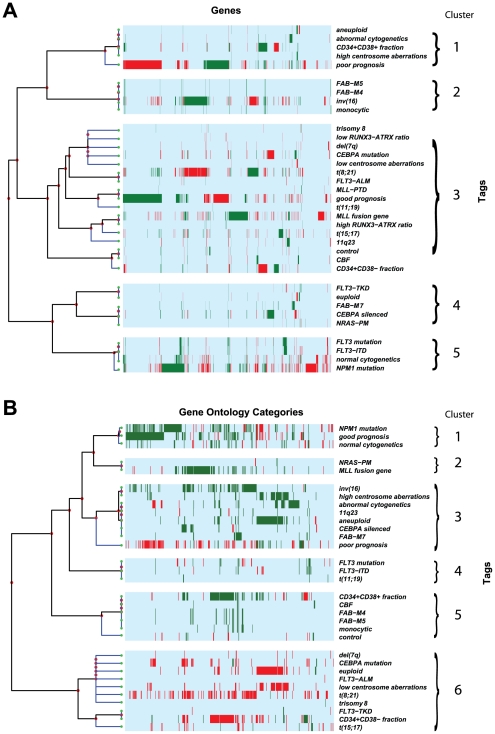
Hierarchical cluster analyses. Strict up-regulation is green and strict down-regulation is red, while light blue represents no reported specific direction. Identification tag descriptions can be found in Table S1. (**A**) Hierarchical cluster analysis of the 3998 differentially expressed genes (x-axis) of AML prognostic categories (y-axis). For illustration purposes, we notated and manually separated 5 major clusters. Cluster 1 includes aneuploid, abnormal cytogenetics, CD34+CD38+ AML fraction, high centrosome aberrations and poor prognosis. Cluster 2 includes FAB-M4, FAB-M5, inv(16) and monocytic. Cluster 3 includes a large group of heterogenous identification tags. Cluster 4 identifies FLT3-TKD, euploid, FAB-M7, *CEBPA* silenced, and NRAS-PM. Cluster 5 includes *FLT3* mutation, FLT3-ITD, normal cytogenetics and *NPM1* mutation. (**B**) Hierarchical cluster analysis of the 541 differential GO categories (x-axis) of AML prognostic categories (y-axis). For illustration purposes, we notated and manually separated 6 major clusters. Cluster 1 includes *NPM1* mutation, good prognosis and normal cytogenetics. Cluster 2 includes NRAS-PM and MLL fusion gene. Cluster 3 includes inv(16), high centrosome aberrations, abnormal cytogenetics, 11q23, aneuploid, CEBPA silenced, FAB-M7, and poor prognosis. Cluster 4 includes *FLT3* mutation, FLT3-ITD and t(11;19). Cluster 5 includes CD34+CD38+ AML fraction, CBF, FAB-M4, FAB-M5, monocytic, and normal patient controls. Cluster 6 includes a large group of heterogenous identification tags.

We next performed hierarchical clustering of differentially expressed genes associated with AML prognostic categories (**Figure 2A**). We identified 5 major clusters. Cluster 1 includes aneuploid, abnormal cytogenetics, CD34+CD38+ AML fraction, high centrosome aberrations and poor prognosis. Cluster 2 includes FAB-M4, FAB-M5, inv(16) and monocytic. Cluster 3 includes a large group of heterogeneous identification tags. Cluster 4 identifies FLT3-TKD, euploid, FAB-M7, *CEBPA* silenced, and NRAS-PM. Cluster 5 includes *FLT3* mutation, FLT3-ITD, normal cytogenetics and *NPM1* mutation. Cluster 1 corresponds to features noted in poor prognosis AML, cluster 2 corresponds to features found in monocytic differentiated AML, while cluster 5 includes AML subtypes that are found in cytogenetically normal (CN) AML.

### Hierarchical Cluster Analyses of Gene Functional Categories

Next, we performed hierarchical cluster analyses of functional categories associated with AML related identification tags (**Figure 2B**). We identified 6 clusters. Cluster 1 includes *NPM1* mutation, good prognosis and normal cytogenetics. Cluster 2 includes NRAS-PM and MLL fusion gene. Cluster 3 includes inv(16), high centrosome aberrations, abnormal cytogenetics, 11q23, aneuploid, CEBPA silenced, FAB-M7, and poor prognosis. Cluster 4 includes *FLT3* mutation, FLT3-ITD and t(11;19). Cluster 5 includes CD34+CD38+ AML fraction, CBF, FAB-M4, FAB-M5, monocytic, and normal patient controls. Cluster 6 includes a large group of heterogeneous identification tags. Cluster 1 corresponds to features noted in good prognosis AML while cluster 3 corresponds to several features noted in poor prognosis AML.

### Analysis of HOX and TALE Gene Families

The *HOX*/*TALE* genes encode transcription factors regulating pattern formation, differentiation, and proliferation, and there is considerable evidence in the literature associating dysregulation of *HOX*/*TALE* genes in AML. [58] We identified 24 homeodomain (*HOX*/*TALE*) genes that were listed in at least one study (**Table S3**). We observed an overall increase in *HOX*/*TALE* expression in AML with normal cytogenetics, *NPM1* mutations, *FLT3* mutations, and 11q23 abnormalities involving the *MLL* gene. Overall decreases in *HOX*/*TALE* expression were observed in normal CD34+ cells, AML with *CEBPA* mutations and AML with abnormal cytogenetics, specifically t(15;17), t(8;21), and inv(16). This pattern is consistent with previous RT-PCR studies screening HOX/TALE genes expression levels[59], [60], [61], [62], [63], [64], although the association of *CEBPA* mutations with decreased *HOX*/*TALE* expression has not been reported previously.

### Analysis and Replication of Prognostic Categories

Next, we focused on genes associated with good and poor prognosis. We defined ‘good prognosis’ as a relatively increased overall survival or disease free survival or response to therapy. We defined ‘poor prognosis’ as a relatively decreased overall survival or disease free survival or response to therapy. The good prognosis and poor prognosis gene sets are largely reciprocal. Surprisingly, only 9.6% of these genes were replicated with concordant expression directions in more than one study. The top ranked up-regulated and down-regulated genes associated with poor prognosis are shown in **Table 4** and **Table 5** respectively. The top ranked up-regulated and down-regulated genes associated with good prognosis are shown in **Table S4**.

**Table 4:** Top ranked up-regulated genes associated with poor prognosis.

Rank	Gene symbol	No. of specific references	Total no. of references	Total no. of platforms	Total no. of differentially expressed features	Gene name
**1**	**BCL11A**	3	5	4	19	B-cell CLL/lymphoma 11A (zinc finger protein)
**2**	**TBXAS1**	3	5	4	11	thromboxane A synthase 1 (platelet, cytochrome P450, family 5, subfamily A)
**3**	**HOXB5**	2	9	5	32	homeobox B5
**4**	**HOXA10**	2	9	4	34	homeobox A10
**5**	**CD34**	2	9	3	16	CD34 molecule
**6**	**RBPMS**	2	8	4	21	RNA binding protein with multiple splicing
**7**	**HOXA4**	2	8	4	18	homeobox A4
**8**	**MN1**	2	8	4	16	meningioma (disrupted in balanced translocation) 1
**9**	**GNAI1**	2	6	3	12	guanine nucleotide binding protein (G protein), alpha inhibiting, activity polypeptide 1
**10**	**SKAP2**	2	5	4	21	src kinase associated phosphoprotein 2
**11**	**MCM3**	2	5	4	9	minichromosome maintenance complex component 3
**12**	**CLIP2**	2	5	3	8	CAP-GLY domain containing linker protein 2
**13**	**DAPK1**	2	5	3	8	death-associated protein kinase 1
**14**	**GUCY1A3**	2	4	4	8	guanylate cyclase 1, soluble, alpha 3
**15**	**ANGPT1**	2	4	3	11	angiopoietin 1
**16**	**MTHFD1**	2	4	3	6	methylenetetrahydrofolate dehydrogenase (NADP+ dependent) 1, methenyltetrahydrofolate cyclohydrolase, formyltetrahydrofolate synthetase
**17**	**MAP7**	2	3	3	14	microtubule-associated protein 7
**18**	**UGCGL2**	2	3	3	11	UDP-glucose ceramide glucosyltransferase-like 2
**19**	**SH2B3**	2	3	3	6	SH2B adaptor protein 3
**20**	**FLT3**	2	3	3	5	fms-related tyrosine kinase 3

In order of preference, the genes are ranked by the number of poor prognosis related independent studies, the total number of independent studies, the total number of unique platforms, and the total number of features. Genes that were also associated with good prognosis with the same expression direction are not shown.

**Table 5:** Top ranked down-regulated genes associated with poor prognosis.

Rank	Gene symbol	No. of specific references	Total no. of references	Total no. of platforms	Total no. of differentially expressed features	Gene name
**1**	**EML4**	4	4	3	22	echinoderm microtubule associated protein like 4
**2**	**C3AR1**	3	6	2	9	complement component 3a receptor 1
**3**	**SMG1** *	3	5	4	26	phosphatidylinositol 3-kinase-related protein kinase
**4**	**FOXO1**	3	5	4	15	forkhead box O1
**5**	**IL6ST**	3	4	3	18	interleukin 6 signal transducer (gp130, oncostatin M receptor)
**6**	**UGCG**	3	4	3	12	UDP-glucose ceramide glucosyltransferase
**7**	**ADFP**	3	4	2	12	adipose differentiation-related protein
**8**	**AZU1**	3	4	2	8	azurocidin 1 (cationic antimicrobial protein 37)
**9**	**SNX9**	3	3	2	14	sorting nexin 9
**10**	**PIK3R4**	3	3	2	8	phosphoinositide-3-kinase, regulatory subunit 4, p150
**11**	**SEMA3F**	3	3	2	6	sema domain, immunoglobulin domain (Ig), short basic domain, secreted, (semaphorin) 3F
**12**	**JAG1**	2	8	3	20	jagged 1 (Alagille syndrome)
**13**	**CD3D**	2	7	4	21	CD3d molecule, delta (CD3-TCR complex)
**14**	**SLC7A7**	2	6	5	14	solute carrier family 7 (cationic amino acid transporter, y+ system), member 7
**15**	**ENDOD1**	2	6	4	13	endonuclease domain containing 1
**16**	**GYPC**	2	6	4	11	glycophorin C (Gerbich blood group)
**17**	**ISG20**	2	6	3	17	interferon stimulated exonuclease gene 20 kDa
**18**	**EZR**	2	5	4	13	Ezrin
**19**	**AGRN**	2	5	4	12	Agrin
**20**	**NRP1**	2	5	3	9	neuropilin 1

In order of preference, the genes are ranked by the number of poor prognosis related independent studies, the total number of independent studies, the total number of unique platforms, and the total number of features. Genes that were also associated with good prognosis with the same expression direction are not shown.

*Gene symbol is not approved by HUGO Gene Nomenclature Committee.

### Genes Associated with Prognosis

The majority of the top-ranked genes up-regulated in poor and good prognosis, which are listed in **Table 4**, **Table 5**, and **Table S4**, have not been described elsewhere in human AML literature. Although not associated elsewhere with prognosis, *HOXB5* [65], *DAPK1* [66], *ANGPT1* [67], *TCF4* [68], *C3AR1* [69], *CAT* [70], *IL6ST* [71], *JAG1* [32], *EZR* [32], *TP53BP2* [72] and *TNFAIP2* [73] have been described in AML. *HOXA10*, *CD34*, *HOXA4*, *MN1*, *NME1*, *FOXO1*, *NRP1, UGCG* and *FLT3* are the only genes listed that have been associated with prognosis of AML in other studies. These studies have described up-regulation of *MN1* [74], *NME1* [75], *HOXA10* [59], and FLT3[76] in poor prognosis AML which correlates with our comparison, while there are conflicting reports of *HOXA4* [59], [60] and *CD34* gene expression in poor prognosis AML. *CD34* is notable and likely represents a false positive result in our comparison. Although up-regulation of *CD34* was initially described to correlate with a decreased response to therapy,[77] it is has since been shown that up-regulation of this gene actually correlates with abnormal cytogenetics, including t(8;21), and is not associated with a decrease in overall survival or disease-free survival.[78] Phosphorylation of *FOXO1* has been reported to correlate with decreased overall survival in AML, although transcript expression levels have not been reported as having any correlation with overall survival.[79] Up-regulation of both *NRP1* [80] and *UGCG* [81] have been previously correlated with decreased survival and chemoresistance in AML respectively, which both contradict the results of our comparison.

### Functional Categories and Prognosis

**Figure 3:**
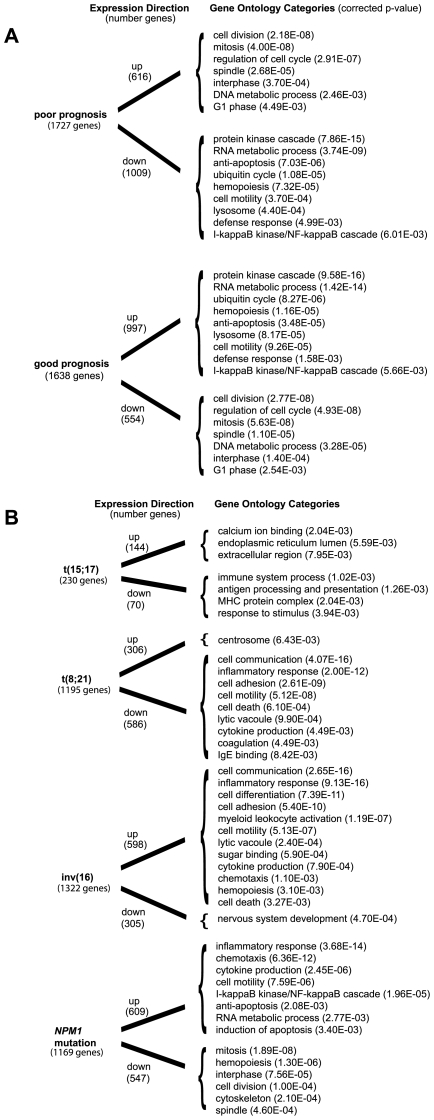
Functional category comparisons. (**A**) Significantly over-represented functional gene ontology (GO) categories of interest in up-regulated and down-regulated genes found in poor prognosis and good prognosis are compared; the comprehensive functional gene ontology listings can be found in Table S5, Table S6, Table S7, and Table S8. (**B**) Significantly over-represented functional gene ontology (GO) categories of interest in up-regulated and down-regulated genes found in AML with *NPM1* mutation, t(15;17), t(8;21) and inv(16) are compared; the comprehensive functional gene ontology listings can be found in Table S13, Table S14, Table S15, Table S16, Table S17, Table S18, Table S19, Table S20. Corrected p-value is the Bonferroni multiple hypothesis.

We then identified the functional categories associated with poor prognosis and good prognosis. The specific over-represented functional categories of the up-regulated genes and down-regulated genes associated with poor prognosis and good prognosis are summarized in **Figure 3A**. Detailed tables describing the over-represented functional categories of up-regulated genes and down-regulated genes associated with poor prognosis and good prognosis are listed in **Table S5**, **Table S6**, **Table S7** and **Table S8** respectively. Interestingly, many of the over-represented functional categories of up-regulated genes associated with poor prognosis were shared with up-regulated genes in aneuploidy, high centrosome aberrations and CD34+CD38+ AML fraction, and down-regulated genes in euploidy, low centrosome aberrations, *NPM1* mutations, good prognosis AML, CD34+CD38- AML fraction, and FLT3-ITD. These results are consistent with increased expression of genes involved in differentiation and apoptosis dysregulation in good prognosis AML and increased expression of genes involved in proliferation in poor prognosis AML.

### Analysis of Molecular and Cytogenetic Subtypes

We then surveyed specific molecular and cytogenetic subtypes of AML that reported genes in greater than 3 independent studies. This includes NPM1 mutations, t(15;17), inv(16), and t(8;21), which are all known to portend a good prognosis. [1], [82] The top-ranked up-regulated and down-regulated genes associated with *NPM1* mutations, t(15;17), inv(16), and t(8;21) are shown in **Table S9**, **Table S10**, **Table S11** and **Table S12** respectively. The specific over-represented functional categories of the up-regulated genes and down-regulated genes associated with *NPM1* mutations, t(15;17), inv(16), and t(8;21) are summarized in **Figure 3B**. Notably, NPM1 mutation's functional categories were concordant with good prognosis AML. AML with t(15;17) illustrated down-regulation of genes involved in the immune system. Interestingly, t(8;21) and inv(16) mirrored each other in terms of direction of their common functional categories because of the significant proportion of studies that directly compared these two entities. Detailed tables describing the over-represented functional categories of up-regulated genes and down-regulated genes associated with *NPM1* mutations, t(15;17), inv(16), and t(8;21) are listed in **Table S13**, **Table S14**, **Table S15**, **Table S16**, **Table S17**, **Table S18**, **Table S19** and **Table S20** respectively.

## Discussion

We developed a methodology for the comparison of published heterogeneous gene lists, and we developed a web application (http://gat.stamlab.org) to facilitate access to the study data. This approach permitted a granular multi-study comparison of gene lists and functional gene ontology classifications. To our knowledge, the body of published AML gene expression profiling studies in the form of published gene lists has not been systematically compared.

We extracted a list of 4918 genes that were reported in 25 gene expression profiling studies of AML. We found that a considerable amount of the genes (32.7%) were published in more than one study, and we described a list of 25 genes that were reported in greater than 8 studies. Although most of these genes have been associated with AML elsewhere in the literature, several genes (*VCAN* and *PGDS*) have only been described in AML cell lines and a surprising number of the genes (*HLA-DPA1*, *ITM2A*, *RBPMS*, *RGS10*, *RNASE2* and *TRH*) have not been specifically described in AML.

We identified gene sets that were associated with good prognosis and poor prognosis (overall survival, disease free survival, or response to therapy) in AML across multiple studies. Surprisingly, only 9.6% of these genes were replicated with concordant expression directions in more than one study. We surveyed the higher ranked genes that were reported in multiple studies, and noted the majority of these genes were not described elsewhere in human AML.

We also identified functional gene ontology categories that are associated with prognosis in AML, which are consistent with increased expression of genes involved in differentiation and apoptosis dysregulation in good prognosis AML and increased expression of genes involved in proliferation in poor prognosis AML. A study included in our comparison that examined survival in CBF AML also associated up-regulation of proliferation GO categories with decreased survival and associated up-regulation of RNA metabolism and apoptosis dysregulation GO categories with increased survival.[27]

We identified differentially expressed genes across multiple studies that were associated with specific subtypes of AML including t(15;17), inv(16), t(8;21), and *NPM1* mutations. For example, there were 5 papers in our comparison that reported gene lists associated with *NPM1* mutations, and all 5 of these papers reported up-regulation of *SMC4*. Additionally, we also identified functional gene ontology categories that were associated with each of these AML subtypes. Interestingly, the functional gene ontology sets of AML with the *NPM1* mutation were similar to good prognosis AML, which is expected considering *NPM1* mutations impart a favorable prognosis.

Our comparison included 24 homeodomain (*HOX*/*TALE*) genes with 7 listed in more than 7 papers. The *HOX*/*TALE* genes encode transcription factors regulating pattern formation, differentiation, and proliferation. Orderly *HOX* gene activation is essential for normal hematopoiesis with *HOX* genes preferentially expressed in the hematopoietic stem cell compartment and then down-regulated following differentiation and maturation.[58] There is considerable evidence in the literature associating dysregulation of *HOX*/*TALE* genes in AML.[58] Constitutive expression of *HOXA7*, *HOXA9*, *HOXA10*, *HOXB3*, and *HOXB8* in mice results in acute leukemia,[83], [84], [85], [86] and recurrent chromosomal translocations in humans involving *HOXA9* [87], *PBX1* [88], and *HOX11* [89] results in leukemia. The *MLL* gene is a known positive regulator of *HOX*/*TALE* expression and translocations involving the *MLL* gene have been associated with increased expression of HOXA4-11, *MEIS1*, and *PBX1*.[58]

Our comparison showed a general increase in *HOX*/*TALE* expression in AML with normal cytogenetics, *NPM1* mutations, *FLT3* mutations, and 11q23 abnormalities involving the *MLL* gene while showing an overall decrease in *HOX*/*TALE* expression in normal patient CD34+ cells, AML with *CEBPA* mutations and AML with abnormal cytogenetics, specifically t(15;17), t(8;21), and inv(16). All of the above trends, except for *CEBPA* mutations, have been reported and confirmed in several RT-PCR studies.[59], [60], [61], [62], [63], [64] To our knowledge, the association of *CEBPA* mutations with decreased *HOX*/*TALE* expression has not been reported previously. Several of the *HOX*/*TALE* genes, specifically *HOXB2*, *PBX3* and *MEIS1*, were also shown in our comparison to have increased expression in inv(16) when compared to t(8;21), which is supported by two recent RT-PCR studies[59], [60]. Exceptions to the above trends in our comparison include decreased expression of *HOXB2* with *MLL* translocations, decreased expression of *PBX2* with *MLL* translocations and *NPM1* mutations, and decreased expression of *HOXC4* with *NPM1* mutations.

Several RT-PCR studies have associated increased expression of *HOXA*1-10 and *MEIS1* with decreased overall survival in AML,[59], [61] although recently a RT-PCR study did associate decreased expression of *HOXA4* with decreased overall survival in CN AML[60]. Several RT-PCR studies have also associated high risk cytogenetics with increased expression of *HOX*/*TALE* genes[58], [61] and an RT-PCR study has associated increased expression of *FLT3* or *FLT3* mutations in CN AML with increased expression of *HOX*/*TALE* genes[63]. In poor prognosis (includes decreased overall survival, disease free survival, or response to therapy) AML, our comparison showed increased expression of several *HOX*/*TALE* genes, specifically *HOXA4*, *HOXA10*, *HOXB5* and *PBX1*, while showing decreased expression of *MEIS1* and contradictory expression directions of *HOXB2* and *PBX3*. Although an overall increase of *HOX*/*TALE* expression in poor prognosis AML has been reported, there are several contradictions to this including *MEIS1*, *HOXB2* and *PBX3* in our comparison and *HOXA4* in an outside RT-PCR study[60]. Additionally, the overall trend of increased *HOX*/*TALE* expression in poor prognosis AML does not appear specific because our comparison and the literature also report increased expression of *HOX*/*TALE* genes in CN AML and AML with *NPM1* mutations. This point is well illustrated by an RT-PCR study using a classifier with 17 homeodomain genes that was able to differentiate favorable cytogenetics from intermediate/unfavorable cytogenetics, however unable to differentiate intermediate from unfavorable cytogenetics.[59]

There were several intriguing potential targets of therapy uncovered during our analysis. *TBXAS1* is an enzyme that converts prostaglandin H2 into thromboxane A2.[90] Thromboxane A2 induces platelet aggregation, smooth muscle contraction, and possibly modulates mitogenesis and apoptosis.[91] Although there have been no previous reports describing *TBXAS1* expression in AML, our comparison included three papers that associated increased expression of *TBXAS1* with a poor prognosis. In bladder cancer cells, pharmacologic inhibition of *TBXAS1* with furegrelate or ozagrel induced apoptosis and enhanced sensitivity to chemotherapy,[92] which does suggest that pharmacologic inhibition of this enzyme has potential for treatment in AML. *SEMA3F* is a secreted protein that has been reported to function as a axon guidance factor, a tumor suppressor gene in small cell lung cancer, a inhibitor of angiogenesis, and a possible direct inhibitor of tumor cell migration and attachment.[93] Although there have been no previous reports describing *SEMA3F* expression in AML, our comparison included three papers that associated increased expression of *SEMA3F* with a good prognosis, which suggest that a *SEMA3F* analog could have potential for treatment in AML.

Our methodology was shown to be especially useful in systematically identifying commonly reported genes and pathways in the heterogeneous disease of AML. Our method is flexible and ensures the inclusion of all pertinent studies into the analysis and is accompanied by an online analysis and database querying tool for other investigators. To ensure the inclusion of all possible pertinent studies, our methodology does not require raw data and can incorporate both published differential gene lists that are not quantified and published gene lists with no reported direction of expression (12% of the published expression features were not associated with a direction). Another strategy that utilizes gene list comparisons across studies has been published by Griffith et al. and Chan et al.[36], [37] Their method successfully identified biomarkers in thyroid and colorectal cancer, however, we chose not to employ their method because each feature requires an explicit expression direction and a quantified expression value.

A potential disadvantage of our methodology is the wide variety of methods employed by the individual studies, which include sample populations, sample sizes, microarray platform types, statistical analysis methods, and the ultimate decisions of which gene lists the authors decide to publish. This heterogeneity in methods can also be viewed as an advantage. For example, a gene that is listed in two studies that employ different microarray platforms and statistical methods could be considered more meaningful than a gene that is listed in two studies that employ the same microarray platform and statistical methodology. Another potential disadvantage with our methodology is publication bias, because our results are dependent on gene lists the authors have decided to publish within their respective studies. To avoid the introduction of any further bias into our results, we do not attempt to weigh the importance of each study by quality metrics, such as sample size or data quality, thus the resulting gene rankings are simply primarily based on the number of applicable studies the gene was reported in.

In the future, our methodology could be applied to perform comparisons of other malignancies and disease states. The main limitations include the tedious process required to collect the gene lists and the potential for publication bias. However, despite these limitations, our methodology is especially powerful in systematically identifying commonly reported genes and pathways in heterogeneous diseases, such as AML, and is especially useful in cases where the raw gene expression datasets are not available.

## Materials and Methods

### Data Collection and Curation

We queried Pubmed for acute myeloid leukemia expression profiling studies published between 1999 and early 2008. We excluded studies that predominantly examined non-leukemia cells and studies that contained less than 5 patient samples. In total, published gene lists were collected from 25 independent studies (**Table 1**). The published gene lists were processed to obtain the following information: gene symbol; unique identifiers (Accession ID, Affymetrix probe ID, LocusLink ID, UniGene ID); comparison conditions; differential expression; microarray platform; number of samples; PubMed ID; and identification tags. The identification tags are a set of descriptors that describe each expression feature. If two conditions were being compared, then two separate expression features were created with opposite differential expression and opposing identification tags. The notation of the comparison conditions and the identification tags in the database were standardized to allow the gene expression summary analysis and gene ontology analysis, which are both described below. The above processing was accomplished with a combination of parsing with custom Perl scripts, manual transcription, and copying/pasting. This information was then enumerated and formatted with custom Perl scripts to create a flat file database.

### Gene Mapping

The expression features in the collected published lists were referenced by one or more of the following: gene symbol, accession ID, Affymetrix probe ID, LocusLink ID, and/or UniGene ID. These references were mapped to the Gene Symbol in the UCSC human genome hg18 database [94] with custom Perl scripts. If we were unable to map the reference to a Gene Symbol in the UCSC database, then the expression feature was not included in further analysis.

### Tag-Based Classification of Expression with Prognostic Features

We used an integrative approach to assign identification “tags” to gene expression and prognostic categories. A flow chart of the approach is illustrated in **Figure 1**. We assigned identification tags to each datapoint and used a strict nomenclature for comparison conditions.

### Gene Expression Summary

We developed a customized Perl script that incorporates the comparison conditions and identification tags in an algorithm to summarize the expression directions of each mapped gene. These expression summaries can be viewed in an online Browser (http://gat.stamlab.org).(B.G.M and J.A.S., manuscript in preparation)

### Functional Classification of Gene Lists

For functional classification of the gene lists, we used GO::TermFinder[95] for gene ontology (GO)[96]analysis. We downloaded the GO v1.0 OBO database 2/22/2008 release from http://www.geneontology.org. We downloaded the human annotation file version 60.0 and human cross-reference file version 3.39 from the GOA website http://www.ebi.ac.uk/GOA/. We developed custom Perl scripts to create a list of genes that was associated with each identification tag and differential expression direction. These lists of genes were then mapped to the appropriate Swiss-Prot ID with the above mentioned GOA human cross-reference file. To avoid an over-representation bias, we only allowed one Swiss-Prot ID per gene. Statistically significant over-represented GO categories of the Swiss-Prot ID lists were identified with GO:TermFinder; we used the entire GO annotation as the background, and statistical significance was calculating by the Bonferroni multiple hypothesis with a p-value cutoff of 0.01.

### Clustering Analysis

Hierarchical clustering was used to compare the differential expression of elements (genes or gene ontology categories) associated with each identification tag. For each identification tag, strictly up-regulated elements were assigned the value 1, while strictly down-regulated elements were assigned the value 0. Hierarchical clustering was then calculated in the R software package, which employed the method of complete linkage and Canberra distance.

## Supporting Information

Table S1 List of identification tags and descriptions
(0.01 MB PDF)

Table S2 List of comparison conditions
(0.01 MB PDF)

Table S3 Expression summaries of HOX and TALE genes
(0.03 MB PDF)

Table S4 Top ranked genes associated with good prognosis
(0.03 MB PDF)

Table S5 Functional categories of up-regulated genes associated with poor prognosis
(0.02 MB PDF)

Table S6 Functional categories of down-regulated genes associated with poor prognosis
(0.03 MB PDF)

Table S7 Functional categories of up-regulated genes associated with good prognosis
(0.03 MB PDF)

Table S8 Functional categories of down-regulated genes associated with good prognosis
(0.02 MB PDF)

Table S9 Top ranked genes associated with NPM1 mutations
(0.03 MB PDF)

Table S10 Top ranked genes associated with t(15;17)
(0.01 MB PDF)

Table S11 Top ranked genes associated with inv(16)
(0.03 MB PDF)

Table S12 Top ranked genes associated with t(8;21)
(0.03 MB PDF)

Table S13 Functional categories of up-regulated genes associated with NPM1 mutations
(0.03 MB PDF)

Table S14 Functional categories of down-regulated genes associated with NPM1 mutations
(0.02 MB PDF)

Table S15 Functional categories of up-regulated genes associated with t(15;17)
(0.02 MB PDF)

Table S16 Functional categories of down-regulated genes associated with t(15;17)
(0.02 MB PDF)

Table S17 Functional categories of up-regulated genes associated with inv(16)
(0.03 MB PDF)

Table S18 Functional categories of down-regulated genes associated with inv(16)
(0.02 MB PDF)

Table S19 Functional categories of up-regulated genes associated with t(8;21)
(0.01 MB PDF)

Table S20 Functional categories of down-regulated genes associated with t(8;21)
(0.03 MB PDF)
