# Supplementary material for: Integrative Meta-Analysis of Differential Gene Expression in Acute Myeloid Leukemia
Source: PLoS One. 2010 Mar 1;5(3):e9466. doi: 10.1371/journal.pone.0009466 (PMC2830886; doi:10.1371/journal.pone.0009466)
Supplement: Table S1 — List of identification tags and descriptions (0.01 MB PDF) [file pone.0009466.s001.pdf]

Table S1. List of identification tags and descriptions

Identification Tag name	No. of references	No. of differentially expressed features	Identification Tag description
11q23	5	559	11q23 abnormalities
abnormal cytogenetics	1	150	abnormal cytogenetics
adult	12	7254	greater than or equal to 18 years of age
ALL	3	212	acute lymphocytic leukemia
AML	25	15697	acute myeloid leukemia
aneuploid	1	64	aneuploid chromosome number
CBF	2	2410	core binding factor translocation
CD34+CD38- fraction	1	411	CD34+CD38- cell fraction from AML
CD34+CD38+ fraction	1	411	CD34+CD38+ cell fraction from AML
CEBPA mutation	2	402	*CEBPA* gene mutation
CEBPA silenced	1	304	*CEBPA* gene silenced
control	2	55	CD34+ cells from normal patient
del(7q)	1	48	deletion of long arm of chromosome 7
euploid	1	64	euploid chromosome number
EVI1 overexpression	1	28	*EVI1* gene overexpression
FAB	1	213	Associated with FAB, unspecified subtype
FAB-M3	1	20	FAB subtype M3
FAB-M3v	1	20	FAB subtype M3v
FAB-M4	1	40	FAB subtype M4
FAB-M5	1	40	FAB subtype M5
FAB-M7	1	100	FAB subtype M7
FLT3 mutation	4	663	*FLT3* gene mutation
FLT3-ALM	1	23	*FLT3* gene activating loop mutations
FLT3-ITD	4	355	*FLT3* gene internal tandem duplications in juxtamembrane region
FLT3-TKD	1	84	*FLT3* gene point mutation in second tyrosine kinase domain
good prognosis	11	2177	increased overall survival or disease free survival or response to therapy
high centrosome aberrations	1	21	high number of abnormal centrosomes
high RUNX3-ATRX ratio	1	13	high *RUNX3* gene to *ATRX* gene ratio
human	25	15809	human sample
inv(16)	9	1748	inv(16)
low centrosome aberrations	1	21	low number of abnormal centrosomes
low RUNX3-ATRX ratio	1	13	low *RUNX3* gene to *ATRX* gene ratio
microarray	25	15686	microarray experiment
MLL fusion gene	2	944	*MLL* gene chimeric fusions
MLL-PTD	1	19	*MLL* gene partial tandem duplications
monocytic	1	50	monocytic differentiation
normal cytogenetics	6	925	cytogenetically normal (CN) AML
NPM1 mutation	5	1802	*NPM1* gene mutation
NRAS-PM	1	67	*NRAS* gene mutation
patient	25	15809	patient sample
pediatric	5	4297	less than 18 years of age
poor prognosis	12	2336	decreased overall survival or disease free survival or reponse to therapy
prognosis	2	138	associated with overall survival or disease free survival or response to therapy
RT-PCR	7	123	RT-PCR experiment
t(11;19)	1	10	t(11;19)
t(15;17)	9	401	t(15;17)
t(8;14)	1	15	t(8;14)
t(8;21)	9	1671	t(8;21)
t(9;11)	1	144	t(9;11)
t(9;22)	1	16	t(9;22)
T-ALL	1	15	T-cell acute lymphocytic leukemia
trisomy 8	1	14	trisomy 8

Definitions of identification tags with associated studies and number of features.
