# Supplementary material for: Integrative Meta-Analysis of Differential Gene Expression in Acute Myeloid Leukemia
Source: PLoS One. 2010 Mar 1;5(3):e9466. doi: 10.1371/journal.pone.0009466 (PMC2830886; doi:10.1371/journal.pone.0009466)
Supplement: Table S2 — List of comparison conditions (0.01 MB PDF) [file pone.0009466.s002.pdf]

Table S2. List of comparison conditions

Comparison condition	No. of references	No. of differentially expressed features
**Baseline - none**		
"11q23" vs "t(8;14)"	1	6
"11q23" vs "t(9;22)"	1	8
"11q23" vs "T-ALL"	1	6
"AML" vs "ALL"	1	100
"CBF" with "good prognosis" vs "CBF" with "poor prognosis"	1	2310
"CD34+CD38- fraction" vs "CD34+CD38+ fraction"	1	822
"CEBPA silenced" vs "CEBPA mutation"	1	608
"euploid" vs "aneuploid"	1	128
"FLT3 mutation" with "good prognosis" and "low RUNX3-ATRX ratio" vs "FLT3 mutation" with "poor prognosis" and "high RUNX-ATRX ratio"	1	26
"FLT3 mutation" with "good prognosis" vs "FLT3 mutation" with "poor prognosis"	1	260
"FLT3-ITD" vs "FLT3-TKD"	1	64
"FLT3-ITD" vs "NRAS-PM"	1	52
"FLT3-TKD" vs "NRAS-PM"	1	36
"FLT3-ITD" vs "FLT3-ALM"	1	46
"good prognosis" vs "poor prognosis"	6	884
"inv(16)" vs "11q23"	1	8
"inv(16)" vs "t(8;21)"	2	52
"inv(16)" vs "t(8;21)"	1	1810
"low centrosome aberrations" vs "high centrosome aberrations"	1	42
"normal cytogenetics" with "good prognosis" vs "normal cytogenetics" with "poor prognosis"	2	394
"t(15;17)" with "FAB-M3v" vs "t(15;17)" with "FAB-M3"	1	20
"t(15;17)" vs "11q23"	1	8
"t(15;17)" vs "inv(16)"	3	104
"t(15;17)" vs "t(8;21)"	2	60
"t(8;14)" vs "t(9;22)"	1	8
"t(8;14)" vs "T-ALL"	1	8
"t(8;21)" vs "11q23"	1	8
"t(9;22)" vs "T-ALL"	1	8
"trisomy 8" vs "normal cytogenetics"	1	28
**Baseline - AML**		
"11q23" cluster	5	531
"abnormal cytogenetics" cluster	1	150
"AML" cluster	1	435
"CBF" cluster	1	100
"CEBPA mutation" cluster	1	98
"control" cluster	1	15
"del(7q)" cluster	1	48
"EVI1 overexpression" cluster	1	28
"FAB" cluster	1	213
"FAB-M4" and "FAB-M5" cluster	1	40
"FAB-M7" cluster	1	100
"FLT3 mutation" cluster	2	98
"FLT3-ITD" cluster	3	193
"good prognosis" and "normal cytogenetics" and "NPM1 mutation" cluster	1	156
"inv(16)" subgroup cluster	1	42
"inv(16)" cluster	7	719
"MLL fusion gene" cluster	2	844
"MLL-PTD" cluster	1	19
"monocytic" cluster	1	50
"normal cytogenetics" and "NPM1 mutation" cluster	1	40
"normal cytogenetics" with "good prognosis" cluster	1	85
"normal cytogenetics" with "poor prognosis" cluster	1	61
"normal cytogenetics" with "prognosis" cluster	1	86
"normal cytogenetics" cluster	3	89
"NPM1 mutation" and "FLT3-ITD" cluster	1	40
"NPM1 mutation" cluster	3	1566
"poor prognosis" and "control" cluster	1	40
"poor prognosis" cluster	1	299
"prognosis" cluster	1	50

"t(11;19)" cluster	1	10
"t(15;17)" cluster	7	295
"t(8;21)" subgroup cluster	1	28
"t(8;21)" cluster	7	678
"t(9;11)" cluster	1	144
"cluster 1" cluster	1	39
"cluster 16" cluster	1	40
"cluster 3" cluster	1	40
"cluster 4" cluster	1	40
"cluster 7" cluster	1	40
"cluster 8" cluster	1	40
"cluster E" cluster	1	50
**Baseline - ALL**		
"11q23" cluster	1	6
"t(8;14)" cluster	1	4
"T-ALL" cluster	1	4
"t(9;22)" cluster	1	4
**Baseline - AML and ALL**		
"MLL fusion gene" cluster	1	100
**Baseline - Normal Cytogenetics AML**		
"FLT3-ITD" cluster	1	41
"FLT3-TKD" cluster	1	34
"NRAS-PM" cluster	1	23

Standardized annotation of experiment conditions organized by the baseline of the experiment. The number of studies and features associated with each comparison condition are listed. Identification tag descriptions can be found in Table S1.
