# Supplementary material for: Integrative Meta-Analysis of Differential Gene Expression in Acute Myeloid Leukemia
Source: PLoS One. 2010 Mar 1;5(3):e9466. doi: 10.1371/journal.pone.0009466 (PMC2830886; doi:10.1371/journal.pone.0009466)
Supplement: Table S3 — Expression summaries of HOX and TALE genes (0.03 MB PDF) [file pone.0009466.s003.pdf]

Table S3. Expression summaries of HOX and TALE genes

Gene symbol	No. of references	No. of platforms	No. of differentially expressed features	Expression Summary: AML Baseline: up-regulated tags	Expression Summary: AML Baseline: down-regulated tags	Expression Summary: No Baseline (bold tag is up-regulated)
HOXB2	12	6	32	*FLT3-ITD*<br>*NPM1 mutation*<br>*normal cytogenetics*	*CEBPA mutation*<br>*MLL fusion gene*<br>*abnormal cytogenetics*	***inv(16)*** *vs t(15;17)*<br>***inv(16)*** *vs t(8;21)*<br>prognosis*
PBX3	12	5	31	*MLL fusion gene*<br>*NPM1 mutation*<br>*normal cytogenetics*	*abnormal cytogenetics*<br>*inv(16)*<br>*t(15;17)*<br>*control*	***inv(16)*** *vs t(8;21)*<br>prognosis*
HOXA9	11	4	35	*MLL fusion gene*<br>*11q23*<br>*NPM1 mutation*<br>*normal cytogenetics*	*CEBPA mutation*<br>*t(8;21)*<br>*abnormal cytogenetics*<br>*inv(16)*<br>*t(15;17)*<br>*control*	***AML*** *vs ALL*
HOXB5	9	5	32	*NRAS-PM*<br>*FLT3-ITD*<br>*FLT3-TKD*<br>*normal cytogenetics*<br>*NPM1 mutation*	*CEBPA mutation*<br>*11q23*<br>*t(8;21)*<br>*inv(16)*<br>*t(15;17)*	***poor prognosis*** *vs good prognosis*<br>***euploid*** *vs aneuploid*
HOXA10	9	4	34	*MLL fusion gene*<br>*NPM1 mutation*<br>*normal cytogenetics*	*CEBPA mutation*<br>*abnormal cytogenetics*<br>*inv(16)*<br>*t(15;17)*<br>*control*	***poor prognosis*** *vs good prognosis*<br>***inv(16)*** *vs. t(15;17)*
MEIS1	9	3	16	*MLL fusion gene*<br>*NPM1 mutation*<br>*normal cytogenetics*<br>*good prognosis*	*CEBPA mutation*<br>*t(15;17)*	***inv(16)*** *vs t(8;21)*
HOXA4	8	4	18	*MLL fusion gene*<br>*11q23*<br>*NPM1 mutation*<br>*normal cytogenetics*	*t(8;21)*<br>*t(15;17)*<br>*inv(16)*	***poor prognosis*** *vs good prognosis*
HOXA5	6	1	15	*MLL fusion gene*<br>*NPM1 mutation*<br>*FLT3-ITD*	*inv(16)*<br>*t(15;17)*<br>*control*	
HOXB6	5	2	7	*NPM1 mutation*<br>*FLT3-ITD*		
HOXA7	5	1	12	*MLL fusion gene*<br>*NPM1 mutation*	*inv(16)*<br>*t(15;17)*<br>*control*	
PBX1	3	3	3	*t(11;19)*<br>*poor prognosis*		
HOXA2	3	2	4	*NPM1 mutation*<br>*normal cytogenetics*	*t(15;17)*	
HOXA6	3	1	6	*MLL fusion gene*<br>*NPM1 mutation*		
HOXB3	3	1	3	*NPM1 mutation*		
PBX2	3	1	3		*MLL fusion gene*<br>*NPM1 mutation*	
HOXB4	2	2	4	*NPM1 mutation*<br>*FLT3-ITD*<br>*normal cytogenetics*		
HOXB9	2	1	4	*MLL fusion gene*<br>*NPM1 mutation*		
HOXA1	1	1	2	*NPM1 mutation*		
HOXB7	1	1	2	*NPM1 mutation*		
HOXA11	1	1	1	*NPM1 mutation*		
HOXA3	1	1	1	*NPM1 mutation*		
HOXC4	1	1	1		*NPM1 mutation*	
HOXC6	1	1	1			
HOXD4	1	1	1	*NPM1 mutation*		

Listing of all HOX/TALE family genes in our analysis. Gene symbols are approved by HUGO Gene Nomenclature Committee. Identification tag descriptions can be found in Table S1.

*Contradictory prognosis expression directions.
