# Supplementary material for: Integrative Meta-Analysis of Differential Gene Expression in Acute Myeloid Leukemia
Source: PLoS One. 2010 Mar 1;5(3):e9466. doi: 10.1371/journal.pone.0009466 (PMC2830886; doi:10.1371/journal.pone.0009466)
Supplement: Table S4 — Top ranked genes associated with good prognosis (0.03 MB PDF) [file pone.0009466.s004.pdf]

Table S4. Top ranked genes associated with good prognosis

Rank	Gene symbol	No. of specific references	Total no. of references	Total no. of platforms	Total no. of differentially expressed features	Gene name
**Up-regulated genes**						
1	EML4	4	4	3	22	echinoderm microtubule associated protein like 4
2	SMG1*	3	5	4	26	phosphatidylinositol 3-kinase-related protein kinase
3	FOXO1	3	5	4	15	forkhead box O1
4	IL6ST	3	4	3	18	interleukin 6 signal transducer (gp130, oncostatin M receptor)
5	UGCG	3	4	3	12	UDP-glucose ceramide glucosyltransferase
6	ADFP	3	4	2	12	adipose differentiation-related protein
7	TP53BP2	3	3	3	5	tumor protein p53 binding protein, 2
8	SNX9	3	3	2	14	sorting nexin 9
9	PIK3R4	3	3	2	8	phosphoinositide-3-kinase, regulatory subunit 4, p150
10	SEMA3F	3	3	2	6	sema domain, immunoglobulin domain (Ig), short basic domain, secreted, (semaphorin) 3F
11	VCAN	2	8	5	22	versican
12	JAG1	2	8	3	20	jagged 1 (Alagille syndrome)
13	CD3D	2	7	4	21	CD3d molecule, delta (CD3-TCR complex)
14	SLC7A7	2	6	5	14	solute carrier family 7 (cationic amino acid transporter, y+ system), member 7
15	ENDOD1	2	6	4	13	endonuclease domain containing 1
16	TNFAIP2	2	6	4	12	tumor necrosis factor, alpha-induced protein 2
17	GYPC	2	6	4	11	glycophorin C (Gerbich blood group)
18	ISG20	2	6	3	17	interferon stimulated exonuclease gene 20kDa
19	CAT	2	6	3	11	catalase
20	C3AR1	2	6	2	9	complement component 3a receptor 1
**Down-regulated genes**						
1	BCL11A	3	5	4	19	B-cell CLL/lymphoma 11A (zinc finger protein)
2	TBXAS1	3	5	4	11	thromboxane A synthase 1 (platelet, cytochrome P450, family 5, subfamily A)
3	HOXB5	2	9	5	32	homeobox B5
4	HOXA10	2	9	4	34	homeobox A10
5	CD34	2	9	3	16	CD34 molecule
6	HOXA4	2	8	4	18	homeobox A4
7	SKAP2	2	5	4	21	src kinase associated phosphoprotein 2
8	MCM3	2	5	4	9	minichromosome maintenance complex component 3
9	DAPK1	2	5	3	8	death-associated protein kinase 1
10	GUCY1A3	2	4	4	8	guanylate cyclase 1, soluble, alpha 3
11	CDK6	2	4	3	12	cyclin-dependent kinase 6
12	ANGPT1	2	4	3	11	angiopoietin 1
13	MTHFD1	2	4	3	6	methylenetetrahydrofolate dehydrogenase (NADP+ dependent) 1, methenyltetrahydrofolate cyclohydrolase,
14	MAP7	2	3	3	14	microtubule-associated protein 7
15	UGCGL2	2	3	3	11	UDP-glucose ceramide glucosyltransferase-like 2
16	FLT3	2	3	3	5	fms-related tyrosine kinase 3
17	HADHSC* (HADH)	2	3	3	5	hydroxyacyl-Coenzyme A dehydrogenase
18	TCF4	2	3	2	19	transcription factor 4
19	NME1	2	3	2	8	non-metastatic cells 1, protein (NM23A) expressed in
20	POLR2H	2	3	2	8	polymerase (RNA) II (DNA directed) polypeptide H

In order of preference, the genes are ranked by the number of good prognosis related independent studies, the total number of independent studies, the total number of unique platforms, and the total number of features. Genes that were also associated with poor prognosis with the same expression direction are not shown.

*Gene symbol is not approved by HUGO Gene Nomenclature Committee. If a HUGO approved name does exist, it is placed in parenthesis.
