# Supplementary material for: Integrative Meta-Analysis of Differential Gene Expression in Acute Myeloid Leukemia
Source: PLoS One. 2010 Mar 1;5(3):e9466. doi: 10.1371/journal.pone.0009466 (PMC2830886; doi:10.1371/journal.pone.0009466)
Supplement: Table S5 — Functional categories of up-regulated genes associated with poor prognosis (0.02 MB PDF) [file pone.0009466.s005.pdf]

Table S5. Functional categories of up-regulated genes associated with poor prognosis

GO category	Corrected p-value	No. of genes	Other tags with upregulated genes	Other tags with downregulated genes
**Biological Processes**				
cell cycle	2.49E-15	50	*abnormal cytogenetics*<br>*aneuploid*<br>*CD34+CD38+ fraction*<br>*high centrosome aberrations*	*CD34+CD38- fraction*<br>*euploid*<br>*FLT3-ITD*<br>*low centrosome aberrations*<br>*MLL fusion gene*
cell cycle process	8.04E-12	33	*aneuploid*<br>*CD34+CD38+ fraction*<br>*high centrosome aberrations*	*CD34+CD38- fraction*<br>*euploid*<br>*FLT3-ITD*<br>*good prognosis*<br>*low centrosome aberrations*<br>*NPM1 mutation*
mitotic cell cycle	6.61E-10	26	*aneuploid*<br>*CD34+CD38+ fraction*<br>*high centrosome aberrations*	*CD34+CD38- fraction*<br>*euploid*<br>*FLT3-ITD*<br>*good prognosis*<br>*low centrosome aberrations*<br>*NPM1 mutation*
cell cycle phase	6.37E-09	26	*aneuploid*<br>*CD34+CD38+ fraction*<br>*high centrosome aberrations*	*CD34+CD38- fraction*<br>*euploid*<br>*FLT3-ITD*<br>*good prognosis*<br>*low centrosome aberrations*<br>*NPM1 mutation*
cell division	2.18E-08	21	*aneuploid*<br>*CD34+CD38+ fraction*<br>*high centrosome aberrations*	*CD34+CD38- fraction*<br>*euploid*<br>*good prognosis*<br>*low centrosome aberrations*<br>*NPM1 mutation*
mitosis	4.00E-08	20	*aneuploid*<br>*CD34+CD38+ fraction*	*CD34+CD38- fraction*<br>*euploid*<br>*FLT3-ITD*<br>*good prognosis*<br>*NPM1 mutation*
M phase of mitotic cell cycle	6.37E-08	20	*aneuploid*<br>*CD34+CD38+ fraction*	*CD34+CD38- fraction*<br>*euploid*<br>*FLT3-ITD*<br>*good prognosis*<br>*NPM1 mutation*
regulation of cell cycle	2.91E-07	21	*abnormal cytogenetics*<br>*aneuploid*<br>*CD34+CD38+ fraction*<br>*high centrosome aberrations*	*CD34+CD38- fraction*<br>*euploid*<br>*good prognosis*<br>*low centrosome aberrations*
M phase	8.37E-07	21	*aneuploid*<br>*CD34+CD38+ fraction*	*CD34+CD38- fraction*<br>*euploid*<br>*good prognosis*<br>*NPM1 mutation*
negative regulation of transcription	3.84E-06	20	*good prognosis*	
negative regulation of nucleobase, nucleoside, nucleotide and nucleic acid metabolic process	4.31E-06	21	*good prognosis*	
interphase of mitotic cell cycle	1.90E-04	10	*aneuploid*<br>*CD34+CD38+ fraction*<br>*high centrosome aberrations*	*CD34+CD38- fraction*<br>*euploid*<br>*good prognosis*<br>*low centrosome aberrations*<br>*NPM1 mutation*
regulation of cellular component organization and biogenesis	3.20E-04	17		
interphase	3.70E-04	10	*aneuploid*<br>*CD34+CD38+ fraction*<br>*high centrosome aberrations*	*CD34+CD38- fraction*<br>*euploid*<br>*good prognosis*<br>*low centrosome aberrations*<br>*NPM1 mutation*
organelle organization and biogenesis	1.47E-03	45	*aneuploid*	*euploid*

G1 phase of mitotic cell cycle	1.85E-03	5	*CD34+CD38+ fraction*	*CD34+CD38- fraction*<br>*good prognosis*
regulation of organelle organization and biogenesis	1.95E-03	10		
organic acid metabolic process	1.95E-03	28		*good prognosis*
microtubule-based process	2.00E-03	19		
DNA metabolic process	2.46E-03	30	*abnormal cytogenetics*<br>*aneuploid*<br>*high centrosome aberrations*	*euploid*<br>*good prognosis*<br>*low centrosome aberrations*
G1 phase	4.49E-03	5	*CD34+CD38+ fraction*	*CD34+CD38- fraction*<br>*good prognosis*
carboxylic acid metabolic process	5.34E-03	27		*good prognosis*
regulation of cytoskeleton organization and biogenesis	5.74E-03	9		
**Cellular Components**				
microtubule cytoskeleton	3.65E-06	26	*aneuploid*<br>*CD34+CD38+ fraction*	*CD34+CD38- fraction*<br>*euploid*<br>*good prognosis*
spindle	2.68E-05	10	*aneuploid*<br>*CD34+CD38+ fraction*	*CD34+CD38- fraction*<br>*euploid*<br>*good prognosis*<br>*NPM1 mutation*
protein complex	8.99E-05	73	*aneuploid*<br>*high centrosome aberrations*	*euploid*<br>*good prognosis*<br>*low centrosome aberrations*
mitochondrion	3.30E-04	34	*MLL fusion gene*	*good prognosis*
non-membrane-bound organelle	2.30E-03	68	*aneuploid*	*euploid*
intracellular non-membrane-bound organelle	2.30E-03	68	*aneuploid*	*euploid*
plasma membrane part	7.59E-03	54	*11q23*<br>*CBF*<br>*CEBPA silenced*<br>*FAB-M4*<br>*FAB-M5*<br>*FAB-M7*<br>*inv(16)*	*CEBPA mutation*<br>*t(15;17)*<br>*good prognosis*
microtubule organizing center	7.77E-03	10	*aneuploid*<br>*CD34+CD38+ fraction*	*CD34+CD38- fraction*<br>*euploid*<br>*good prognosis*
organelle outer membrane	9.09E-03	8		

Significantly over-represented functional gene ontology (GO) categories of up-regulated genes associated with poor prognosis are presented here. GO categories that are also over-represented in down-regulated genes associated with poor prognosis are not included. Corrected p-value is the Bonferroni multiple hypothesis. Identification tags that are both up-regulated and down-regulated are not included in the 'other tags' columns. Identification tag descriptions can be found in Table S1.
