# Supplementary material for: Integrative Meta-Analysis of Differential Gene Expression in Acute Myeloid Leukemia
Source: PLoS One. 2010 Mar 1;5(3):e9466. doi: 10.1371/journal.pone.0009466 (PMC2830886; doi:10.1371/journal.pone.0009466)
Supplement: Table S6 — Functional categories of down-regulated genes associated with poor prognosis (0.03 MB PDF) [file pone.0009466.s006.pdf]

Table S6. Functional categories of down-regulated genes associated with poor prognosis

GO category	Corrected p-value	No. of genes	Other tags with downregulated genes	Other tags with upregulated genes
**Biological Processes**				
protein kinase cascade	7.86E-15	44		*good prognosis*<br>*MLL fusion gene*<br>*NPM1 mutation*
intracellular signaling cascade	1.96E-14	114	*t(8;21)*	*good prognosis*<br>*inv(16)*<br>*MLL fusion gene*<br>*NPM1 mutation*
transcription from RNA polymerase II promoter	1.88E-12	54	*del(7q)*<br>*MLL fusion gene*<br>*t(8;21)*	*abnormal cytogenetics*<br>*good prognosis*<br>*inv(16)*<br>*NPM1 mutation*
signal transduction	3.97E-12	211	*CD34+CD38- fraction*<br>*CEBPA mutation*<br>*FLT3 mutation*<br>*t(8;21)*	*CD34+CD38+ fraction*<br>*good prognosis*<br>*inv(16)*<br>*normal cytogenetics*
negative regulation of developmental process	7.73E-12	37	*FLT3 mutation*	*good prognosis*<br>*NPM1 mutation*
cell communication	9.13E-12	222	*CD34+CD38- fraction*<br>*CEBPA mutation*<br>*t(8;21)*	*CD34+CD38+ fraction*<br>*CEBPA silenced*<br>*good prognosis*<br>*inv(16)*<br>*normal cytogenetics*
negative regulation of apoptosis	3.74E-10	29		*good prognosis*<br>*NPM1 mutation*
negative regulation of programmed cell death	5.23E-10	29		*good prognosis*<br>*NPM1 mutation*
positive regulation of metabolic process	2.46E-09	38	*low centrosome aberrations*	*good prognosis*<br>*high centrosome aberrations*<br>*inv(16)*
RNA metabolic process	3.74E-09	171		*abnormal cytogenetics*<br>*good prognosis*<br>*NPM1 mutation*
macromolecule localization	3.61E-08	64		*good prognosis*<br>*MLL fusion gene*
protein localization	9.25E-08	61		*good prognosis*<br>*MLL fusion gene*
regulation of cell proliferation	1.06E-07	38	*CD34+CD38+ fraction*<br>*euploid*<br>*t(8;21)*	*aneuploid*<br>*CD34+CD38- fraction*<br>*good prognosis*<br>*MLL fusion gene*<br>*NPM1 mutation*
nucleobase, nucleoside, nucleotide and nucleic acid metabolic process	1.43E-07	208		*abnormal cytogenetics*<br>*good prognosis*<br>*NPM1 mutation*
regulation of metabolic process	1.95E-07	165	*CEBPA mutation*<br>*low centrosome aberrations*<br>*t(15;17)*	*good prognosis*<br>*high centrosome aberrations*<br>*NPM1 mutation*
regulation of cellular metabolic process	2.25E-07	161	*CEBPA mutation*<br>*low centrosome aberrations*<br>*t(15;17)*	*good prognosis*<br>*high centrosome aberrations*<br>*NPM1 mutation*
multi-organism process	1.25E-06	29	*CD34+CD38- fraction*	*CD34+CD38+ fraction*<br>*FAB-M4*<br>*FAB-M5*<br>*good prognosis*<br>*NPM1 mutation*
positive regulation of transcription, DNA-dependent	1.32E-06	24		*good prognosis*
positive regulation of RNA metabolic process	1.58E-06	24		*good prognosis*

positive regulation of transcription	1.63E-06	27	*low centrosome aberrations*	*good prognosis*<br>*high centrosome aberrations*
protein transport	2.15E-06	54		*good prognosis*<br>*MLL fusion gene*
cellular localization	2.75E-06	64		*good prognosis*<br>*MLL fusion gene*
response to external stimulus	3.41E-06	43	*CD34+CD38- fraction*<br>*control*<br>*del(7q)*<br>*t(8;21)*	*CD34+CD38+ fraction*<br>*good prognosis*<br>*inv(16)*<br>*MLL fusion gene*<br>*monocytic*<br>*normal cytogenetics*<br>*NPM1 mutation*
positive regulation of nucleobase, nucleoside, nucleotide and nucleic acid metabolic process	4.04E-06	27	*low centrosome aberrations*	*good prognosis*<br>*high centrosome aberrations*<br>*inv(16)*
establishment of protein localization	5.13E-06	55		*good prognosis*<br>*MLL fusion gene*
anti-apoptosis	7.03E-06	20		*good prognosis*<br>*NPM1 mutation*
regulation of gene expression	8.13E-06	149	*abnormal cytogenetics*<br>*CEBPA mutation*<br>*t(15;17)*	*good prognosis*<br>*NPM1 mutation*
ubiquitin cycle	1.09E-05	38		*good prognosis*
localization	1.17E-05	172	*t(8;21)*	*FAB-M7*<br>*good prognosis*<br>*inv(16)*<br>*MLL fusion gene*
anatomical structure morphogenesis	2.44E-05	50	*t(15;17)*<br>*t(8;21)*	*FLT3-ITD*<br>*FLT3 mutation*<br>*good prognosis*<br>*MLL fusion gene*<br>*normal cytogenetics*
regulation of transcription, DNA-dependent	2.75E-05	132	*abnormal cytogenetics*<br>*CEBPA mutation*	*good prognosis*
enzyme linked receptor protein signaling pathway	3.16E-05	26		*good prognosis*
regulation of RNA metabolic process	3.61E-05	132	*abnormal cytogenetics*<br>*CEBPA mutation*	*good prognosis*
transcription, DNA-dependent	3.71E-05	134	*abnormal cytogenetics*<br>*CEBPA mutation*	*good prognosis*<br>*NPM1 mutation*
transcription	3.95E-05	145	*abnormal cytogenetics*<br>*CEBPA mutation*	*good prognosis*<br>*NPM1 mutation*
response to molecule of bacterial origin	4.09E-05	6		*good prognosis*<br>*NPM1 mutation*
RNA biosynthetic process	4.18E-05	134	*abnormal cytogenetics*<br>*CEBPA mutation*	*good prognosis*<br>*NPM1 mutation*
hemopoietic or lymphoid organ development	4.27E-05	19	*NPM1 mutation*	*good prognosis*<br>*inv(16)*
gene expression	4.54E-05	191		*abnormal cytogenetics*<br>*good prognosis*
hemopoiesis	7.32E-05	18	*NPM1 mutation*	*good prognosis*<br>*inv(16)*
regulation of nucleobase, nucleoside, nucleotide and nucleic acid metabolic process	7.96E-05	141	*abnormal cytogenetics*<br>*CEBPA mutation*	*good prognosis*<br>*NPM1 mutation*
establishment of cellular localization	8.87E-05	59		*good prognosis*<br>*MLL fusion gene*
immune system development	9.70E-05	19		*good prognosis*<br>*inv(16)*
response to other organism	1.20E-04	20	*CD34+CD38- fraction*	*CD34+CD38+ fraction*<br>*FAB-M4*<br>*FAB-M5*<br>*good prognosis*<br>*NPM1 mutation*

response to wounding	1.30E-04	31	*CD34+CD38- fraction*<br>*control*<br>*t(8;21)*	*CD34+CD38+ fraction*<br>*good prognosis*<br>*inv(16)*<br>*MLL fusion gene*<br>*monocytic*<br>*normal cytogenetics*<br>*NPM1 mutation*
mRNA metabolic process	1.40E-04	27		*good prognosis*
regulation of transcription	1.40E-04	138	*abnormal cytogenetics*<br>*CEBPA mutation*	*good prognosis*<br>*NPM1 mutation*
positive regulation of developmental process	1.50E-04	27	*t(8;21)*	*good prognosis*<br>*inv(16)*<br>*MLL fusion gene*
regulation of signal transduction	1.50E-04	46		*good prognosis*
vesicle-mediated transport	1.80E-04	38	*t(8;21)*	*inv(16)*<br>*MLL fusion gene*
protein complex assembly	2.00E-04	27		
intracellular transport	2.30E-04	51		*good prognosis*
positive regulation of cell proliferation	2.80E-04	21	*t(8;21)*	*inv(16)*<br>*MLL fusion gene*<br>*NPM1 mutation*
mRNA processing	3.50E-04	23		*good prognosis*
localization of cell	3.70E-04	28	*CD34+CD38- fraction*<br>*normal cytogenetics*<br>*t(8;21)*	*CD34+CD38+ fraction*<br>*good prognosis*<br>*inv(16)*<br>*MLL fusion gene*<br>*NPM1 mutation*
cell motility	3.70E-04	28	*CD34+CD38- fraction*<br>*normal cytogenetics*<br>*t(8;21)*	*CD34+CD38+ fraction*<br>*good prognosis*<br>*inv(16)*<br>*MLL fusion gene*<br>*NPM1 mutation*
lipid metabolic process	4.10E-04	50		*MLL fusion gene*
positive regulation of signal transduction	4.60E-04	17		*good prognosis*<br>*MLL fusion gene*<br>*NPM1 mutation*
response to biotic stimulus	6.60E-04	23	*CD34+CD38- fraction*	*CD34+CD38+ fraction*<br>*good prognosis*<br>*NPM1 mutation*
response to hormone stimulus	7.60E-04	12		
regulation of biological quality	8.30E-04	55	*t(8;21)*	*FAB-M7*<br>*good prognosis*<br>*MLL fusion gene*<br>*t(8;14)*
organ morphogenesis	9.60E-04	26	*t(8;21)*	*good prognosis*<br>*inv(16)*<br>*normal cytogenetics*<br>*NPM1 mutation*
nervous system development	1.07E-03	40	*CD34+CD38+ fraction*<br>*inv(16)*<br>*NPM1 mutation*	*CD34+CD38- fraction*<br>*good prognosis*
protein targeting	1.97E-03	19		*good prognosis*
response to hypoxia	2.06E-03	9		*good prognosis*
response to endogenous stimulus	2.22E-03	33	*euploid*<br>*low centrosome aberrations*	*abnormal cytogenetics*<br>*aneuploid*<br>*good prognosis*<br>*high centrosome aberrations*
RNA splicing	3.14E-03	19		*good prognosis*
response to chemical stimulus	4.65E-03	34	*11q23*<br>*CD34+CD38- fraction*<br>*t(8;21)*	*CD34+CD38+ fraction*<br>*good prognosis*<br>*inv(16)*<br>*normal cytogenetics*<br>*NPM1 mutation*

defense response	4.99E-03	34	*CD34+CD38- fraction<br>t(8;21)*	*11q23<br>CD34+CD38+ fraction<br>good prognosis<br>inv(16)<br>MLL fusion gene<br>monocytic<br>normal cytogenetics*
I-kappaB kinase/NF-kappaB cascade	6.01E-03	14		*good prognosis<br>MLL fusion gene<br>NPM1 mutation*
membrane organization and biogenesis	6.46E-03	21	*CD34+CD38- fraction<br>NPM1 mutation<br>t(8;21)*	*CD34+CD38+ fraction<br>inv(16)<br>MLL fusion gene*
nuclear transport	6.53E-03	15		*good prognosis*
positive regulation of transcription from RNA polymerase II promoter	6.55E-03	14		*good prognosis*
mRNA transport	6.78E-03	11		*good prognosis*
immune response-regulating signal transduction	8.01E-03	7		*good prognosis*
cellular lipid metabolic process	8.15E-03	40		*MLL fusion gene*
**Molecular Functions**				
transcription factor binding	2.99E-10	39		*good prognosis<br>NPM1 mutation*
transcription cofactor activity	5.79E-09	31		*good prognosis*
transcription activator activity	1.22E-08	31	*t(8;21)*	*good prognosis<br>inv(16)<br>NPM1 mutation*
RNA binding	5.25E-08	59		*good prognosis*
transcription coactivator activity	7.18E-08	23	*t(8;21)*	*good prognosis<br>inv(16)*
transcription regulator activity	7.05E-05	91	*abnormal cytogenetics<br>control<br>MLL fusion gene<br>t(15;17)*	*good prognosis<br>NPM1 mutation*
receptor signaling protein activity	1.10E-04	18		*good prognosis<br>MLL fusion gene*
enzyme binding	1.30E-04	24		*good prognosis*
transcription repressor activity	1.59E-03	18		*good prognosis*
DNA binding	2.03E-03	127	*abnormal cytogenetics*	*good prognosis<br>NPM1 mutation*
RNA helicase activity	2.16E-03	7		
molecular adaptor activity	4.81E-03	9		*good prognosis*
protein serine/threonine kinase activity	7.93E-03	36	*euploid*	*aneuploid<br>good prognosis*
**Cellular Components**				
nuclear body	6.21E-09	19		*good prognosis*
Golgi apparatus	2.23E-08	49	*t(8;21)*	*good prognosis<br>inv(16)<br>MLL fusion gene*
organelle membrane	4.49E-08	80	*t(8;21)*	*MLL fusion gene*
nuclear speck	2.67E-07	15		*good prognosis*
endomembrane system	6.15E-07	59	*CEBPA mutation<br>t(8;21)*	*CEBPA silenced<br>good prognosis<br>inv(16)<br>MLL fusion gene<br>NPM1 mutation*
vacuole	2.30E-04	20	*CD34+CD38- fraction<br>CEBPA silenced<br>t(8;21)*	*CD34+CD38+ fraction<br>CEBPA mutation<br>FAB-M4<br>FAB-M5<br>good prognosis<br>inv(16)<br>MLL fusion gene<br>monocytic*

lysosome	4.40E-04	18	*CD34+CD38- fraction*<br>*CEBPA silenced*<br>*t(8;21)*	*CD34+CD38+ fraction*<br>*CEBPA mutation*<br>*FAB-M4*<br>*FAB-M5*<br>*good prognosis*<br>*inv(16)*<br>*MLL fusion gene*<br>*monocytic*
Golgi membrane	4.70E-04	26	*t(8;21)*	*inv(16)*
lytic vacuole	5.40E-04	18	*CD34+CD38- fraction*<br>*CEBPA silenced*<br>*t(8;21)*	*CD34+CD38+ fraction*<br>*CEBPA mutation*<br>*FAB-M4*<br>*FAB-M5*<br>*good prognosis*<br>*inv(16)*<br>*MLL fusion gene*<br>*monocytic*
endoplasmic reticulum	1.10E-03	45	*t(8;21)*	*CEBPA silenced*<br>*MLL fusion gene*
cellular_component	1.54E-03	40		*good prognosis*
endosome	1.94E-03	17		*good prognosis*<br>*MLL fusion gene*
Golgi apparatus part	2.10E-03	27	*t(8;21)*	*inv(16)*<br>*MLL fusion gene*

Significantly over-represented functional gene ontology (GO) categories of down-regulated genes associated with poor prognosis are presented here. GO categories that are also over-represented in up-regulated genes associated with poor prognosis are not included. Corrected p-value is the Bonferroni multiple hypothesis. Identification tags that are both up-regulated and down-regulated are not included in the 'other tags' columns. Identification tag descriptions can be found in Table S1.
