# Supplementary material for: Integrative Meta-Analysis of Differential Gene Expression in Acute Myeloid Leukemia
Source: PLoS One. 2010 Mar 1;5(3):e9466. doi: 10.1371/journal.pone.0009466 (PMC2830886; doi:10.1371/journal.pone.0009466)
Supplement: Table S7 — Functional categories of up-regulated genes associated with good prognosis (0.03 MB PDF) [file pone.0009466.s007.pdf]

Table S7. Functional categories of up-regulated genes associated with good prognosis

GO category	Corrected p-value	No. of genes	Other tags with upregulated genes	Other tags with downregulated genes
**Biological Processes**				
regulation of cellular process	6.66E-25	266	*aneuploid*<br>*CD34+CD38+ fraction*<br>*CEBPA silenced*<br>*high centrosome aberrations*<br>*inv(16)*<br>*MLL fusion gene*	*CD34+CD38- fraction*<br>*CEBPA mutation*<br>*euploid*<br>*FLT3 mutation*<br>*low centrosome aberrations*<br>*t(15;17)*<br>*t(8;21)*
positive regulation of biological process	5.00E-22	91	*aneuploid*<br>*CD34+CD38+ fraction*<br>*high centrosome aberrations*<br>*inv(16)*	*CD34+CD38- fraction*<br>*CEBPA mutation*<br>*euploid*<br>*low centrosome aberrations*<br>*t(8;21)*
positive regulation of cellular process	2.00E-21	86	*aneuploid*<br>*CD34+CD38+ fraction*<br>*high centrosome aberrations*<br>*inv(16)*<br>*MLL fusion gene*<br>*normal cytogenetics*	*CD34+CD38- fraction*<br>*euploid*<br>*low centrosome aberrations*<br>*t(8;21)*
transcription from RNA polymerase II promoter	9.14E-18	62	*abnormal cytogenetics*<br>*inv(16)*<br>*NPM1 mutation*	*del(7q)*<br>*MLL fusion gene*<br>*poor prognosis*<br>*t(8;21)*
regulation of developmental process	9.57E-17	70	*CD34+CD38+ fraction*<br>*CEBPA silenced*<br>*inv(16)*<br>*MLL fusion gene*	*CD34+CD38- fraction*<br>*CEBPA mutation*<br>*t(8;21)*
protein kinase cascade	9.58E-16	45	*MLL fusion gene*<br>*NPM1 mutation*	*poor prognosis*
multicellular organismal process	1.99E-15	174	*11q23*<br>*FAB-M4*<br>*FAB-M5*<br>*FAB-M7*<br>*FLT3-ITD*<br>*FLT3 mutation*	*CEBPA mutation*<br>*control*<br>*del(7q)*<br>*t(8;21)*
RNA metabolic process	1.42E-14	187	*abnormal cytogenetics*<br>*NPM1 mutation*	*poor prognosis*
intracellular signaling cascade	2.48E-14	113	*inv(16)*<br>*MLL fusion gene*<br>*NPM1 mutation*	*poor prognosis*<br>*t(8;21)*
signal transduction	7.34E-14	215	*CD34+CD38+ fraction*<br>*inv(16)*<br>*normal cytogenetics*	*CD34+CD38- fraction*<br>*CEBPA mutation*<br>*FLT3 mutation*<br>*poor prognosis*<br>*t(8;21)*
cell communication	8.89E-14	227	*CD34+CD38+ fraction*<br>*CEBPA silenced*<br>*inv(16)*<br>*normal cytogenetics*	*CD34+CD38- fraction*<br>*CEBPA mutation*<br>*poor prognosis*<br>*t(8;21)*
negative regulation of developmental process	1.79E-13	39	*NPM1 mutation*	*FLT3 mutation*<br>*poor prognosis*
nucleobase, nucleoside, nucleotide and nucleic acid metabolic process	2.75E-12	224	*abnormal cytogenetics*<br>*NPM1 mutation*	*poor prognosis*
regulation of apoptosis	4.30E-12	50	*CD34+CD38+ fraction*<br>*CEBPA silenced*<br>*MLL fusion gene*	*CD34+CD38- fraction*<br>*CEBPA mutation*<br>*t(8;21)*
regulation of transcription from RNA polymerase II promoter	6.61E-12	42	*normal cytogenetics*<br>*NPM1 mutation*	*CEBPA mutation*<br>*MLL fusion gene*<br>*t(8;21)*
regulation of programmed cell death	6.93E-12	50	*CEBPA silenced*<br>*MLL fusion gene*	*CEBPA mutation*<br>*t(8;21)*

positive regulation of metabolic process	1.94E-11	41	*high centrosome aberrations*<br>*inv(16)*	*low centrosome aberrations*<br>*poor prognosis*
regulation of metabolic process	3.07E-11	177	*high centrosome aberrations*<br>*NPM1 mutation*	*CEBPA mutation*<br>*low centrosome aberrations*<br>*poor prognosis*<br>*t(15;17)*
regulation of cellular metabolic process	3.39E-11	173	*high centrosome aberrations*<br>*NPM1 mutation*	*CEBPA mutation*<br>*low centrosome aberrations*<br>*poor prognosis*<br>*t(15;17)*
positive regulation of cellular metabolic process	3.49E-11	40	*high centrosome aberrations*<br>*inv(16)*	*low centrosome aberrations*
negative regulation of apoptosis	4.74E-11	30	*NPM1 mutation*	*poor prognosis*
negative regulation of programmed cell death	6.73E-11	30	*NPM1 mutation*	*poor prognosis*
regulation of gene expression	1.06E-09	162	*NPM1 mutation*	*abnormal cytogenetics*<br>*CEBPA mutation*<br>*poor prognosis*<br>*t(15;17)*
protein modification process	1.29E-09	126	*CD34+CD38- fraction*	*CD34+CD38+ fraction*
transcription	1.82E-09	160	*NPM1 mutation*	*abnormal cytogenetics*<br>*CEBPA mutation*<br>*poor prognosis*
transcription, DNA-dependent	2.48E-09	148	*NPM1 mutation*	*abnormal cytogenetics*<br>*CEBPA mutation*<br>*poor prognosis*
RNA biosynthetic process	2.89E-09	148	*NPM1 mutation*	*abnormal cytogenetics*<br>*CEBPA mutation*<br>*poor prognosis*
post-translational protein modification	3.96E-09	109	*CD34+CD38- fraction*	*CD34+CD38+ fraction*
regulation of RNA metabolic process	4.58E-09	145		*abnormal cytogenetics*<br>*CEBPA mutation*<br>*poor prognosis*
regulation of transcription, DNA-dependent	6.53E-09	144		*abnormal cytogenetics*<br>*CEBPA mutation*<br>*poor prognosis*
positive regulation of transcription, DNA-dependent	6.58E-09	27		*poor prognosis*
positive regulation of RNA metabolic process	8.12E-09	27		*poor prognosis*
gene expression	9.88E-09	205	*abnormal cytogenetics*	*poor prognosis*
positive regulation of transcription	1.20E-08	30	*high centrosome aberrations*	*low centrosome aberrations*<br>*poor prognosis*
anatomical structure morphogenesis	1.24E-08	57	*FLT3-ITD*<br>*FLT3 mutation*<br>*MLL fusion gene*<br>*normal cytogenetics*	*poor prognosis*<br>*t(15;17)*<br>*t(8;21)*
regulation of nucleobase, nucleoside, nucleotide and nucleic acid metabolic process	1.55E-08	154	*NPM1 mutation*	*abnormal cytogenetics*<br>*CEBPA mutation*<br>*poor prognosis*
positive regulation of developmental process	2.94E-08	33	*inv(16)*<br>*MLL fusion gene*	*poor prognosis*<br>*t(8;21)*
regulation of biological quality	2.96E-08	66	*FAB-M7*<br>*MLL fusion gene*<br>*t(8;14)*	*poor prognosis*<br>*t(8;21)*
regulation of transcription	3.22E-08	151	*NPM1 mutation*	*abnormal cytogenetics*<br>*CEBPA mutation*<br>*poor prognosis*
positive regulation of nucleobase, nucleoside, nucleotide and nucleic acid metabolic process	3.42E-08	30	*high centrosome aberrations*<br>*inv(16)*	*low centrosome aberrations*<br>*poor prognosis*

organ morphogenesis	2.86E-07	32	*inv(16)*<br>*normal cytogenetics*<br>*NPM1 mutation*	*poor prognosis*<br>*t(8;21)*
mRNA metabolic process	5.21E-07	31		*poor prognosis*
multi-organism process	1.04E-06	29	*CD34+CD38+ fraction*<br>*FAB-M4*<br>*FAB-M5*<br>*NPM1 mutation*	*CD34+CD38- fraction*<br>*poor prognosis*
macromolecule localization	1.27E-06	60	*MLL fusion gene*	*poor prognosis*
mRNA processing	4.26E-06	26		*poor prognosis*
hemopoietic or lymphoid organ development	6.90E-06	20	*inv(16)*	*NPM1 mutation*<br>*poor prognosis*
protein localization	8.61E-06	56	*MLL fusion gene*	*poor prognosis*
ubiquitin cycle	8.72E-06	38		*poor prognosis*
hemopoiesis	1.16E-05	19	*inv(16)*	*NPM1 mutation*<br>*poor prognosis*
regulation of cell proliferation	1.40E-05	34	*aneuploid*<br>*CD34+CD38- fraction*<br>*MLL fusion gene*<br>*NPM1 mutation*	*CD34+CD38+ fraction*<br>*euploid*<br>*poor prognosis*<br>*t(8;21)*
immune system development	1.66E-05	20	*inv(16)*	*poor prognosis*
negative regulation of transcription	1.68E-05	26	*poor prognosis*	
regulation of signal transduction	1.73E-05	48		*poor prognosis*
response to other organism	2.34E-05	21	*CD34+CD38+ fraction*<br>*FAB-M4*<br>*FAB-M5*<br>*NPM1 mutation*	*CD34+CD38- fraction*<br>*poor prognosis*
localization	2.71E-05	169	*FAB-M7*<br>*inv(16)*<br>*MLL fusion gene*	*poor prognosis*<br>*t(8;21)*
protein transport	2.92E-05	51	*MLL fusion gene*	*poor prognosis*
anti-apoptosis	3.48E-05	19	*NPM1 mutation*	*poor prognosis*
negative regulation of nucleobase, nucleoside, nucleotide and nucleic acid metabolic process	3.69E-05	27	*poor prognosis*	
response to molecule of bacterial origin	4.08E-05	6	*NPM1 mutation*	*poor prognosis*
establishment of protein localization	6.24E-05	52	*MLL fusion gene*	*poor prognosis*
cellular localization	6.34E-05	60	*MLL fusion gene*	*poor prognosis*
localization of cell	9.26E-05	29	*CD34+CD38+ fraction*<br>*inv(16)*<br>*MLL fusion gene*<br>*NPM1 mutation*	*CD34+CD38- fraction*<br>*normal cytogenetics*<br>*poor prognosis*<br>*t(8;21)*
cell motility	9.26E-05	29	*CD34+CD38+ fraction*<br>*inv(16)*<br>*MLL fusion gene*<br>*NPM1 mutation*	*CD34+CD38- fraction*<br>*normal cytogenetics*<br>*poor prognosis*<br>*t(8;21)*
positive regulation of cell activation	1.40E-04	11	*NPM1 mutation*	
positive regulation of leukocyte activation	1.40E-04	11	*NPM1 mutation*	
response to biotic stimulus	1.50E-04	24	*CD34+CD38+ fraction*<br>*NPM1 mutation*	*CD34+CD38- fraction*<br>*poor prognosis*
RNA splicing	1.60E-04	21		*poor prognosis*
regulation of immune effector process	1.70E-04	8	*normal cytogenetics*<br>*NPM1 mutation*	
response to hypoxia	1.80E-04	10		*poor prognosis*
response to external stimulus	1.90E-04	39	*CD34+CD38+ fraction*<br>*inv(16)*<br>*MLL fusion gene*<br>*monocytic*<br>*normal cytogenetics*<br>*NPM1 mutation*	*CD34+CD38- fraction*<br>*control*<br>*del(7q)*<br>*poor prognosis*<br>*t(8;21)*

positive regulation of transcription from RNA polymerase II promoter	2.10E-04	16		*poor prognosis*
positive regulation of apoptosis	3.10E-04	22	*NPM1 mutation*	
regulation of mast cell cytokine production	3.80E-04	4	*inv(16)*<br>*normal cytogenetics*	*t(8;21)*
positive regulation of programmed cell death	3.80E-04	22	*NPM1 mutation*	
mast cell cytokine production	3.80E-04	4	*inv(16)*<br>*normal cytogenetics*	*t(8;21)*
enzyme linked receptor protein signaling pathway	4.10E-04	24		*poor prognosis*
protein targeting	4.20E-04	20		*poor prognosis*
positive regulation of signal transduction	4.20E-04	17	*MLL fusion gene*<br>*NPM1 mutation*	*poor prognosis*
regulation of molecular function	5.20E-04	40	*aneuploid*<br>*high centrosome aberrations*	*euploid*<br>*low centrosome aberrations*<br>*NPM1 mutation*
induction of apoptosis	7.10E-04	19	*NPM1 mutation*	
positive regulation of lymphocyte activation	7.10E-04	10	*NPM1 mutation*	
establishment of cellular localization	7.50E-04	56	*MLL fusion gene*	*poor prognosis*
induction of programmed cell death	7.60E-04	19	*NPM1 mutation*	
cytokine production	8.30E-04	14	*CD34+CD38+ fraction*<br>*FAB-M4*<br>*FAB-M5*<br>*inv(16)*<br>*NPM1 mutation*	*CD34+CD38- fraction*<br>*t(8;21)*
mRNA transport	9.50E-04	12		*poor prognosis*
regulation of production of molecular mediator of immune response	1.04E-03	5	*normal cytogenetics*<br>*NPM1 mutation*	
regulation of cytokine production during immune response	1.04E-03	5	*normal cytogenetics*<br>*NPM1 mutation*	
homeostasis of number of cells	1.13E-03	9		
positive regulation of T cell activation	1.13E-03	9	*NPM1 mutation*	
cell activation	1.18E-03	19	*inv(16)*<br>*NPM1 mutation*	*t(8;21)*
nuclear transport	1.26E-03	16		*poor prognosis*
regulation of cell activation	1.57E-03	12	*inv(16)*<br>*NPM1 mutation*	*t(8;21)*
regulation of leukocyte activation	1.57E-03	12	*inv(16)*<br>*NPM1 mutation*	*t(8;21)*
defense response	1.58E-03	35	*11q23*<br>*CD34+CD38+ fraction*<br>*inv(16)*<br>*MLL fusion gene*<br>*monocytic*<br>*normal cytogenetics*	*CD34+CD38- fraction*<br>*poor prognosis*<br>*t(8;21)*
response to endogenous stimulus	1.87E-03	33	*abnormal cytogenetics*<br>*aneuploid*<br>*high centrosome aberrations*	*euploid*<br>*low centrosome aberrations*<br>*poor prognosis*
peptidyl-amino acid modification	2.07E-03	14		
blood vessel development	2.46E-03	16	*inv(16)*<br>*NPM1 mutation*	*MLL fusion gene*<br>*t(8;21)*
regulation of immune system process	2.46E-03	16	*inv(16)*<br>*NPM1 mutation*	*t(8;21)*
positive regulation of immune system process	3.02E-03	14	*inv(16)*<br>*NPM1 mutation*	*t(8;21)*
regulation of T cell activation	3.06E-03	10	*NPM1 mutation*	
vasculature development	3.12E-03	16	*inv(16)*<br>*NPM1 mutation*	*MLL fusion gene*<br>*t(8;21)*
nucleic acid transport	3.18E-03	12		
RNA transport	3.18E-03	12		

establishment of RNA localization	3.18E-03	12		
response to wounding	3.42E-03	28	*CD34+CD38+ fraction*<br>*inv(16)*<br>*MLL fusion gene*<br>*monocytic*<br>*normal cytogenetics*<br>*NPM1 mutation*	*CD34+CD38- fraction*<br>*control*<br>*poor prognosis*<br>*t(8;21)*
protein import into nucleus	3.55E-03	12		
RNA localization	3.55E-03	12		
T cell activation	3.55E-03	12	*inv(16)*	
response to chemical stimulus	3.92E-03	34	*CD34+CD38+ fraction*<br>*inv(16)*<br>*normal cytogenetics*<br>*NPM1 mutation*	*11q23*<br>*CD34+CD38- fraction*<br>*poor prognosis*<br>*t(8;21)*
regulation of multicellular organismal process	4.18E-03	23	*FAB-M7*<br>*inv(16)*<br>*NPM1 mutation*	*t(8;21)*
regulation of response to stimulus	4.42E-03	15	*NPM1 mutation*	*t(8;21)*
nuclear import	4.43E-03	12		
leukocyte mediated immunity	4.56E-03	11	*inv(16)*<br>*normal cytogenetics*<br>*NPM1 mutation*	*t(8;21)*
immune response-regulating cell surface receptor signaling pathway	4.92E-03	7		
intracellular transport	5.14E-03	47		*poor prognosis*
negative regulation of cell cycle	5.19E-03	14		
nucleocytoplasmic transport	5.20E-03	15		
nervous system development	5.45E-03	38	*CD34+CD38- fraction*	*CD34+CD38+ fraction*<br>*inv(16)*<br>*NPM1 mutation*<br>*poor prognosis*
I-kappaB kinase/NF-kappaB cascade	5.66E-03	14	*MLL fusion gene*<br>*NPM1 mutation*	*poor prognosis*
regulation of catalytic activity	5.96E-03	35	*aneuploid*<br>*high centrosome aberrations*	*euploid*<br>*low centrosome aberrations*<br>*NPM1 mutation*
regulation of cell differentiation	6.60E-03	15	*NPM1 mutation*	
response to abiotic stimulus	6.64E-03	16		
regulation of cytokine production	6.75E-03	8	*normal cytogenetics*<br>*NPM1 mutation*	
RNA processing	7.00E-03	31		
immune response-regulating signal transduction	7.95E-03	7		*poor prognosis*
**Molecular Functions**				
binding	1.20E-14	619	*aneuploid*<br>*CD34+CD38+ fraction*<br>*inv(16)*<br>*normal cytogenetics*	*CD34+CD38- fraction*<br>*euploid*<br>*t(8;21)*
transcription factor binding	1.65E-14	45	*NPM1 mutation*	*poor prognosis*
transcription activator activity	2.40E-12	36	*inv(16)*<br>*NPM1 mutation*	*poor prognosis*<br>*t(8;21)*
transcription cofactor activity	3.09E-11	34		*poor prognosis*
RNA binding	1.42E-09	62		*poor prognosis*
transcription coactivator activity	1.44E-09	25	*inv(16)*	*poor prognosis*<br>*t(8;21)*
transcription regulator activity	6.81E-09	103	*NPM1 mutation*	*abnormal cytogenetics*<br>*control*<br>*MLL fusion gene*<br>*poor prognosis*<br>*t(15;17)*
DNA binding	5.13E-07	141	*NPM1 mutation*	*abnormal cytogenetics*<br>*poor prognosis*
transcription repressor activity	3.28E-06	22		*poor prognosis*
enzyme binding	2.86E-05	25		*poor prognosis*

receptor signaling protein activity	9.93E-05	18	*MLL fusion gene*	*poor prognosis*
nucleic acid binding	1.90E-03	203		*abnormal cytogenetics*
RNA polymerase II transcription factor activity	2.58E-03	21		
protein serine/threonine kinase activity	2.63E-03	37	*aneuploid*	*euploid*<br>*poor prognosis*
nuclear hormone receptor binding	2.86E-03	9		
hormone receptor binding	3.31E-03	9		
molecular adaptor activity	4.41E-03	9		*poor prognosis*
protein dimerization activity	5.74E-03	24	*CD34+CD38+ fraction*	*CD34+CD38- fraction*
metal ion binding	6.20E-03	212		
transcription factor activity	7.24E-03	63	*NPM1 mutation*	*abnormal cytogenetics*<br>*control*<br>*MLL fusion gene*<br>*t(15;17)*

Cellular Components

nucleoplasm part	7.90E-13	45		
nuclear body	5.45E-10	20		*poor prognosis*
nuclear speck	2.21E-08	16		*poor prognosis*
Golgi apparatus	9.93E-06	43	*inv(16)*<br>*MLL fusion gene*	*poor prognosis*<br>*t(8;21)*
endomembrane system	3.52E-05	54	*CEBPA silenced*<br>*inv(16)*<br>*MLL fusion gene*<br>*NPM1 mutation*	*CEBPA mutation*<br>*poor prognosis*<br>*t(8;21)*
lysosome	8.17E-05	19	*CD34+CD38+ fraction*<br>*CEBPA mutation*<br>*FAB-M4*<br>*FAB-M5*<br>*inv(16)*<br>*MLL fusion gene*<br>*monocytic*	*CD34+CD38- fraction*<br>*CEBPA silenced*<br>*poor prognosis*<br>*t(8;21)*
lytic vacuole	1.00E-04	19	*CD34+CD38+ fraction*<br>*CEBPA mutation*<br>*FAB-M4*<br>*FAB-M5*<br>*inv(16)*<br>*MLL fusion gene*<br>*monocytic*	*CD34+CD38- fraction*<br>*CEBPA silenced*<br>*poor prognosis*<br>*t(8;21)*
vacuole	1.80E-04	20	*CD34+CD38+ fraction*<br>*CEBPA mutation*<br>*FAB-M4*<br>*FAB-M5*<br>*inv(16)*<br>*MLL fusion gene*<br>*monocytic*	*CD34+CD38- fraction*<br>*CEBPA silenced*<br>*poor prognosis*<br>*t(8;21)*
cellular_component	1.13E-03	40		*poor prognosis*
intrinsic to plasma membrane	1.19E-03	59	*CBF*<br>*CD34+CD38+ fraction*<br>*FAB-M4*<br>*FAB-M5*<br>*FAB-M7*<br>*inv(16)*<br>*monocytic*	*CD34+CD38- fraction*<br>*t(15;17)*
integral to plasma membrane	1.67E-03	58	*CBF*<br>*CD34+CD38+ fraction*<br>*FAB-M4*<br>*FAB-M5*<br>*FAB-M7*<br>*inv(16)*<br>*monocytic*	*CD34+CD38- fraction*
endosome	6.26E-03	16	*MLL fusion gene*	*poor prognosis*

plasma membrane	6.87E-03	129	*11q23*<br>*CBF*<br>*CD34+CD38+ fraction*<br>*CEBPA silenced*<br>*control*<br>*FAB-M4*<br>*FAB-M5*<br>*FAB-M7*<br>*inv(16)*<br>*monocytic*	*CD34+CD38- fraction*<br>*CEBPA mutation*<br>*t(15;17)*

Significantly over-represented functional gene ontology (GO) categories of up-regulated genes associated with good prognosis are presented here. GO categories that are also over-represented in down-regulated genes associated with good prognosis are not included. Corrected p-value is the Bonferroni multiple hypothesis. Identification tags that are both up-regulated and down-regulated are not included in the 'other tags' columns. Identification tag descriptions can be found in Table S1.
