# Supplementary material for: Integrative Meta-Analysis of Differential Gene Expression in Acute Myeloid Leukemia
Source: PLoS One. 2010 Mar 1;5(3):e9466. doi: 10.1371/journal.pone.0009466 (PMC2830886; doi:10.1371/journal.pone.0009466)
Supplement: Table S8 — Functional categories of down-regulated genes associated with good prognosis (0.02 MB PDF) [file pone.0009466.s008.pdf]

Table S8. Functional categories of down-regulated genes associated with good prognosis

GO category	Corrected p-value	No. of genes	Other tags with downregulated genes	Other tags with upregulated genes
**Biological Processes**				
cell cycle process	2.20E-11	31	*CD34+CD38- fraction*<br>*euploid*<br>*FLT3-ITD*<br>*low centrosome aberrations*<br>*NPM1 mutation*	*aneuploid*<br>*CD34+CD38+ fraction*<br>*high centrosome aberrations*<br>*poor prognosis*
mitotic cell cycle	3.64E-09	24	*CD34+CD38- fraction*<br>*euploid*<br>*FLT3-ITD*<br>*low centrosome aberrations*<br>*NPM1 mutation*	*aneuploid*<br>*CD34+CD38+ fraction*<br>*high centrosome aberrations*<br>*poor prognosis*
cell cycle phase	4.75E-09	25	*CD34+CD38- fraction*<br>*euploid*<br>*FLT3-ITD*<br>*low centrosome aberrations*<br>*NPM1 mutation*	*aneuploid*<br>*CD34+CD38+ fraction*<br>*high centrosome aberrations*<br>*poor prognosis*
cell division	2.77E-08	20	*CD34+CD38- fraction*<br>*euploid*<br>*low centrosome aberrations*<br>*NPM1 mutation*	*aneuploid*<br>*CD34+CD38+ fraction*<br>*high centrosome aberrations*<br>*poor prognosis*
regulation of cell cycle	4.93E-08	21	*CD34+CD38- fraction*<br>*euploid*<br>*low centrosome aberrations*	*abnormal cytogenetics*<br>*aneuploid*<br>*CD34+CD38+ fraction*<br>*high centrosome aberrations*<br>*poor prognosis*
mitosis	5.63E-08	19	*CD34+CD38- fraction*<br>*euploid*<br>*FLT3-ITD*<br>*NPM1 mutation*	*aneuploid*<br>*CD34+CD38+ fraction*<br>*poor prognosis*
M phase of mitotic cell cycle	8.78E-08	19	*CD34+CD38- fraction*<br>*euploid*<br>*FLT3-ITD*<br>*NPM1 mutation*	*aneuploid*<br>*CD34+CD38+ fraction*<br>*poor prognosis*
M phase	9.13E-07	20	*CD34+CD38- fraction*<br>*euploid*<br>*NPM1 mutation*	*aneuploid*<br>*CD34+CD38+ fraction*<br>*poor prognosis*
DNA metabolic process	3.28E-05	32	*euploid*<br>*low centrosome aberrations*	*abnormal cytogenetics*<br>*aneuploid*<br>*high centrosome aberrations*<br>*poor prognosis*
interphase of mitotic cell cycle	7.51E-05	10	*CD34+CD38- fraction*<br>*euploid*<br>*low centrosome aberrations*<br>*NPM1 mutation*	*aneuploid*<br>*CD34+CD38+ fraction*<br>*high centrosome aberrations*<br>*poor prognosis*
interphase	1.40E-04	10	*CD34+CD38- fraction*<br>*euploid*<br>*low centrosome aberrations*<br>*NPM1 mutation*	*aneuploid*<br>*CD34+CD38+ fraction*<br>*high centrosome aberrations*<br>*poor prognosis*
organic acid metabolic process	2.90E-04	28		*poor prognosis*
carboxylic acid metabolic process	8.70E-04	27		*poor prognosis*
G1 phase of mitotic cell cycle	1.04E-03	5	*CD34+CD38- fraction*	*CD34+CD38+ fraction*<br>*poor prognosis*
G1 phase	2.54E-03	5	*CD34+CD38- fraction*	*CD34+CD38+ fraction*<br>*poor prognosis*
cellular component assembly	4.19E-03	27		
**Molecular Functions**				
nucleotide binding	6.42E-03	84	*euploid*	*aneuploid*
**Cellular Components**				
microtubule cytoskeleton	2.41E-06	25	*CD34+CD38- fraction*<br>*euploid*	*aneuploid*<br>*CD34+CD38+ fraction*<br>*poor prognosis*

mitochondrion	4.30E-06	36		*MLL fusion gene*<br>*poor prognosis*
spindle	1.10E-05	10	*CD34+CD38- fraction*<br>*euploid*<br>*NPM1 mutation*	*aneuploid*<br>*CD34+CD38+ fraction*<br>*poor prognosis*
protein complex	2.29E-05	70	*euploid*<br>*low centrosome aberrations*	*aneuploid*<br>*high centrosome aberrations*<br>*poor prognosis*
plasma membrane part	1.16E-03	53	*CEBPA mutation*<br>*t(15;17)*	*11q23*<br>*CEBPA silenced*<br>*FAB-M4*<br>*FAB-M5*<br>*FAB-M7*<br>*inv(16)*<br>*poor prognosis*
microtubule organizing center	3.45E-03	10	*CD34+CD38- fraction*<br>*euploid*	*aneuploid*<br>*CD34+CD38+ fraction*<br>*poor prognosis*
macromolecular complex	3.51E-03	78	*euploid*<br>*low centrosome aberrations*	*abnormal cytogenetics*<br>*aneuploid*<br>*high centrosome aberrations*
cytoskeleton	3.77E-03	38	*euploid*<br>*NPM1 mutation*	*aneuploid*

Significantly over-represented functional gene ontology (GO) categories of down-regulated genes associated with good prognosis are presented here. GO categories that are also over-represented in up-regulated genes associated with good prognosis are not included. Corrected p-value is the Bonferroni multiple hypothesis. Identification tags that are both up-regulated and down-regulated are not included in the 'other tags' columns. Identification tag descriptions can be found in Table S1.
