# Supplementary material for: Integrative Meta-Analysis of Differential Gene Expression in Acute Myeloid Leukemia
Source: PLoS One. 2010 Mar 1;5(3):e9466. doi: 10.1371/journal.pone.0009466 (PMC2830886; doi:10.1371/journal.pone.0009466)
Supplement: Table S9 — Top ranked genes associated with NPM1 mutations (0.03 MB PDF) [file pone.0009466.s009.pdf]

Table S9. Top ranked genes associated with *NPM1* mutations

Rank	Gene symbol	no. of specific references	Total no. of references	Total no. of platforms	Total no. of differentially expressed features	Gene name
**Up-regulated genes**						
1	SMC4	5	5	2	15	structural maintenance of chromosomes 4
2	PBX3	4	12	5	31	pre-B-cell leukemia homeobox 3
3	MEIS1	4	9	3	16	Meis homeobox 1
4	CAT	4	6	3	11	catalase
5	COL4A5	4	6	3	10	collagen, type IV, alpha 5 (Alport syndrome)
6	FOXC1	4	6	3	7	forkhead box C1
7	LTBP1	4	4	2	9	latent transforming growth factor beta binding protein 1
8	FTO	4	4	2	5	fat mass and obesity associated
9	HOXB2	3	12	6	32	homeobox B2
10	HOXA9	3	11	4	35	homeobox A9
11	HOXB5	3	9	5	32	homeobox B5
12	HOXA10	3	9	4	34	homeobox A10
13	HOXA4	3	8	4	18	homeobox A4
14	SCHIP1	3	7	3	8	schwannomin interacting protein 1
15	HOXA5	3	6	1	15	homeobox A5
16	SNX10	3	5	2	8	sorting nexin 10
17	HOXB6	3	5	2	7	homeobox B6
18	HOXA7	3	5	1	12	homeobox A7
19	RASGRP3	3	4	2	8	RAS guanyl releasing protein 3 (calcium and DAG-regulated)
20	IRX5	3	4	2	5	iroquois homeobox 5
**Down-regulated genes**						
1	HLA-DPA1	4	10	3	20	major histocompatibility complex, class II, DP alpha 1
2	CD34	4	9	3	16	CD34 molecule
3	SPARC	3	9	5	16	secreted protein, acidic, cysteine-rich (osteonectin)
4	HLA-DRA	3	6	3	12	major histocompatibility complex, class II, DR alpha
5	IFITM1	3	6	3	9	interferon induced transmembrane protein 1 (9-27)
6	SERPINF1	3	6	3	8	serpin peptidase inhibitor, clade F (alpha-2 antiplasmin, pigment epithelium derived factor), member 1
7	APP	3	6	2	9	amyloid beta (A4) precursor protein (peptidase nexin-II, Alzheimer disease)
8	MOX2* (CD200)	3	5	3	9	CD200 molecule
9	CD7	3	5	2	16	CD7 molecule
10	PMAIP1	3	5	2	8	phorbol-12-myristate-13-acetate-induced protein 1
11	MHC2TA* (CIITA)	3	5	2	6	class II, major histocompatibility complex, transactivator
12	EGFL7	3	5	1	6	EGF-like-domain, multiple 7
13	P2RY5	3	4	2	7	purinergic receptor P2Y, G-protein coupled, 5
14	EVL	3	4	2	6	Enah/Vasp-like
15	VEGFA	3	3	1	3	vascular endothelial growth factor A
16	POU4F1	2	11	3	29	POU class 4 homeobox 1
17	TSPAN7	2	10	5	16	tetraspanin 7
18	RUNX1T1	2	10	3	34	runt-related transcription factor 1; translocated to, 1 (cyclin D-related)
19	TRH	2	10	3	21	thyrotropin-releasing hormone
20	MN1	2	8	4	16	meningioma (disrupted in balanced translocation) 1

In order of preference, the genes are ranked by the number of *NPM1* mutations related independent studies, the total number of independent studies, the total number of unique platforms, and the total number of features.

*Gene symbol is not approved by HUGO Gene Nomenclature Committee. If a HUGO approved name does exist, it is placed in parenthesis.
