# Supplementary material for: Integrative Meta-Analysis of Differential Gene Expression in Acute Myeloid Leukemia
Source: PLoS One. 2010 Mar 1;5(3):e9466. doi: 10.1371/journal.pone.0009466 (PMC2830886; doi:10.1371/journal.pone.0009466)
Supplement: Table S10 — Top ranked genes associated with t(15;17) (0.01 MB PDF) [file pone.0009466.s010.pdf]

Table S10. Top ranked genes associated with t(15;17)

Rank	Gene symbol	no. of specific references	Total no. of references	Total no. of platforms	Total no. of differentially expressed features	Gene name
**Up-regulated genes**						
1	CST7	4	7	5	8	cystatin F (leukocystatin)
2	HGF	4	7	2	36	hepatocyte growth factor (hepapoietin A; scatter factor)
3	CALR	4	5	4	11	calreticulin
4	PRODH	4	5	3	8	proline dehydrogenase (oxidase) 1
5	ANXA8	4	5	2	9	annexin A8
6	FGF13	4	5	2	8	fibroblast growth factor 13
7	CTSW	3	6	3	15	cathepsin W
8	PTGDS	3	4	2	13	prostaglandin D2 synthase 21kDa (brain)
9	S100B	3	4	2	7	S100 calcium binding protein B
10	MST1	3	4	2	6	macrophage stimulating 1 (hepatocyte growth factor-like)
11	P4HB	3	4	2	6	procollagen-proline, 2-oxoglutarate 4-dioxygenase (proline 4-hydroxylase), beta polypeptide
12	FP504	3	4	1	7	maternally expressed 3
13	AGRN	2	5	4	12	agrin
14	SERPING1	2	4	3	10	serpin peptidase inhibitor, clade G (C1 inhibitor), member 1, (angioedema, hereditary)
15	CPA3	2	4	3	5	carboxypeptidase A3 (mast cell)
16	GABRE	2	4	3	4	gamma-aminobutyric acid (GABA) A receptor, epsilon
17	KRT18	2	4	2	11	keratin 18
18	IGFBP2	2	4	2	6	insulin-like growth factor binding protein 2, 36kDa
19	PCBP3	2	3	3	4	poly(rC) binding protein 3
20	FAM45B	2	3	2	8	family with sequence similarity 45, member B
**Down-regulated genes**						
1	ARHGAP4	7	7	3	23	Rho GTPase activating protein 4
2	CLEC2B	4	4	3	14	C-type lectin domain family 2, member B
3	HOXA9	3	11	4	35	homeobox A9
4	HLA-DPA1	3	10	3	20	major histocompatibility complex, class II, DP alpha 1
5	NRIP1	3	4	2	12	nuclear receptor interacting protein 1
6	PRDX4	3	3	3	11	peroxiredoxin 4
7	RGS10	2	8	3	17	regulator of G-protein signaling 10
8	ITGB2	2	6	4	17	integrin, beta 2 (complement component 3 receptor 3 and 4 subunit)
9	CD52	2	6	3	14	CD52 molecule
10	SKAP2	2	5	4	21	src kinase associated phosphoprotein 2
11	HLA-DMA	2	5	3	7	major histocompatibility complex, class II, DM alpha
12	SELL	2	4	4	9	selectin L (lymphocyte adhesion molecule 1)
13	ADD3	2	3	3	5	adducin 3 (gamma)
14	LGALS9	2	3	3	3	lectin, galactoside-binding, soluble, 9 (galectin 9)

In order of preference, the genes are ranked by the number of t(15;17) related independent studies, the total number of independent studies, the total number of unique platforms, and the total number of features. Gene symbols are approved by HUGO Gene Nomenclature Committee.
