# Supplementary material for: Integrative Meta-Analysis of Differential Gene Expression in Acute Myeloid Leukemia
Source: PLoS One. 2010 Mar 1;5(3):e9466. doi: 10.1371/journal.pone.0009466 (PMC2830886; doi:10.1371/journal.pone.0009466)
Supplement: Table S11 — Top ranked genes associated with inv(16) (0.03 MB PDF) [file pone.0009466.s011.pdf]

## Table S11. Top ranked genes associated with inv(16)

| Rank | Gene symbol | no. of specific references | Total no. of references | Total no. of platforms | Total no. of differentially expressed features | Gene name |
|---|---|---|---|---|---|---|
| **Up-regulated genes** | | | | | | |
| 1 | MYH11 | 7 | 10 | 3 | 38 | myosin, heavy chain 11, smooth muscle |
| 2 | TGFBI | 5 | 7 | 4 | 13 | transforming growth factor, beta-induced, 68kDa |
| 3 | PTPRM | 5 | 6 | 3 | 11 | protein tyrosine phosphatase, receptor type, M |
| 4 | SPARC | 4 | 9 | 5 | 16 | secreted protein, acidic, cysteine-rich (osteonectin) |
| 5 | MN1 | 4 | 8 | 4 | 16 | meningioma (disrupted in balanced translocation) 1 |
| 6 | CD52 | 4 | 6 | 3 | 14 | CD52 molecule |
| 7 | CD1C | 4 | 6 | 3 | 7 | CD1c molecule |
| 8 | CHI3L1 | 4 | 6 | 2 | 13 | chitinase 3-like 1 (cartilage glycoprotein-39) |
| 9 | ST18 | 4 | 6 | 2 | 7 | suppression of tumorigenicity 18 (breast carcinoma) (zinc finger protein) |
| 10 | NRP1 | 4 | 5 | 3 | 9 | neuropilin 1 |
| 11 | NPTN | 3 | 7 | 3 | 11 | neuroplastin |
| 12 | SLC7A7 | 3 | 6 | 5 | 14 | solute carrier family 7 (cationic amino acid transporter, y+ system), member 7 |
| 13 | ITGB2 | 3 | 6 | 4 | 17 | integrin, beta 2 (complement component 3 receptor 3 and 4 subunit) |
| 14 | NT5E | 3 | 6 | 4 | 12 | 5'-nucleotidase, ecto (CD73) |
| 15 | PLXNB2 | 3 | 6 | 3 | 8 | plexin B2 |
| 16 | MSLN | 3 | 5 | 3 | 14 | mesothelin |
| 17 | SLC38A1 | 3 | 5 | 2 | 15 | solute carrier family 38, member 1 |
| 18 | SELL | 3 | 4 | 4 | 9 | selectin L (lymphocyte adhesion molecule 1) |
| 19 | MTMR11 | 3 | 4 | 2 | 7 | myotubularin related protein 11 |
| 20 | VSIG4 | 3 | 4 | 2 | 6 | V-set and immunoglobulin domain containing 4 |
| 21 | DUSP6 | 3 | 3 | 2 | 11 | dual specificity phosphatase 6 |
| 22 | FLJ22662* | 3 | 3 | 2 | 5 | hypothetical protein FLJ22662 |
| **Down-regulated genes** | | | | | | |
| 1 | RUNX3 | 4 | 8 | 5 | 23 | runt-related transcription factor 3 |
| 2 | CBFB | 3 | 5 | 3 | 9 | core-binding factor, beta subunit |
| 3 | CLIP2 | 3 | 5 | 3 | 8 | CAP-GLY domain containing linker protein 2 |
| 4 | CHST12 | 3 | 4 | 3 | 5 | carbohydrate (chondroitin 4) sulfotransferase 12 |
| 5 | MGLL | 3 | 3 | 2 | 4 | monoglyceride lipase |
| 6 | HOXA9 | 2 | 11 | 4 | 35 | homeobox A9 |
| 7 | POU4F1 | 2 | 11 | 3 | 29 | POU class 4 homeobox 1 |
| 8 | HOXA4 | 2 | 8 | 4 | 18 | homeobox A4 |
| 9 | ZHX2 | 2 | 4 | 3 | 5 | zinc fingers and homeoboxes 2 |
| 10 | SLPI | 2 | 3 | 2 | 10 | secretory leukocyte peptidase inhibitor |
| 11 | ABCA5 | 2 | 3 | 2 | 7 | ATP-binding cassette, sub-family A (ABC1), member 5 |
| 12 | CD81 | 2 | 3 | 2 | 6 | CD81 molecule |
| 13 | PELI2 | 2 | 3 | 2 | 6 | pellino homolog 2 (Drosophila) |
| 14 | PVR | 2 | 3 | 2 | 4 | poliovirus receptor |
| 15 | NDE1 | 2 | 2 | 2 | 5 | nudE nuclear distribution gene E homolog 1 (A. nidulans) |
| 16 | BAHCC1 | 2 | 2 | 2 | 4 | BAH domain and coiled-coil containing 1 |
| 17 | FBN2 | 2 | 2 | 1 | 3 | fibrillin 2 (congenital contractural arachnodactyly) |
| 18 | GLCCI1 | 2 | 2 | 1 | 3 | glucocorticoid induced transcript 1 |
| 19 | OAT | 2 | 2 | 1 | 3 | ornithine aminotransferase (gyrate atrophy) |
| 20 | SLC37A1 | 2 | 2 | 1 | 3 | solute carrier family 37 (glycerol-3-phosphate transporter), member 1 |

In order of preference, the genes are ranked by the number of inv(16) related independent studies, the total number of independent studies, the total number of unique platforms, and the total number of features.

*Gene symbol is not approved by HUGO Gene Nomenclature Committee.
