# Supplementary material for: Integrative Meta-Analysis of Differential Gene Expression in Acute Myeloid Leukemia
Source: PLoS One. 2010 Mar 1;5(3):e9466. doi: 10.1371/journal.pone.0009466 (PMC2830886; doi:10.1371/journal.pone.0009466)
Supplement: Table S12 — Top ranked genes associated with t(8;21) (0.03 MB PDF) [file pone.0009466.s012.pdf]

Table S12. Top ranked genes associated with t(8;21)

Rank	Gene symbol	no. of specific references	Total no. of references	Total no. of platforms	Total no. of differentially expressed features	Gene name
**Up-regulated genes**						
1	POU4F1	7	11	3	29	POU class 4 homeobox 1
2	RUNX1T1	6	10	3	34	runt-related transcription factor 1; translocated to, 1 (cyclin D-related)
3	TRH	6	10	3	21	thyrotropin-releasing hormone
4	RGS10	5	8	3	17	regulator of G-protein signaling 10
5	PRAME	4	8	4	11	preferentially expressed antigen in melanoma
6	CAV1	4	7	3	14	caveolin 1, caveolae protein, 22kDa
7	CACNA2D2	4	6	3	9	calcium channel, voltage-dependent, alpha 2/delta subunit 2
8	C11orf9	4	6	3	8	chromosome 11 open reading frame 9
9	TSPAN7	3	10	5	16	tetraspanin 7
10	PALM	3	7	4	9	paralemmin
11	GPM6B	3	7	3	16	glycoprotein M6B
12	BAIAP3	3	6	3	8	BAI1-associated protein 3
13	ITGB4	3	5	3	8	integrin, beta 4
14	ROBO1	3	5	3	8	roundabout, axon guidance receptor, homolog 1 (Drosophila)
15	HSPG2	3	5	3	7	heparan sulfate proteoglycan 2
16	SCML2	3	4	4	5	sex comb on midleg-like 2 (Drosophila)
17	JMJD2B	3	4	3	10	jumonji domain containing 2B
18	SLC25A1	3	4	3	9	solute carrier family 25 (mitochondrial carrier; citrate transporter), member 1
19	PSD3	3	4	3	8	pleckstrin and Sec7 domain containing 3
20	NCALD	3	4	3	6	neurocalcin delta
21	SLCO4A1	3	4	3	6	solute carrier organic anion transporter family, member 4A1
**Down-regulated genes**						
1	CTSW	4	6	3	15	cathepsin W
2	LCP1	4	5	2	8	lymphocyte cytosolic protein 1 (L-plastin)
3	CAPG	3	7	3	13	capping protein (actin filament), gelsolin-like
4	PLXNB2	3	6	3	8	plexin B2
5	LAPTM5	3	5	3	9	lysosomal associated multispanning membrane protein 5
6	WBSCR5* (LAT2)	3	4	2	5	linker for activation of T cells family, member 2
7	ECOP*	3	3	2	4	EGFR-coamplified and overexpressed protein
8	HOXB2	2	12	6	32	homeobox B2
9	MYH11	2	10	3	38	myosin, heavy chain 11, smooth muscle
10	JAG1	2	8	3	20	jagged 1 (Alagille syndrome)
11	TGFBI	2	7	4	13	transforming growth factor, beta-induced, 68kDa
12	SCHIP1	2	7	3	8	schwannomin interacting protein 1
13	ITGB2	2	6	4	17	integrin, beta 2 (complement component 3 receptor 3 and 4 subunit)
14	TNFAIP2	2	6	4	12	tumor necrosis factor, alpha-induced protein 2
15	PRKCD	2	6	4	9	protein kinase C, delta
16	ALDH2	2	6	4	8	aldehyde dehydrogenase 2 family (mitochondrial)
17	ABR	2	6	3	9	active BCR-related gene
18	ADCY7	2	5	3	8	adenylate cyclase 7
19	SERPING1	2	4	3	10	serpin peptidase inhibitor, clade G (C1 inhibitor), member 1, (angioedema, hereditary)
20	PELI1	2	4	3	5	pellino homolog 1 (Drosophila)

In order of preference, the genes are ranked by the number of t(8;21) related independent studies, the total number of independent studies, the total number of unique platforms, and the total number of features.

*Gene symbol is not approved by HUGO Gene Nomenclature Committee. If a HUGO approved name does exist, it is placed in parenthesis.
