# Supplementary material for: Integrative Meta-Analysis of Differential Gene Expression in Acute Myeloid Leukemia
Source: PLoS One. 2010 Mar 1;5(3):e9466. doi: 10.1371/journal.pone.0009466 (PMC2830886; doi:10.1371/journal.pone.0009466)
Supplement: Table S13 — Functional categories of up-regulated genes associated with NPM1 mutations (0.03 MB PDF) [file pone.0009466.s013.pdf]

Table S13. Functional categories of up-regulated genes associated with *NPM1* mutations

GO category	Corrected p-value	No. of genes	Other tags with upregulated genes	Other tags with downregulated genes
**Biological Processes**				
response to external stimulus	1.76E-20	53	*CD34+CD38+ fraction*<br>*good prognosis*<br>*inv(16)*<br>*MLL fusion gene*<br>*monocytic*<br>*normal cytogenetics*	*CD34+CD38- fraction*<br>*control*<br>*del(7q)*<br>*poor prognosis*<br>*t(8;21)*
response to wounding	3.59E-20	44	*CD34+CD38+ fraction*<br>*good prognosis*<br>*inv(16)*<br>*MLL fusion gene*<br>*monocytic*<br>*normal cytogenetics*	*CD34+CD38- fraction*<br>*control*<br>*poor prognosis*<br>*t(8;21)*
inflammatory response	3.68E-14	31	*CD34+CD38+ fraction*<br>*inv(16)*<br>*MLL fusion gene*<br>*monocytic*	*CD34+CD38- fraction*<br>*t(8;21)*
response to chemical stimulus	4.87E-12	40	*CD34+CD38+ fraction*<br>*good prognosis*<br>*inv(16)*<br>*normal cytogenetics*	*11q23*<br>*CD34+CD38- fraction*<br>*poor prognosis*<br>*t(8;21)*
taxis	6.36E-12	21	*CD34+CD38+ fraction*<br>*inv(16)*<br>*MLL fusion gene*	*CD34+CD38- fraction*<br>*control*
chemotaxis	6.36E-12	21	*CD34+CD38+ fraction*<br>*inv(16)*<br>*MLL fusion gene*	*CD34+CD38- fraction*<br>*control*
locomotory behavior	6.65E-11	22	*CD34+CD38+ fraction*<br>*inv(16)*	*11q23*<br>*CD34+CD38- fraction*<br>*control*<br>*t(8;21)*
immune effector process	2.54E-10	18	*inv(16)*	*t(8;21)*
negative regulation of developmental process	1.10E-09	27	*good prognosis*	*FLT3 mutation*<br>*poor prognosis*
positive regulation of multicellular organismal process	2.97E-09	19	*inv(16)*	*t(8;21)*
behavior	4.33E-09	25	*CD34+CD38+ fraction*<br>*inv(16)*	*CD34+CD38- fraction*
positive regulation of immune system process	1.15E-08	17	*good prognosis*<br>*inv(16)*	*t(8;21)*
adaptive immune response based on somatic recombination of immune receptors built from immunoglobulin superfamily domains	1.25E-08	14		*t(8;21)*
adaptive immune response	1.25E-08	14		*t(8;21)*
negative regulation of apoptosis	5.20E-08	21	*good prognosis*	*poor prognosis*
regulation of immune system process	6.47E-08	18	*good prognosis*<br>*inv(16)*	*t(8;21)*
negative regulation of programmed cell death	6.63E-08	21	*good prognosis*	*poor prognosis*
regulation of immune response	3.34E-07	14		*t(8;21)*
acute inflammatory response	3.40E-07	13		
cytokine production	2.45E-06	14	*CD34+CD38+ fraction*<br>*FAB-M4*<br>*FAB-M5*<br>*good prognosis*<br>*inv(16)*	*CD34+CD38- fraction*<br>*t(8;21)*
positive regulation of signal transduction	3.51E-06	16	*good prognosis*<br>*MLL fusion gene*	*poor prognosis*

cellular metabolic process	4.51E-06	255	*abnormal cytogenetics*<br>*high centrosome aberrations*<br>*MLL fusion gene*<br>*NRAS-PM*	*low centrosome aberrations*
positive regulation of immune response	5.56E-06	12		*t(8;21)*
localization of cell	7.59E-06	24	*CD34+CD38+ fraction*<br>*good prognosis*<br>*inv(16)*<br>*MLL fusion gene*	*CD34+CD38- fraction*<br>*normal cytogenetics*<br>*poor prognosis*<br>*t(8;21)*
cell motility	7.59E-06	24	*CD34+CD38+ fraction*<br>*good prognosis*<br>*inv(16)*<br>*MLL fusion gene*	*CD34+CD38- fraction*<br>*normal cytogenetics*<br>*poor prognosis*<br>*t(8;21)*
regulation of cytokine production	1.02E-05	9	*good prognosis*<br>*normal cytogenetics*	
production of molecular mediator of immune response	1.05E-05	8	*inv(16)*	*t(8;21)*
response to other organism	1.40E-05	17	*CD34+CD38+ fraction*<br>*FAB-M4*<br>*FAB-M5*<br>*good prognosis*	*CD34+CD38- fraction*<br>*poor prognosis*
transcription from RNA polymerase II promoter	1.51E-05	31	*abnormal cytogenetics*<br>*good prognosis*<br>*inv(16)*	*del(7q)*<br>*MLL fusion gene*<br>*poor prognosis*<br>*t(8;21)*
humoral immune response	1.59E-05	11	*inv(16)*	*t(8;21)*
regulation of cell proliferation	1.61E-05	26	*aneuploid*<br>*CD34+CD38- fraction*<br>*good prognosis*<br>*MLL fusion gene*	*CD34+CD38+ fraction*<br>*euploid*<br>*poor prognosis*<br>*t(8;21)*
regulation of cell differentiation	1.70E-05	15	*good prognosis*	
primary metabolic process	1.95E-05	253	*abnormal cytogenetics*<br>*normal cytogenetics*	*CEBPA mutation*
I-kappaB kinase/NF-kappaB cascade	1.96E-05	14	*good prognosis*<br>*MLL fusion gene*	*poor prognosis*
regulation of multicellular organismal process	2.56E-05	21	*FAB-M7*<br>*good prognosis*<br>*inv(16)*	*t(8;21)*
cytokine production during immune response	2.96E-05	6	*normal cytogenetics*	
regulation of myeloid cell differentiation	3.96E-05	8		
organ morphogenesis	3.98E-05	22	*good prognosis*<br>*inv(16)*<br>*normal cytogenetics*	*poor prognosis*<br>*t(8;21)*
leukocyte mediated immunity	4.15E-05	11	*good prognosis*<br>*inv(16)*<br>*normal cytogenetics*	*t(8;21)*
regulation of I-kappaB kinase/NF-kappaB cascade	5.64E-05	12	*MLL fusion gene*	
positive regulation of cytokine production	6.17E-05	7		
regulation of response to stimulus	8.07E-05	14	*good prognosis*	*t(8;21)*
activation of immune response	8.09E-05	10		*t(8;21)*
regulation of production of molecular mediator of immune response	8.79E-05	5	*good prognosis*<br>*normal cytogenetics*	
regulation of cytokine production during immune response	8.79E-05	5	*good prognosis*<br>*normal cytogenetics*	
protein kinase cascade	8.91E-05	22	*good prognosis*<br>*MLL fusion gene*	*poor prognosis*
positive regulation of response to stimulus	1.00E-04	12		
positive regulation of lymphocyte activation	1.20E-04	9	*good prognosis*	
lymphocyte mediated immunity	1.80E-04	10		*t(8;21)*

positive regulation of I-kappaB kinase/NF-kappaB cascade	1.90E-04	11	*MLL fusion gene*	
positive regulation of cellular biosynthetic process	2.00E-04	10		
positive regulation of biosynthetic process	2.00E-04	10		
intracellular signaling cascade	2.00E-04	61	*good prognosis*<br>*inv(16)*<br>*MLL fusion gene*	*poor prognosis*<br>*t(8;21)*
positive regulation of cell activation	2.20E-04	9	*good prognosis*	
positive regulation of leukocyte activation	2.20E-04	9	*good prognosis*	
regulation of cellular metabolic process	2.30E-04	102	*good prognosis*<br>*high centrosome aberrations*	*CEBPA mutation*<br>*low centrosome aberrations*<br>*poor prognosis*<br>*t(15;17)*
macromolecule metabolic process	3.30E-04	217	*abnormal cytogenetics*<br>*aneuploid*	*euploid*
regulation of nucleobase, nucleoside, nucleotide and nucleic acid metabolic process	3.70E-04	95	*good prognosis*	*abnormal cytogenetics*<br>*CEBPA mutation*<br>*poor prognosis*
nucleobase, nucleoside, nucleotide and nucleic acid metabolic process	4.20E-04	130	*abnormal cytogenetics*<br>*good prognosis*	*poor prognosis*
metabolic process	4.30E-04	278	*abnormal cytogenetics*	*euploid*
multi-organism process	4.50E-04	19	*CD34+CD38+ fraction*<br>*FAB-M4*<br>*FAB-M5*<br>*good prognosis*	*CD34+CD38- fraction*<br>*poor prognosis*
response to bacterium	5.40E-04	11	*CD34+CD38+ fraction*	*CD34+CD38- fraction*
regulation of metabolic process	5.40E-04	103	*good prognosis*<br>*high centrosome aberrations*	*CEBPA mutation*<br>*low centrosome aberrations*<br>*poor prognosis*<br>*t(15;17)*
regulation of cell activation	9.20E-04	10	*good prognosis*<br>*inv(16)*	*t(8;21)*
regulation of leukocyte activation	9.20E-04	10	*good prognosis*<br>*inv(16)*	*t(8;21)*
cell activation	9.70E-04	15	*good prognosis*<br>*inv(16)*	*t(8;21)*
regulation of body fluid levels	9.90E-04	12		*t(8;21)*
biopolymer metabolic process	1.22E-03	165	*abnormal cytogenetics*<br>*aneuploid*<br>*CD34+CD38- fraction*	*CD34+CD38+ fraction*<br>*euploid*
cellular defense response	1.30E-03	9	*CD34+CD38+ fraction*	*CD34+CD38- fraction*
positive regulation of translation	1.57E-03	8		
hemostasis	1.60E-03	11	*inv(16)*	*t(8;21)*
activation of plasma proteins during acute inflammatory response	1.64E-03	7		
complement activation	1.64E-03	7		
innate immune response	1.74E-03	11		
wound healing	1.82E-03	12	*inv(16)*	*t(8;21)*
transcription, DNA-dependent	1.90E-03	87	*good prognosis*	*abnormal cytogenetics*<br>*CEBPA mutation*<br>*poor prognosis*
regulation of adaptive immune response based on somatic recombination of immune receptors built from immunoglobulin superfamily domains	1.94E-03	5	*normal cytogenetics*	
regulation of transcription from RNA polymerase II promoter	1.94E-03	21	*good prognosis*<br>*normal cytogenetics*	*CEBPA mutation*<br>*MLL fusion gene*<br>*t(8;21)*
regulation of adaptive immune response	1.94E-03	5	*normal cytogenetics*	

transcription	1.99E-03	94	*good prognosis*	*abnormal cytogenetics*<br>*CEBPA mutation*<br>*poor prognosis*
response to biotic stimulus	2.04E-03	17	*CD34+CD38+ fraction*<br>*good prognosis*	*CD34+CD38- fraction*<br>*poor prognosis*
positive regulation of apoptosis	2.04E-03	16	*good prognosis*	
RNA biosynthetic process	2.06E-03	87	*good prognosis*	*abnormal cytogenetics*<br>*CEBPA mutation*<br>*poor prognosis*
anti-apoptosis	2.08E-03	13	*good prognosis*	*poor prognosis*
positive regulation of programmed cell death	2.38E-03	16	*good prognosis*	
regulation of immune effector process	2.47E-03	6	*good prognosis*<br>*normal cytogenetics*	
myeloid cell differentiation	2.59E-03	9		
regulation of DNA metabolic process	2.72E-03	8		
RNA metabolic process	2.77E-03	100	*abnormal cytogenetics*<br>*good prognosis*	*poor prognosis*
positive regulation of cell proliferation	2.82E-03	15	*inv(16)*<br>*MLL fusion gene*	*poor prognosis*<br>*t(8;21)*
positive regulation of cytokine biosynthetic process	3.27E-03	7		
induction of apoptosis	3.40E-03	14	*good prognosis*	
cell surface receptor linked signal transduction	3.54E-03	66		
regulation of lymphocyte activation	3.56E-03	9		
induction of programmed cell death	3.59E-03	14	*good prognosis*	
regulation of cytokine biosynthetic process	3.99E-03	8		*FLT3 mutation*
positive regulation of T cell activation	4.49E-03	7	*good prognosis*	
regulation of T cell activation	5.09E-03	8	*good prognosis*	
positive regulation of cellular protein metabolic process	6.44E-03	10		
regulation of transcription	6.76E-03	89	*good prognosis*	*abnormal cytogenetics*<br>*CEBPA mutation*<br>*poor prognosis*
blood coagulation	7.57E-03	10		*t(8;21)*
blood vessel development	7.58E-03	12	*good prognosis*<br>*inv(16)*	*MLL fusion gene*<br>*t(8;21)*
positive regulation of protein metabolic process	8.87E-03	10		
cytokine biosynthetic process	9.00E-03	8		*FLT3 mutation*
vasculature development	9.12E-03	12	*good prognosis*<br>*inv(16)*	*MLL fusion gene*<br>*t(8;21)*
regulation of gene expression	9.49E-03	92	*good prognosis*	*abnormal cytogenetics*<br>*CEBPA mutation*<br>*poor prognosis*<br>*t(15;17)*
response to molecule of bacterial origin	9.61E-03	4	*good prognosis*	*poor prognosis*
T-helper 2 type immune response	9.61E-03	4		
**Molecular Functions**				
sequence-specific DNA binding	2.33E-09	44	*FLT3-ITD*	*abnormal cytogenetics*<br>*control*<br>*t(15;17)*
DNA binding	6.02E-06	96	*good prognosis*	*abnormal cytogenetics*<br>*poor prognosis*
cytokine activity	2.90E-05	20	*CD34+CD38+ fraction*	*CD34+CD38- fraction*
transcription factor activity	4.20E-05	51	*good prognosis*	*abnormal cytogenetics*<br>*control*<br>*MLL fusion gene*<br>*t(15;17)*

chemokine activity	5.73E-05	10		*11q23*
chemokine receptor binding	6.52E-05	10		*11q23*
transcription activator activity	1.10E-04	19	*good prognosis*<br>*inv(16)*	*poor prognosis*<br>*t(8;21)*
receptor binding	1.10E-04	39	*11q23*<br>*inv(16)*	*t(8;21)*
transcription regulator activity	3.80E-04	62	*good prognosis*	*abnormal cytogenetics*<br>*control*<br>*MLL fusion gene*<br>*poor prognosis*<br>*t(15;17)*
G-protein-coupled receptor binding	1.16E-03	10		
transcription factor binding	8.59E-03	19	*good prognosis*	*poor prognosis*
**Cellular Components**				
extracellular space	1.15E-16	43	*11q23*<br>*CD34+CD38+ fraction*<br>*FLT3 mutation*<br>*inv(16)*	*CD34+CD38- fraction*<br>*t(8;21)*
extracellular region part	3.00E-13	51	*11q23*<br>*CD34+CD38+ fraction*<br>*FLT3 mutation*<br>*inv(16)*	*CD34+CD38- fraction*<br>*t(8;21)*
extracellular region	4.36E-09	80	*11q23*<br>*CD34+CD38+ fraction*<br>*inv(16)*<br>*t(15;17)*	*CD34+CD38- fraction*<br>*t(8;21)*
intracellular organelle part	1.54E-06	110	*abnormal cytogenetics*<br>*aneuploid*<br>*CD34+CD38+ fraction*<br>*CEBPA silenced*<br>*MLL fusion gene*<br>*normal cytogenetics*	*CD34+CD38- fraction*<br>*CEBPA mutation*<br>*euploid*
organelle part	1.75E-06	110	*abnormal cytogenetics*<br>*aneuploid*<br>*CD34+CD38+ fraction*<br>*CEBPA silenced*<br>*MLL fusion gene*<br>*normal cytogenetics*	*CD34+CD38- fraction*<br>*CEBPA mutation*<br>*euploid*
nuclear part	1.08E-03	38	*abnormal cytogenetics*<br>*aneuploid*<br>*normal cytogenetics*	*euploid*
DNA-directed RNA polymerase complex	1.59E-03	6		
nuclear DNA-directed RNA polymerase complex	1.59E-03	6		
chromosome	2.15E-03	23	*CD34+CD38+ fraction*	*CD34+CD38- fraction*
RNA polymerase complex	3.17E-03	6		
DNA-directed RNA polymerase II, core complex	3.66E-03	5		
chromosomal part	5.61E-03	20	*CD34+CD38+ fraction*	*CD34+CD38- fraction*
endomembrane system	6.45E-03	34	*CEBPA silenced*<br>*good prognosis*<br>*inv(16)*<br>*MLL fusion gene*	*CEBPA mutation*<br>*poor prognosis*<br>*t(8;21)*

Significantly over-represented functional gene ontology (GO) categories of up-regulated genes associated with *NPM1* mutations are presented here. GO categories that are also over-represented in down-regulated genes associated with *NPM1* mutations are not included. Corrected p-value is the Bonferroni multiple hypothesis. Identification tags that are both up-regulated and down-regulated are not included in the 'other tags' columns. Identification tag descriptions can be found in Table S1.
