# Supplementary material for: Integrative Meta-Analysis of Differential Gene Expression in Acute Myeloid Leukemia
Source: PLoS One. 2010 Mar 1;5(3):e9466. doi: 10.1371/journal.pone.0009466 (PMC2830886; doi:10.1371/journal.pone.0009466)
Supplement: Table S14 — Functional categories of down-regulated genes associated with NPM1 mutations (0.02 MB PDF) [file pone.0009466.s014.pdf]

Table S14. Functional categories of down-regulated genes associated with *NPM1* mutations

GO category	Corrected p-value	No. of genes	Other tags with downregulated genes	Other tags with upregulated genes
**Biological Processes**				
cell cycle process	2.63E-13	36	*CD34+CD38- fraction*<br>*euploid*<br>*FLT3-ITD*<br>*good prognosis*<br>*low centrosome aberrations*	*aneuploid*<br>*CD34+CD38+ fraction*<br>*high centrosome aberrations*<br>*poor prognosis*
cell cycle phase	4.63E-13	32	*CD34+CD38- fraction*<br>*euploid*<br>*FLT3-ITD*<br>*good prognosis*<br>*low centrosome aberrations*	*aneuploid*<br>*CD34+CD38+ fraction*<br>*high centrosome aberrations*<br>*poor prognosis*
mitotic cell cycle	1.49E-12	30	*CD34+CD38- fraction*<br>*euploid*<br>*FLT3-ITD*<br>*good prognosis*<br>*low centrosome aberrations*	*aneuploid*<br>*CD34+CD38+ fraction*<br>*high centrosome aberrations*<br>*poor prognosis*
M phase	3.09E-10	26	*CD34+CD38- fraction*<br>*euploid*<br>*good prognosis*	*aneuploid*<br>*CD34+CD38+ fraction*<br>*poor prognosis*
M phase of mitotic cell cycle	4.17E-09	22	*CD34+CD38- fraction*<br>*euploid*<br>*FLT3-ITD*<br>*good prognosis*	*aneuploid*<br>*CD34+CD38+ fraction*<br>*poor prognosis*
regulation of protein kinase activity	1.03E-08	22	*euploid*<br>*low centrosome aberrations*	*aneuploid*<br>*high centrosome aberrations*
regulation of kinase activity	1.53E-08	22	*euploid*<br>*low centrosome aberrations*	*aneuploid*<br>*high centrosome aberrations*
mitosis	1.89E-08	21	*CD34+CD38- fraction*<br>*euploid*<br>*FLT3-ITD*<br>*good prognosis*	*aneuploid*<br>*CD34+CD38+ fraction*<br>*poor prognosis*
regulation of transferase activity	2.26E-08	22	*euploid*<br>*low centrosome aberrations*	*aneuploid*<br>*high centrosome aberrations*
hemopoiesis	1.30E-06	16	*poor prognosis*	*good prognosis*<br>*inv(16)*
hemopoietic or lymphoid organ development	3.74E-06	16	*poor prognosis*	*good prognosis*<br>*inv(16)*
regulation of molecular function	1.11E-05	32	*euploid*<br>*low centrosome aberrations*	*aneuploid*<br>*good prognosis*<br>*high centrosome aberrations*
regulation of catalytic activity	1.51E-05	30	*euploid*<br>*low centrosome aberrations*	*aneuploid*<br>*good prognosis*<br>*high centrosome aberrations*
regulation of cyclin-dependent protein kinase activity	3.60E-05	9	*euploid*<br>*low centrosome aberrations*	*aneuploid*<br>*high centrosome aberrations*
interphase of mitotic cell cycle	3.71E-05	11	*CD34+CD38- fraction*<br>*euploid*<br>*good prognosis*<br>*low centrosome aberrations*	*aneuploid*<br>*CD34+CD38+ fraction*<br>*high centrosome aberrations*<br>*poor prognosis*
interphase	7.56E-05	11	*CD34+CD38- fraction*<br>*euploid*<br>*good prognosis*<br>*low centrosome aberrations*	*aneuploid*<br>*CD34+CD38+ fraction*<br>*high centrosome aberrations*<br>*poor prognosis*
cell division	1.00E-04	17	*CD34+CD38- fraction*<br>*euploid*<br>*good prognosis*<br>*low centrosome aberrations*	*aneuploid*<br>*CD34+CD38+ fraction*<br>*high centrosome aberrations*<br>*poor prognosis*
nervous system development	1.90E-04	30	*CD34+CD38+ fraction*<br>*inv(16)*<br>*poor prognosis*	*CD34+CD38- fraction*<br>*good prognosis*
leukocyte differentiation	5.20E-04	10		
leukocyte activation	1.92E-03	13	*FLT3 mutation*<br>*t(8;21)*	*inv(16)*

membrane organization and biogenesis	3.37E-03	16	*CD34+CD38- fraction*<br>*poor prognosis*<br>*t(8;21)*	*CD34+CD38+ fraction*<br>*inv(16)*<br>*MLL fusion gene*
catabolic process	5.10E-03	34		*MLL fusion gene*
barbed-end actin filament capping	8.47E-03	5		
actin filament capping	8.47E-03	5		
**Molecular Functions**				
identical protein binding	3.81E-05	21	*CD34+CD38+ fraction*<br>*FLT3 mutation*<br>*normal cytogenetics*<br>*t(8;21)*	*abnormal cytogenetics*<br>*CD34+CD38- fraction*<br>*MLL fusion gene*
GTPase activity	2.34E-03	16		
cytoskeletal protein binding	4.28E-03	24		*MLL fusion gene*
cytokine binding	6.78E-03	9		
**Cellular Components**				
soluble fraction	9.72E-06	17		
cytoplasmic part	4.28E-05	109	*FLT3-ITD*<br>*normal cytogenetics*<br>*t(8;21)*	*abnormal cytogenetics*<br>*FAB-M7*<br>*inv(16)*<br>*MLL fusion gene*
actin cytoskeleton	2.00E-04	18		
cytoskeleton	2.10E-04	46	*euploid*<br>*good prognosis*	*aneuploid*
cell surface	3.50E-04	12		
spindle	4.60E-04	9	*CD34+CD38- fraction*<br>*euploid*<br>*good prognosis*	*aneuploid*<br>*CD34+CD38+ fraction*<br>*poor prognosis*
coated pit	4.60E-04	7		

Significantly over-represented functional gene ontology (GO) categories of down-regulated genes associated with *NPM1* mutations are presented here. GO categories that are also over-represented in up-regulated genes associated with *NPM1* mutations are not included. Corrected p-value is the Bonferroni multiple hypothesis. Identification tags that are both up-regulated and down-regulated are not included in the 'other tags' columns. Identification tag descriptions can be found in Table S1.
