# Supplementary material for: Integrative Meta-Analysis of Differential Gene Expression in Acute Myeloid Leukemia
Source: PLoS One. 2010 Mar 1;5(3):e9466. doi: 10.1371/journal.pone.0009466 (PMC2830886; doi:10.1371/journal.pone.0009466)
Supplement: Table S15 — Functional categories of up-regulated genes associated with t(15;17) (0.02 MB PDF) [file pone.0009466.s015.pdf]

Table S15. Functional categories of up-regulated genes associated with t(15;17)

GO category	Corrected p-value	No. of genes	Other tags with upregulated genes	Other tags with downregulated genes
**Biological Processes**				
system development	1.62E-05	21	*CD34+CD38- fraction*	*CD34+CD38+ fraction*<br>*t(8;21)*
**Molecular Functions**				
calcium ion binding	2.04E-03	16	*inv(16)*	
**Cellular Components**				
cytoplasm	3.30E-03	44	*abnormal cytogenetics*<br>*aneuploid*<br>*FAB-M7*<br>*FLT3-ITD*<br>*FLT3 mutation*	*euploid*<br>*normal cytogenetics*
endoplasmic reticulum lumen	5.59E-03	4		
proteinaceous extracellular matrix	6.49E-03	8		
extracellular region	7.95E-03	20	*11q23*<br>*CD34+CD38+ fraction*<br>*inv(16)*<br>*NPM1 mutation*	*CD34+CD38- fraction*<br>*t(8;21)*
extracellular matrix	8.13E-03	8		

Significantly over-represented functional gene ontologt (GO) categories of up-regulated genes associated with t(15;17) are presented here. GO categories that are also over-represented in down-regulated genes associated with t(15;17) are not included. Corrected p-value is the Bonferroni multiple hypothesis. Identification tags that are both up-regulated and down-regulated are not included in the 'other tags' columns. Identification tag descriptions can be found in Table S1.
