# Supplementary material for: Integrative Meta-Analysis of Differential Gene Expression in Acute Myeloid Leukemia
Source: PLoS One. 2010 Mar 1;5(3):e9466. doi: 10.1371/journal.pone.0009466 (PMC2830886; doi:10.1371/journal.pone.0009466)
Supplement: Table S16 — Functional categories of down-regulated genes associated with t(15;17) (0.02 MB PDF) [file pone.0009466.s016.pdf]

Table S16. Functional categories of down-regulated genes associated with t(15;17)

GO category	Corrected p-value	No. of genes	Other tags with downregulated genes	Other tags with upregulated genes
**Biological Processes**				
biological regulation	1.40E-07	34	*CD34+CD38- fraction*<br>*CEBPA mutation*<br>*control*<br>*del(7q)*<br>*euploid*<br>*low centrosome aberrations*<br>*t(8;21)*	*aneuploid*<br>*CD34+CD38+ fraction*<br>*CEBPA silenced*<br>*high centrosome aberrations*<br>*normal cytogenetics*
regulation of biological process	2.33E-07	32	*CD34+CD38- fraction*<br>*CEBPA mutation*<br>*del(7q)*<br>*euploid*<br>*FLT3 mutation*<br>*low centrosome aberrations*<br>*t(8;21)*	*aneuploid*<br>*CD34+CD38+ fraction*<br>*CEBPA silenced*<br>*high centrosome aberrations*<br>*inv(16)*<br>*MLL fusion gene*
regulation of cellular process	3.21E-07	31	*CD34+CD38- fraction*<br>*CEBPA mutation*<br>*euploid*<br>*FLT3 mutation*<br>*low centrosome aberrations*<br>*t(8;21)*	*aneuploid*<br>*CD34+CD38+ fraction*<br>*CEBPA silenced*<br>*high centrosome aberrations*<br>*inv(16)*<br>*MLL fusion gene*
antigen processing and presentation of peptide or polysaccharide antigen via MHC class II	1.07E-05	6	*normal cytogenetics*	
anatomical structure morphogenesis	8.23E-05	11	*poor prognosis*<br>*t(8;21)*	*FLT3-ITD*<br>*FLT3 mutation*<br>*good prognosis*<br>*MLL fusion gene*<br>*normal cytogenetics*
regulation of metabolic process	3.50E-04	22	*CEBPA mutation*<br>*low centrosome aberrations*<br>*poor prognosis*	*good prognosis*<br>*high centrosome aberrations*<br>*NPM1 mutation*
regulation of cellular metabolic process	8.90E-04	21	*CEBPA mutation*<br>*low centrosome aberrations*<br>*poor prognosis*	*good prognosis*<br>*high centrosome aberrations*<br>*NPM1 mutation*
immune system process	1.02E-03	12	*CD34+CD38- fraction*<br>*FLT3 mutation*<br>*normal cytogenetics*<br>*t(8;21)*	*CD34+CD38+ fraction*<br>*FAB-M4*<br>*FAB-M5*<br>*inv(16)*<br>*MLL fusion gene*<br>*monocytic*
antigen processing and presentation	1.26E-03	7	*normal cytogenetics*	
regulation of gene expression	1.64E-03	20	*abnormal cytogenetics*<br>*CEBPA mutation*<br>*poor prognosis*	*good prognosis*<br>*NPM1 mutation*
response to stimulus	3.94E-03	18	*CD34+CD38- fraction*<br>*FLT3 mutation*<br>*t(8;21)*	*11q23*<br>*CD34+CD38+ fraction*<br>*inv(16)*<br>*MLL fusion gene*<br>*monocytic*
negative regulation of cellular process	5.51E-03	10	*CD34+CD38- fraction*<br>*t(8;21)*	*CD34+CD38+ fraction*<br>*control*<br>*inv(16)*<br>*MLL fusion gene*
ovulation	7.13E-03	2		
negative regulation of biological process	8.35E-03	10	*CD34+CD38- fraction*<br>*t(8;21)*	*CD34+CD38+ fraction*<br>*control*<br>*MLL fusion gene*<br>*inv(16)*<br>*MLL fusion gene*
**Molecular Functions**				

sequence-specific DNA binding	2.59E-07	13	*abnormal cytogenetics*<br>*control*	*FLT3-ITD*<br>*NPM1 mutation*
transcription factor activity	3.02E-06	15	*abnormal cytogenetics*<br>*control*<br>*MLL fusion gene*	*good prognosis*<br>*NPM1 mutation*
transcription regulator activity	4.86E-05	16	*abnormal cytogenetics*<br>*control*<br>*MLL fusion gene*<br>*poor prognosis*	*good prognosis*<br>*NPM1 mutation*
**Cellular Components**				
plasma membrane part	6.53E-08	20	*CEBPA mutation*<br>*good prognosis*	*11q23*<br>*CBF*<br>*CEBPA silenced*<br>*FAB-M4*<br>*FAB-M5*<br>*FAB-M7*<br>*inv(16)*<br>*poor prognosis*
plasma membrane	3.73E-06	23	*CD34+CD38- fraction*<br>*CEBPA mutation*	*11q23*<br>*CBF*<br>*CD34+CD38+ fraction*<br>*CEBPA silenced*<br>*control*<br>*FAB-M4*<br>*FAB-M5*<br>*FAB-M7*<br>*good prognosis*<br>*inv(16)*<br>*monocytic*
MHC class II protein complex	7.87E-06	6	*normal cytogenetics*	
intrinsic to plasma membrane	1.34E-03	11	*CD34+CD38- fraction*	*CBF*<br>*CD34+CD38+ fraction*<br>*FAB-M4*<br>*FAB-M5*<br>*FAB-M7*<br>*good prognosis*<br>*inv(16)*<br>*monocytic*
MHC protein complex	2.04E-03	6		
intracellular part	3.44E-03	39	*low centrosome aberrations*	*abnormal cytogenetics*<br>*FAB-M7*<br>*FLT3 mutation*<br>*high centrosome aberrations*
cell part	4.93E-03	53	*euploid*	*abnormal cytogenetics*<br>*aneuploid*<br>*FAB-M7*<br>*FLT3-ITD*<br>*FLT3 mutation*
cell	4.94E-03	53	*euploid*	*abnormal cytogenetics*<br>*aneuploid*<br>*FAB-M7*<br>*FLT3-ITD*<br>*FLT3 mutation*

Significantly over-represented functional gene ontology (GO) categories of down-regulated genes associated with t(15;17) are presented here. GO categories that are also over-represented in up-regulated genes associated with t(15;17) are not included. Corrected p-value is the Bonferroni multiple hypothesis. Identification tags that are both up-regulated and down-regulated are not included in the 'other tags' columns. Identification tag descriptions can be found in Table S1.
