# Supplementary material for: Integrative Meta-Analysis of Differential Gene Expression in Acute Myeloid Leukemia
Source: PLoS One. 2010 Mar 1;5(3):e9466. doi: 10.1371/journal.pone.0009466 (PMC2830886; doi:10.1371/journal.pone.0009466)
Supplement: Table S17 — Functional categories of up-regulated genes associated with inv(16) (0.03 MB PDF) [file pone.0009466.s017.pdf]

Table S17. Functional categories of up-regulated genes associated with inv(16)

GO category	Corrected p-value	No. of genes	Other tags with upregulated genes	Other tags with downregulated genes
**Biological Processes**				
response to external stimulus	4.34E-24	56	*CD34+CD38+ fraction<br>good prognosis<br>MLL fusion gene<br>monocytic<br>normal cytogenetics<br>NPM1 mutation*	*CD34+CD38- fraction<br>control<br>del(7q)<br>poor prognosis<br>t(8;21)*
response to wounding	5.77E-23	46	*CD34+CD38+ fraction<br>good prognosis<br>MLL fusion gene<br>monocytic<br>normal cytogenetics<br>NPM1 mutation*	*CD34+CD38- fraction<br>control<br>poor prognosis<br>t(8;21)*
response to stimulus	4.17E-19	123	*11q23<br>CD34+CD38+ fraction<br>MLL fusion gene<br>monocytic*	*CD34+CD38- fraction<br>FLT3 mutation<br>t(15;17)<br>t(8;21)*
immune system process	1.25E-16	69	*CD34+CD38+ fraction<br>FAB-M4<br>FAB-M5<br>MLL fusion gene<br>monocytic*	*CD34+CD38- fraction<br>FLT3 mutation<br>normal cytogenetics<br>t(15;17)<br>t(8;21)*
cell communication	2.65E-16	161	*CD34+CD38+ fraction<br>CEBPA silenced<br>good prognosis<br>normal cytogenetics*	*CD34+CD38- fraction<br>CEBPA mutation<br>poor prognosis<br>t(8;21)*
defense response	8.93E-16	44	*11q23<br>CD34+CD38+ fraction<br>good prognosis<br>MLL fusion gene<br>monocytic<br>normal cytogenetics*	*CD34+CD38- fraction<br>poor prognosis<br>t(8;21)*
inflammatory response	9.13E-16	32	*CD34+CD38+ fraction<br>MLL fusion gene<br>monocytic<br>NPM1 mutation*	*CD34+CD38- fraction<br>t(8;21)*
organ development	6.28E-15	60	*CD34+CD38- fraction*	*CD34+CD38+ fraction<br>normal cytogenetics<br>t(8;21)*
positive regulation of biological process	1.46E-14	58	*aneuploid<br>CD34+CD38+ fraction<br>good prognosis<br>high centrosome aberrations*	*CD34+CD38- fraction<br>CEBPA mutation<br>euploid<br>low centrosome aberrations<br>t(8;21)*
response to stress	3.86E-13	60	*abnormal cytogenetics<br>aneuploid<br>CD34+CD38+ fraction<br>high centrosome aberrations<br>MLL fusion gene<br>normal cytogenetics*	*CD34+CD38- fraction<br>euploid<br>low centrosome aberrations<br>t(8;21)*
signal transduction	4.23E-13	145	*CD34+CD38+ fraction<br>good prognosis<br>normal cytogenetics*	*CD34+CD38- fraction<br>CEBPA mutation<br>FLT3 mutation<br>poor prognosis<br>t(8;21)*
immune response	1.26E-12	57	*CD34+CD38+ fraction<br>FAB-M4<br>FAB-M5<br>monocytic*	*CD34+CD38- fraction<br>FLT3 mutation<br>normal cytogenetics<br>t(8;21)*

positive regulation of cellular process	2.30E-12	52	*aneuploid*<br>*CD34+CD38+ fraction*<br>*good prognosis*<br>*high centrosome aberrations*<br>*MLL fusion gene*<br>*normal cytogenetics*	*CD34+CD38- fraction*<br>*euploid*<br>*low centrosome aberrations*<br>*t(8;21)*
cell differentiation	7.39E-11	70	*CD34+CD38+ fraction*<br>*CEBPA silenced*<br>*t(11;19)*	*CD34+CD38- fraction*<br>*CEBPA mutation*
cellular developmental process	7.39E-11	70	*CD34+CD38+ fraction*<br>*CEBPA silenced*<br>*t(11;19)*	*CD34+CD38- fraction*<br>*CEBPA mutation*
biological adhesion	5.40E-10	47	*CD34+CD38- fraction*	*CD34+CD38+ fraction*<br>*normal cytogenetics*<br>*t(8;21)*
cell adhesion	5.40E-10	47	*CD34+CD38- fraction*	*CD34+CD38+ fraction*<br>*normal cytogenetics*<br>*t(8;21)*
negative regulation of cellular process	1.37E-08	48	*CD34+CD38+ fraction*<br>*control*<br>*MLL fusion gene*	*CD34+CD38- fraction*<br>*t(15;17)*<br>*t(8;21)*
negative regulation of biological process	2.24E-08	49	*CD34+CD38+ fraction*<br>*control*<br>*MLL fusion gene*	*CD34+CD38- fraction*<br>*t(15;17)*<br>*t(8;21)*
cell activation	3.87E-08	20	*good prognosis*<br>*NPM1 mutation*	*t(8;21)*
leukocyte activation	1.19E-07	18		*FLT3 mutation*<br>*NPM1 mutation*<br>*t(8;21)*
cell-cell signaling	2.63E-07	33		*t(8;21)*
cellular process	3.50E-07	352	*11q23*<br>*abnormal cytogenetics*<br>*aneuploid*<br>*CEBPA silenced*<br>*high centrosome aberrations*	*CEBPA mutation*<br>*del(7q)*<br>*euploid*<br>*low centrosome aberrations*<br>*t(8;21)*
regulation of biological process	4.22E-07	144	*aneuploid*<br>*CD34+CD38+ fraction*<br>*CEBPA silenced*<br>*high centrosome aberrations*<br>*MLL fusion gene*	*CD34+CD38- fraction*<br>*CEBPA mutation*<br>*del(7q)*<br>*euploid*<br>*FLT3 mutation*<br>*low centrosome aberrations*<br>*t(15;17)*<br>*t(8;21)*
endocytosis	4.82E-07	18	*CD34+CD38+ fraction*<br>*MLL fusion gene*	*CD34+CD38- fraction*<br>*CEBPA mutation*<br>*t(8;21)*
membrane invagination	4.82E-07	18	*CD34+CD38+ fraction*<br>*MLL fusion gene*	*CD34+CD38- fraction*<br>*CEBPA mutation*<br>*t(8;21)*
localization of cell	5.13E-07	25	*CD34+CD38+ fraction*<br>*good prognosis*<br>*MLL fusion gene*<br>*NPM1 mutation*	*CD34+CD38- fraction*<br>*normal cytogenetics*<br>*poor prognosis*<br>*t(8;21)*
cell motility	5.13E-07	25	*CD34+CD38+ fraction*<br>*good prognosis*<br>*MLL fusion gene*<br>*NPM1 mutation*	*CD34+CD38- fraction*<br>*normal cytogenetics*<br>*poor prognosis*<br>*t(8;21)*
membrane organization and biogenesis	9.16E-07	21	*CD34+CD38+ fraction*<br>*MLL fusion gene*	*CD34+CD38- fraction*<br>*NPM1 mutation*<br>*poor prognosis*<br>*t(8;21)*
immune effector process	1.59E-06	14	*NPM1 mutation*	*t(8;21)*
regulation of multicellular organismal process	1.82E-06	22	*FAB-M7*<br>*good prognosis*<br>*NPM1 mutation*	*t(8;21)*

regulation of cellular process	8.30E-06	134	*aneuploid*<br>*CD34+CD38+ fraction*<br>*CEBPA silenced*<br>*good prognosis*<br>*high centrosome aberrations*<br>*MLL fusion gene*	*CD34+CD38- fraction*<br>*CEBPA mutation*<br>*euploid*<br>*FLT3 mutation*<br>*low centrosome aberrations*<br>*t(15;17)*<br>*t(8;21)*
regulation of immune system process	1.39E-05	15	*good prognosis*<br>*NPM1 mutation*	*t(8;21)*
cell development	1.61E-05	45	*CD34+CD38+ fraction*<br>*normal cytogenetics*	*CD34+CD38- fraction*<br>*CEBPA mutation*<br>*t(8;21)*
regulation of developmental process	1.77E-05	35	*CD34+CD38+ fraction*<br>*CEBPA silenced*<br>*good prognosis*<br>*MLL fusion gene*	*CD34+CD38- fraction*<br>*CEBPA mutation*<br>*t(8;21)*
organ morphogenesis	6.75E-05	21	*good prognosis*<br>*normal cytogenetics*<br>*NPM1 mutation*	*poor prognosis*<br>*t(8;21)*
myeloid leukocyte activation	9.09E-05	7		
vesicle-mediated transport	9.70E-05	28	*MLL fusion gene*	*poor prognosis*<br>*t(8;21)*
humoral immune response	1.00E-04	10	*NPM1 mutation*	*t(8;21)*
response to chemical stimulus	1.70E-04	27	*CD34+CD38+ fraction*<br>*good prognosis*<br>*normal cytogenetics*<br>*NPM1 mutation*	*11q23*<br>*CD34+CD38- fraction*<br>*poor prognosis*<br>*t(8;21)*
positive regulation of developmental process	1.80E-04	20	*good prognosis*<br>*MLL fusion gene*	*poor prognosis*<br>*t(8;21)*
leukocyte mediated immunity	2.30E-04	10	*good prognosis*<br>*normal cytogenetics*<br>*NPM1 mutation*	*t(8;21)*
lymphocyte activation	3.10E-04	13		*FLT3 mutation*<br>*t(8;21)*
locomotory behavior	3.50E-04	14	*CD34+CD38+ fraction*<br>*NPM1 mutation*	*11q23*<br>*CD34+CD38- fraction*<br>*control*<br>*t(8;21)*
skeletal development	4.00E-04	15		*t(8;21)*
regulation of cell activation	5.10E-04	10	*good prognosis*<br>*NPM1 mutation*	*t(8;21)*
regulation of leukocyte activation	5.10E-04	10	*good prognosis*<br>*NPM1 mutation*	*t(8;21)*
positive regulation of metabolic process	6.10E-04	21	*good prognosis*<br>*high centrosome aberrations*	*low centrosome aberrations*<br>*poor prognosis*
transcription from RNA polymerase II promoter	6.30E-04	27	*abnormal cytogenetics*<br>*good prognosis*<br>*NPM1 mutation*	*del(7q)*<br>*MLL fusion gene*<br>*poor prognosis*<br>*t(8;21)*
blood vessel development	6.50E-04	13	*good prognosis*<br>*NPM1 mutation*	*MLL fusion gene*<br>*t(8;21)*
tissue development	6.70E-04	18		*t(8;21)*
cytokine production	7.90E-04	11	*CD34+CD38+ fraction*<br>*FAB-M4*<br>*FAB-M5*<br>*good prognosis*<br>*NPM1 mutation*	*CD34+CD38- fraction*<br>*t(8;21)*
vasculature development	8.00E-04	13	*good prognosis*<br>*NPM1 mutation*	*MLL fusion gene*<br>*t(8;21)*
wound healing	9.50E-04	12	*NPM1 mutation*	*t(8;21)*
taxis	1.10E-03	12	*CD34+CD38+ fraction*<br>*MLL fusion gene*<br>*NPM1 mutation*	*CD34+CD38- fraction*<br>*control*
chemotaxis	1.10E-03	12	*CD34+CD38+ fraction*<br>*MLL fusion gene*<br>*NPM1 mutation*	*CD34+CD38- fraction*<br>*control*

positive regulation of nucleobase, nucleoside, nucleotide and nucleic acid metabolic process	1.26E-03	17	*good prognosis*<br>*high centrosome aberrations*	*low centrosome aberrations*<br>*poor prognosis*
blood vessel morphogenesis	1.27E-03	12		*t(8;21)*
positive regulation of cellular metabolic process	1.51E-03	20	*good prognosis*<br>*high centrosome aberrations*	*low centrosome aberrations*
intracellular signaling cascade	1.72E-03	56	*good prognosis*<br>*MLL fusion gene*<br>*NPM1 mutation*	*poor prognosis*<br>*t(8;21)*
anatomical structure formation	1.94E-03	12		*t(8;21)*
immune system development	1.99E-03	13	*good prognosis*	*poor prognosis*
positive regulation of immune system process	2.27E-03	11	*good prognosis*<br>*NPM1 mutation*	*t(8;21)*
positive regulation of multicellular organismal process	2.38E-03	12	*NPM1 mutation*	*t(8;21)*
response to mechanical stimulus	2.67E-03	5		*t(8;21)*
hemopoiesis	3.10E-03	12	*good prognosis*	*NPM1 mutation*<br>*poor prognosis*
production of molecular mediator of immune response	3.12E-03	6	*NPM1 mutation*	*t(8;21)*
cell death	3.27E-03	32	*CD34+CD38+ fraction*<br>*MLL fusion gene*<br>*normal cytogenetics*	*CD34+CD38- fraction*<br>*CEBPA mutation*<br>*t(8;21)*
death	3.27E-03	32	*CD34+CD38+ fraction*<br>*MLL fusion gene*<br>*normal cytogenetics*	*CD34+CD38- fraction*<br>*CEBPA mutation*<br>*t(8;21)*
negative regulation of cell proliferation	5.14E-03	14	*aneuploid*	*euploid*<br>*t(8;21)*
behavior	5.55E-03	16	*CD34+CD38+ fraction*<br>*NPM1 mutation*	*CD34+CD38- fraction*
hemostasis	6.33E-03	10	*NPM1 mutation*	*t(8;21)*
localization	6.43E-03	103	*FAB-M7*<br>*good prognosis*<br>*MLL fusion gene*	*poor prognosis*<br>*t(8;21)*
hemopoietic or lymphoid organ development	6.53E-03	12	*good prognosis*	*NPM1 mutation*<br>*poor prognosis*
positive regulation of cell proliferation	6.58E-03	14	*MLL fusion gene*<br>*NPM1 mutation*	*poor prognosis*<br>*t(8;21)*
T cell activation	8.53E-03	9	*good prognosis*	
regulation of MAP kinase activity	9.28E-03	9		
regulation of mast cell cytokine production	9.75E-03	3	*good prognosis*<br>*normal cytogenetics*	*t(8;21)*
mast cell cytokine production	9.75E-03	3	*good prognosis*<br>*normal cytogenetics*	*t(8;21)*

Molecular Functions

signal transducer activity	2.14E-08	102	*CD34+CD38+ fraction*<br>*FAB-M4*<br>*FAB-M5*<br>*normal cytogenetics*	*CD34+CD38- fraction*<br>*t(8;21)*
molecular transducer activity	2.14E-08	102	*CD34+CD38+ fraction*<br>*FAB-M4*<br>*FAB-M5*<br>*normal cytogenetics*	*CD34+CD38- fraction*<br>*t(8;21)*
binding	2.00E-05	362	*aneuploid*<br>*CD34+CD38+ fraction*<br>*good prognosis*<br>*normal cytogenetics*	*CD34+CD38- fraction*<br>*euploid*<br>*t(8;21)*
receptor binding	2.20E-04	37	*11q23*<br>*NPM1 mutation*	*t(8;21)*
sugar binding	5.90E-04	16	*CD34+CD38+ fraction*	*CD34+CD38- fraction*
protein complex binding	7.70E-04	10		*t(8;21)*
transcription activator activity	1.04E-03	17	*good prognosis*<br>*NPM1 mutation*	*poor prognosis*<br>*t(8;21)*
calcium ion binding	1.87E-03	43	*t(15;17)*	
receptor activity	4.30E-03	73	*CD34+CD38+ fraction*<br>*FAB-M4*<br>*FAB-M5*	*CD34+CD38- fraction*<br>*t(8;21)*

transcription coactivator activity	6.17E-03	12	*good prognosis*	*poor prognosis*<br>*t(8;21)*

Cellular Components

plasma membrane	5.04E-26	144	*11q23*<br>*CBF*<br>*CD34+CD38+ fraction*<br>*CEBPA silenced*<br>*control*<br>*FAB-M4*<br>*FAB-M5*<br>*FAB-M7*<br>*good prognosis*<br>*monocytic*	*CD34+CD38- fraction*<br>*CEBPA mutation*<br>*t(15;17)*
integral to plasma membrane	1.87E-23	77	*CBF*<br>*CD34+CD38+ fraction*<br>*FAB-M4*<br>*FAB-M5*<br>*FAB-M7*<br>*good prognosis*<br>*monocytic*	*CD34+CD38- fraction*
intrinsic to plasma membrane	3.95E-23	77	*CBF*<br>*CD34+CD38+ fraction*<br>*FAB-M4*<br>*FAB-M5*<br>*FAB-M7*<br>*good prognosis*<br>*monocytic*	*CD34+CD38- fraction*<br>*t(15;17)*
membrane	2.89E-20	245	*FLT3 mutation*<br>*MLL fusion gene*	*normal cytogenetics*<br>*t(8;21)*
plasma membrane part	1.42E-19	96	*11q23*<br>*CBF*<br>*CEBPA silenced*<br>*FAB-M4*<br>*FAB-M5*<br>*FAB-M7*<br>*poor prognosis*	*CEBPA mutation*<br>*good prognosis*<br>*t(15;17)*
membrane part	2.50E-16	200	*CEBPA silenced*<br>*FAB-M4*<br>*FAB-M5*<br>*MLL fusion gene*	*CEBPA mutation*<br>*normal cytogenetics*<br>*t(8;21)*
intrinsic to membrane	5.79E-16	182	*CEBPA silenced*<br>*FAB-M4*<br>*FAB-M5*<br>*MLL fusion gene*	*t(8;21)*
integral to membrane	3.27E-15	178	*CEBPA silenced*<br>*FAB-M4*<br>*FAB-M5*<br>*MLL fusion gene*	*t(8;21)*
extracellular region	4.96E-09	77	*11q23*<br>*CD34+CD38+ fraction*<br>*NPM1 mutation*<br>*t(15;17)*	*CD34+CD38- fraction*<br>*t(8;21)*
membrane fraction	3.09E-06	31	*FAB-M7*<br>*MLL fusion gene*<br>*normal cytogenetics*	*del(7q)*<br>*t(8;21)*
Golgi apparatus	1.18E-05	31	*good prognosis*<br>*MLL fusion gene*	*poor prognosis*<br>*t(8;21)*
vacuole	4.52E-05	16	*CD34+CD38+ fraction*<br>*CEBPA mutation*<br>*FAB-M4*<br>*FAB-M5*<br>*good prognosis*<br>*MLL fusion gene*<br>*monocytic*	*CD34+CD38- fraction*<br>*CEBPA silenced*<br>*poor prognosis*<br>*t(8;21)*
melanosome	4.71E-05	10		*NRAS-PM*<br>*t(8;21)*
pigment granule	4.71E-05	10		*NRAS-PM*<br>*t(8;21)*

extracellular region part	9.37E-05	34	*11q23*<br>*CD34+CD38+ fraction*<br>*FLT3 mutation*<br>*NPM1 mutation*	*CD34+CD38- fraction*<br>*t(8;21)*
extracellular space	1.70E-04	24	*11q23*<br>*CD34+CD38+ fraction*<br>*FLT3 mutation*<br>*NPM1 mutation*	*CD34+CD38- fraction*<br>*t(8;21)*
lysosome	2.00E-04	14	*CD34+CD38+ fraction*<br>*CEBPA mutation*<br>*FAB-M4*<br>*FAB-M5*<br>*good prognosis*<br>*MLL fusion gene*<br>*monocytic*	*CD34+CD38- fraction*<br>*CEBPA silenced*<br>*poor prognosis*<br>*t(8;21)*
lytic vacuole	2.40E-04	14	*CD34+CD38+ fraction*<br>*CEBPA mutation*<br>*FAB-M4*<br>*FAB-M5*<br>*good prognosis*<br>*MLL fusion gene*<br>*monocytic*	*CD34+CD38- fraction*<br>*CEBPA silenced*<br>*poor prognosis*<br>*t(8;21)*
cytoplasmic part	3.20E-04	105	*abnormal cytogenetics*<br>*FAB-M7*<br>*MLL fusion gene*	*FLT3-ITD*<br>*normal cytogenetics*<br>*NPM1 mutation*<br>*t(8;21)*
Golgi membrane	4.50E-04	19		*poor prognosis*<br>*t(8;21)*
cytoplasmic vesicle	9.70E-04	20	*MLL fusion gene*	*t(8;21)*
Golgi apparatus part	9.70E-04	20	*MLL fusion gene*	*poor prognosis*<br>*t(8;21)*
vesicle	1.26E-03	20	*MLL fusion gene*	*t(8;21)*
endomembrane system	1.96E-03	34	*CEBPA silenced*<br>*good prognosis*<br>*MLL fusion gene*<br>*NPM1 mutation*	*CEBPA mutation*<br>*poor prognosis*<br>*t(8;21)*
integrin complex	1.98E-03	7		*t(8;21)*
receptor complex	4.54E-03	9	*MLL fusion gene*	*t(8;21)*
lipid raft	7.20E-03	6		*t(8;21)*
cytoplasmic membrane-bound vesicle	8.20E-03	16	*MLL fusion gene*	*t(8;21)*
membrane-bound vesicle	9.69E-03	16	*MLL fusion gene*	*t(8;21)*

Significantly over-represented functional gene ontology (GO) categories of up-regulated genes associated with inv(16) are presented here. GO categories that are also over-represented in down-regulated genes associated with inv(16) are not included. Corrected p-value is the Bonferroni multiple hypothesis. Identification tags that are both up-regulated and down-regulated are not included in the 'other tags' columns. Identification tag descriptions can be found in Table S1.
