# Supplementary material for: Integrative Meta-Analysis of Differential Gene Expression in Acute Myeloid Leukemia
Source: PLoS One. 2010 Mar 1;5(3):e9466. doi: 10.1371/journal.pone.0009466 (PMC2830886; doi:10.1371/journal.pone.0009466)
Supplement: Table S18 — Functional categories of down-regulated genes associated with inv(16) (0.02 MB PDF) [file pone.0009466.s018.pdf]

Table S18. Functional categories of down-regulated genes associated with inv(16)

GO category	Corrected p-value	No. of genes	Other tags with downregulated genes	Other tags with upregulated genes
**Biological Processes**				
nervous system development	4.70E-04	19	*CD34+CD38+ fraction*<br>*NPM1 mutation*<br>*poor prognosis*	*CD34+CD38- fraction*<br>*good prognosis*
**Molecular Functions - none**				
**Cellular Components - none**				

Significantly over-represented functional gene ontology (GO) categories of down-regulated genes associated with inv(16) are presented here. GO categories that are also over-represented in up-regulated genes associated with inv(16) are not included. Corrected p-value is the Bonferroni multiple hypothesis. Identification tags that are both up-regulated and down-regulated are not included in the 'other tags' columns. Identification tag descriptions can be found in Table S1.
