# Supplementary material for: Integrative Meta-Analysis of Differential Gene Expression in Acute Myeloid Leukemia
Source: PLoS One. 2010 Mar 1;5(3):e9466. doi: 10.1371/journal.pone.0009466 (PMC2830886; doi:10.1371/journal.pone.0009466)
Supplement: Table S19 — Functional categories of up-regulated genes associated with t(8;21) (0.01 MB PDF) [file pone.0009466.s019.pdf]

Table S19. Functional categories of up-regulated genes associated with t(8;21)

GO category	Corrected p-value	No. of genes	Other tags with upregulated genes	Other tags with downregulated genes
**Biological Processes - none**				
**Molecular Functions - none**				
**Cellular Components**				
centrosome	6.43E-03	7	*aneuploid*	*euploid*

Significantly over-represented functional gene ontology (GO) categories of up-regulated genes associated with t(8;21) are presented here. GO categories that are also over-represented in down-regulated genes associated with t(8;21) are not included. Corrected p-value is the Bonferroni multiple hypothesis. Identification tags that are both up-regulated and down-regulated are not included in the 'other tags' coolumns. Identification tag descriptions can be found in Table S1.
