# Supplementary material for: Integrative Meta-Analysis of Differential Gene Expression in Acute Myeloid Leukemia
Source: PLoS One. 2010 Mar 1;5(3):e9466. doi: 10.1371/journal.pone.0009466 (PMC2830886; doi:10.1371/journal.pone.0009466)
Supplement: Table S20 — Functional categories of down-regulated genes associated with t(8;21) (0.03 MB PDF) [file pone.0009466.s020.pdf]

Table S20. Functional categories of down-regulated genes associated with t(8;21)

GO category	Corrected p-value	No. of genes	Other tags with downregulated genes	Other tags with upregulated genes
**Biological Processes**				
developmental process	1.56E-27	133	*CEBPA mutation*<br>*control*	*11q23*<br>*CEBPA silenced*<br>*FAB-M7*<br>*FLT3-ITD*<br>*FLT3 mutation*<br>*t(11;19)*
multicellular organismal process	3.36E-24	137	*CEBPA mutation*<br>*control*<br>*del(7q)*	*11q23*<br>*FAB-M4*<br>*FAB-M5*<br>*FAB-M7*<br>*FLT3-ITD*<br>*FLT3 mutation*<br>*good prognosis*
response to external stimulus	3.48E-22	53	*CD34+CD38- fraction*<br>*control*<br>*del(7q)*<br>*poor prognosis*	*CD34+CD38+ fraction*<br>*good prognosis*<br>*inv(16)*<br>*MLL fusion gene*<br>*monocytic*<br>*normal cytogenetics*<br>*NPM1 mutation*
multicellular organismal development	2.77E-21	100	*CD34+CD38+ fraction*<br>*CEBPA mutation*<br>*control*	*11q23*<br>*CD34+CD38- fraction*<br>*FAB-M7*<br>*FLT3-ITD*
response to wounding	1.06E-20	43	*CD34+CD38- fraction*<br>*control*<br>*poor prognosis*	*CD34+CD38+ fraction*<br>*good prognosis*<br>*inv(16)*<br>*MLL fusion gene*<br>*monocytic*<br>*normal cytogenetics*<br>*NPM1 mutation*
system development	3.83E-17	75	*CD34+CD38+ fraction*	*CD34+CD38- fraction*<br>*t(15;17)*
cell communication	4.07E-16	157	*CD34+CD38- fraction*<br>*CEBPA mutation*<br>*poor prognosis*	*CD34+CD38+ fraction*<br>*CEBPA silenced*<br>*good prognosis*<br>*inv(16)*<br>*normal cytogenetics*
response to stimulus	1.36E-14	111	*CD34+CD38- fraction*<br>*FLT3 mutation*<br>*t(15;17)*	*11q23*<br>*CD34+CD38+ fraction*<br>*inv(16)*<br>*MLL fusion gene*<br>*monocytic*
signal transduction	2.33E-14	145	*CD34+CD38- fraction*<br>*CEBPA mutation*<br>*FLT3 mutation*<br>*poor prognosis*	*CD34+CD38+ fraction*<br>*good prognosis*<br>*inv(16)*<br>*normal cytogenetics*
organ development	2.60E-14	58	*CD34+CD38+ fraction*<br>*normal cytogenetics*	*CD34+CD38- fraction*<br>*inv(16)*
biological regulation	1.18E-13	172	*CD34+CD38- fraction*<br>*CEBPA mutation*<br>*control*<br>*del(7q)*<br>*euploid*<br>*low centrosome aberrations*<br>*t(15;17)*	*aneuploid*<br>*CD34+CD38+ fraction*<br>*CEBPA silenced*<br>*high centrosome aberrations*<br>*normal cytogenetics*
cell proliferation	1.94E-13	45	*CD34+CD38- fraction*<br>*euploid*<br>*normal cytogenetics*	*aneuploid*<br>*CD34+CD38+ fraction*<br>*FLT3-ITD*<br>*FLT3 mutation*

positive regulation of biological process	2.69E-13	55	*CD34+CD38- fraction*<br>*CEBPA mutation*<br>*euploid*<br>*low centrosome aberrations*	*aneuploid*<br>*CD34+CD38+ fraction*<br>*good prognosis*<br>*high centrosome aberrations*<br>*inv(16)*
defense response	1.75E-12	39	*CD34+CD38- fraction*<br>*poor prognosis*	*11q23*<br>*CD34+CD38+ fraction*<br>*good prognosis*<br>*inv(16)*<br>*MLL fusion gene*<br>*monocytic*<br>*normal cytogenetics*
inflammatory response	2.00E-12	28	*CD34+CD38- fraction*	*CD34+CD38+ fraction*<br>*inv(16)*<br>*MLL fusion gene*<br>*monocytic*<br>*NPM1 mutation*
response to stress	5.22E-12	57	*CD34+CD38- fraction*<br>*euploid*<br>*low centrosome aberrations*	*abnormal cytogenetics*<br>*aneuploid*<br>*CD34+CD38+ fraction*<br>*high centrosome aberrations*<br>*inv(16)*<br>*MLL fusion gene*<br>*normal cytogenetics*
positive regulation of cellular process	4.44E-11	49	*CD34+CD38- fraction*<br>*euploid*<br>*low centrosome aberrations*	*aneuploid*<br>*CD34+CD38+ fraction*<br>*good prognosis*<br>*high centrosome aberrations*<br>*inv(16)*<br>*MLL fusion gene*<br>*normal cytogenetics*
immune system process	6.18E-11	58	*CD34+CD38- fraction*<br>*FLT3 mutation*<br>*normal cytogenetics*<br>*t(15;17)*	*CD34+CD38+ fraction*<br>*FAB-M4*<br>*FAB-M5*<br>*inv(16)*<br>*MLL fusion gene*<br>*monocytic*
regulation of biological process	1.05E-09	149	*CD34+CD38- fraction*<br>*CEBPA mutation*<br>*del(7q)*<br>*euploid*<br>*FLT3 mutation*<br>*low centrosome aberrations*<br>*t(15;17)*	*aneuploid*<br>*CD34+CD38+ fraction*<br>*CEBPA silenced*<br>*high centrosome aberrations*<br>*inv(16)*<br>*MLL fusion gene*
cellular process	1.14E-09	351	*CEBPA mutation*<br>*del(7q)*<br>*euploid*<br>*low centrosome aberrations*	*11q23*<br>*abnormal cytogenetics*<br>*aneuploid*<br>*CEBPA silenced*<br>*high centrosome aberrations*<br>*inv(16)*
negative regulation of cellular process	1.30E-09	49	*CD34+CD38- fraction*<br>*t(15;17)*	*CD34+CD38+ fraction*<br>*control*<br>*inv(16)*<br>*MLL fusion gene*
negative regulation of biological process	2.17E-09	50	*CD34+CD38- fraction*<br>*t(15;17)*	*CD34+CD38+ fraction*<br>*control*<br>*inv(16)*<br>*MLL fusion gene*
biological adhesion	2.61E-09	45	*CD34+CD38+ fraction*<br>*normal cytogenetics*	*CD34+CD38- fraction*<br>*inv(16)*
cell adhesion	2.61E-09	45	*CD34+CD38+ fraction*<br>*normal cytogenetics*	*CD34+CD38- fraction*<br>*inv(16)*
regulation of cell proliferation	2.56E-08	29	*CD34+CD38+ fraction*<br>*euploid*<br>*poor prognosis*	*aneuploid*<br>*CD34+CD38- fraction*<br>*good prognosis*<br>*MLL fusion gene*<br>*NPM1 mutation*

regulation of multicellular organismal process	3.05E-08	24		*FAB-M7*<br>*good prognosis*<br>*inv(16)*<br>*NPM1 mutation*
immune response	3.57E-08	48	*CD34+CD38- fraction*<br>*FLT3 mutation*<br>*normal cytogenetics*	*CD34+CD38+ fraction*<br>*FAB-M4*<br>*FAB-M5*<br>*inv(16)*<br>*monocytic*
localization of cell	5.12E-08	26	*CD34+CD38- fraction*<br>*normal cytogenetics*<br>*poor prognosis*	*CD34+CD38+ fraction*<br>*good prognosis*<br>*inv(16)*<br>*MLL fusion gene*<br>*NPM1 mutation*
cell motility	5.12E-08	26	*CD34+CD38- fraction*<br>*normal cytogenetics*<br>*poor prognosis*	*CD34+CD38+ fraction*<br>*good prognosis*<br>*inv(16)*<br>*MLL fusion gene*<br>*NPM1 mutation*
immune effector process	1.03E-07	15		*inv(16)*<br>*NPM1 mutation*
regulation of cellular process	1.33E-07	137	*CD34+CD38- fraction*<br>*CEBPA mutation*<br>*euploid*<br>*FLT3 mutation*<br>*low centrosome aberrations*<br>*t(15;17)*	*aneuploid*<br>*CD34+CD38+ fraction*<br>*CEBPA silenced*<br>*good prognosis*<br>*high centrosome aberrations*<br>*inv(16)*<br>*MLL fusion gene*
anatomical structure morphogenesis	2.12E-07	39	*poor prognosis*<br>*t(15;17)*	*FLT3-ITD*<br>*FLT3 mutation*<br>*good prognosis*<br>*MLL fusion gene*<br>*normal cytogenetics*
regulation of immune system process	1.17E-06	16		*good prognosis*<br>*inv(16)*<br>*NPM1 mutation*
regulation of developmental process	2.42E-06	36	*CD34+CD38- fraction*<br>*CEBPA mutation*	*CD34+CD38+ fraction*<br>*CEBPA silenced*<br>*good prognosis*<br>*inv(16)*<br>*MLL fusion gene*
cell development	6.39E-06	45	*CD34+CD38- fraction*<br>*CEBPA mutation*	*CD34+CD38+ fraction*<br>*inv(16)*<br>*normal cytogenetics*
cell activation	8.32E-06	17		*good prognosis*<br>*inv(16)*<br>*NPM1 mutation*
endocytosis	1.54E-05	16	*CD34+CD38- fraction*<br>*CEBPA mutation*	*CD34+CD38+ fraction*<br>*inv(16)*<br>*MLL fusion gene*
membrane invagination	1.54E-05	16	*CD34+CD38- fraction*<br>*CEBPA mutation*	*CD34+CD38+ fraction*<br>*inv(16)*<br>*MLL fusion gene*
leukocyte mediated immunity	1.60E-05	11		*good prognosis*<br>*inv(16)*<br>*normal cytogenetics*<br>*NPM1 mutation*
positive regulation of developmental process	2.38E-05	21	*poor prognosis*	*good prognosis*<br>*inv(16)*<br>*MLL fusion gene*
cell-cell signaling	2.77E-05	29		*inv(16)*
positive regulation of immune system process	2.79E-05	13		*good prognosis*<br>*inv(16)*<br>*NPM1 mutation*
leukocyte activation	3.26E-05	15	*FLT3 mutation*<br>*NPM1 mutation*	*inv(16)*
positive regulation of multicellular organismal process	3.65E-05	14		*inv(16)*<br>*NPM1 mutation*

organ morphogenesis	4.03E-05	21	*poor prognosis*	*good prognosis*<br>*inv(16)*<br>*normal cytogenetics*<br>*NPM1 mutation*
humoral immune response	7.58E-05	10		*inv(16)*<br>*NPM1 mutation*
membrane organization and biogenesis	9.41E-05	18	*CD34+CD38- fraction*<br>*NPM1 mutation*<br>*poor prognosis*	*CD34+CD38+ fraction*<br>*inv(16)*<br>*MLL fusion gene*
wound healing	9.52E-05	13		*inv(16)*<br>*NPM1 mutation*
transcription from RNA polymerase II promoter	1.00E-04	28	*del(7q)*<br>*MLL fusion gene*<br>*poor prognosis*	*abnormal cytogenetics*<br>*good prognosis*<br>*inv(16)*<br>*NPM1 mutation*
regulation of biological quality	1.90E-04	39	*poor prognosis*	*FAB-M7*<br>*good prognosis*<br>*MLL fusion gene*<br>*t(8;14)*
activation of immune response	4.00E-04	9		*NPM1 mutation*
blood vessel development	4.60E-04	13	*MLL fusion gene*	*good prognosis*<br>*inv(16)*<br>*NPM1 mutation*
vasculature development	5.70E-04	13	*MLL fusion gene*	*good prognosis*<br>*inv(16)*<br>*NPM1 mutation*
cell death	6.10E-04	33	*CD34+CD38- fraction*<br>*CEBPA mutation*	*CD34+CD38+ fraction*<br>*inv(16)*<br>*MLL fusion gene*<br>*normal cytogenetics*
death	6.10E-04	33	*CD34+CD38- fraction*<br>*CEBPA mutation*	*CD34+CD38+ fraction*<br>*inv(16)*<br>*MLL fusion gene*<br>*normal cytogenetics*
vesicle-mediated transport	6.20E-04	26	*poor prognosis*	*inv(16)*<br>*MLL fusion gene*
hemostasis	6.40E-04	11		*inv(16)*<br>*NPM1 mutation*
immunoglobulin mediated immune response	7.50E-04	8		
lymphocyte mediated immunity	8.30E-04	9		*NPM1 mutation*
B cell mediated immunity	8.70E-04	8		
blood vessel morphogenesis	9.30E-04	12		*inv(16)*
regulation of immune response	1.16E-03	10		*NPM1 mutation*
adaptive immune response based on somatic recombination of immune receptors built from immunoglobulin superfamily domains	1.30E-03	9		*NPM1 mutation*
adaptive immune response	1.30E-03	9		*NPM1 mutation*
apoptosis	1.37E-03	31	*CD34+CD38- fraction*<br>*CEBPA mutation*	*CD34+CD38+ fraction*<br>*MLL fusion gene*<br>*normal cytogenetics*
anatomical structure formation	1.42E-03	12		*inv(16)*
intracellular signaling cascade	1.44E-03	55	*poor prognosis*	*good prognosis*<br>*inv(16)*<br>*MLL fusion gene*<br>*NPM1 mutation*
skeletal development	1.55E-03	14		*inv(16)*
programmed cell death	1.66E-03	31	*CD34+CD38- fraction*	*CD34+CD38+ fraction*<br>*MLL fusion gene*<br>*normal cytogenetics*
regulation of apoptosis	1.80E-03	24	*CD34+CD38- fraction*<br>*CEBPA mutation*	*CD34+CD38+ fraction*<br>*CEBPA silenced*<br>*good prognosis*<br>*MLL fusion gene*
tissue development	1.96E-03	17		*inv(16)*

regulation of programmed cell death	2.21E-03	24	*CEBPA mutation*	*CEBPA silenced*<br>*good prognosis*<br>*MLL fusion gene*
response to mechanical stimulus	2.30E-03	5		*inv(16)*
positive regulation of immune response	2.43E-03	9		*NPM1 mutation*
production of molecular mediator of immune response	2.62E-03	6		*inv(16)*<br>*NPM1 mutation*
regulation of body fluid levels	2.65E-03	11		*NPM1 mutation*
blood coagulation	3.27E-03	10		*NPM1 mutation*
rhythmic process	3.50E-03	8		
regulation of cell activation	3.57E-03	9		*good prognosis*<br>*inv(16)*<br>*NPM1 mutation*
regulation of leukocyte activation	3.57E-03	9		*good prognosis*<br>*inv(16)*<br>*NPM1 mutation*
negative regulation of cell proliferation	3.63E-03	14	*euploid*	*aneuploid*<br>*inv(16)*
response to chemical stimulus	3.72E-03	24	*11q23*<br>*CD34+CD38- fraction*<br>*poor prognosis*	*CD34+CD38+ fraction*<br>*good prognosis*<br>*inv(16)*<br>*normal cytogenetics*<br>*NPM1 mutation*
cytokine production	4.49E-03	10	*CD34+CD38- fraction*	*CD34+CD38+ fraction*<br>*FAB-M4*<br>*FAB-M5*<br>*good prognosis*<br>*inv(16)*<br>*NPM1 mutation*
coagulation	4.49E-03	10		
positive regulation of cell proliferation	4.66E-03	14	*poor prognosis*	*inv(16)*<br>*MLL fusion gene*<br>*NPM1 mutation*
regulation of transcription from RNA polymerase II promoter	6.90E-03	19	*CEBPA mutation*<br>*MLL fusion gene*	*good prognosis*<br>*normal cytogenetics*<br>*NPM1 mutation*
localization	8.23E-03	100	*poor prognosis*	*FAB-M7*<br>*good prognosis*<br>*inv(16)*<br>*MLL fusion gene*
regulation of response to stimulus	8.32E-03	11		*good prognosis*<br>*NPM1 mutation*
locomotory behavior	8.56E-03	12	*11q23*<br>*CD34+CD38- fraction*<br>*control*	*CD34+CD38+ fraction*<br>*inv(16)*<br>*NPM1 mutation*
regulation of mast cell cytokine production	8.92E-03	3		*good prognosis*<br>*inv(16)*<br>*normal cytogenetics*
mast cell cytokine production	8.92E-03	3		*good prognosis*<br>*inv(16)*<br>*normal cytogenetics*
lymphocyte activation	9.42E-03	11	*FLT3 mutation*	*inv(16)*
negative regulation of cytokine production	9.49E-03	4		

Molecular Functions

signal transducer activity	3.82E-08	99	*CD34+CD38- fraction*	*CD34+CD38+ fraction*<br>*FAB-M4*<br>*FAB-M5*<br>*inv(16)*<br>*normal cytogenetics*
molecular transducer activity	3.82E-08	99	*CD34+CD38- fraction*	*CD34+CD38+ fraction*<br>*FAB-M4*<br>*FAB-M5*<br>*inv(16)*<br>*normal cytogenetics*

binding	1.06E-05	353	*CD34+CD38- fraction*<br>*euploid*	*aneuploid*<br>*CD34+CD38+ fraction*<br>*good prognosis*<br>*inv(16)*<br>*normal cytogenetics*
enzyme regulator activity	6.45E-05	44		
protein complex binding	5.80E-04	10		*inv(16)*
transcription activator activity	6.80E-04	17	*poor prognosis*	*good prognosis*<br>*inv(16)*<br>*NPM1 mutation*
receptor binding	7.70E-04	35		*11q23*<br>*inv(16)*<br>*NPM1 mutation*
transcription coactivator activity	4.53E-03	12	*poor prognosis*	*good prognosis*<br>*inv(16)*
enzyme activator activity	4.92E-03	20		
identical protein binding	6.43E-03	17	*CD34+CD38+ fraction*<br>*FLT3 mutation*<br>*normal cytogenetics*<br>*NPM1 mutation*	*abnormal cytogenetics*<br>*CD34+CD38- fraction*<br>*MLL fusion gene*
IgE binding	8.42E-03	3		
receptor activity	9.20E-03	70	*CD34+CD38- fraction*	*CD34+CD38+ fraction*<br>*FAB-M4*<br>*FAB-M5*<br>*inv(16)*
structural constituent of cytoskeleton	9.41E-03	8		
enzyme inhibitor activity	9.87E-03	18		
**Cellular Components**				
membrane	7.53E-18	233	*normal cytogenetics*	*FLT3 mutation*<br>*inv(16)*<br>*MLL fusion gene*
membrane part	5.49E-13	186	*CEBPA mutation*<br>*normal cytogenetics*	*CEBPA silenced*<br>*FAB-M4*<br>*FAB-M5*<br>*inv(16)*<br>*MLL fusion gene*
intrinsic to membrane	4.60E-12	167		*CEBPA silenced*<br>*FAB-M4*<br>*FAB-M5*<br>*inv(16)*<br>*MLL fusion gene*
integral to membrane	4.98E-11	162		*CEBPA silenced*<br>*FAB-M4*<br>*FAB-M5*<br>*inv(16)*<br>*MLL fusion gene*
extracellular region	2.96E-09	76	*CD34+CD38- fraction*	*11q23*<br>*CD34+CD38+ fraction*<br>*inv(16)*<br>*NPM1 mutation*<br>*t(15;17)*
intracellular organelle	1.77E-07	216	*CD34+CD38+ fraction*<br>*low centrosome aberrations*	*abnormal cytogenetics*<br>*CD34+CD38- fraction*<br>*FLT3-TKD*<br>*high centrosome aberrations*
organelle	1.83E-07	216	*CD34+CD38+ fraction*<br>*low centrosome aberrations*	*abnormal cytogenetics*<br>*CD34+CD38- fraction*<br>*FLT3-TKD*<br>*high centrosome aberrations*
cytoplasmic part	4.25E-07	113	*FLT3-ITD*<br>*normal cytogenetics*<br>*NPM1 mutation*	*abnormal cytogenetics*<br>*FAB-M7*<br>*inv(16)*<br>*MLL fusion gene*
Golgi apparatus	1.80E-06	32	*poor prognosis*	*good prognosis*<br>*inv(16)*<br>*MLL fusion gene*

membrane fraction	2.00E-05	29	*del(7q)*	*FAB-M7*<br>*inv(16)*<br>*MLL fusion gene*<br>*normal cytogenetics*
melanosome	3.77E-05	10	*NRAS-PM*	*inv(16)*
pigment granule	3.77E-05	10	*NRAS-PM*	*inv(16)*
cytoplasmic vesicle	4.43E-05	22		*inv(16)*<br>*MLL fusion gene*
vesicle	6.02E-05	22		*inv(16)*<br>*MLL fusion gene*
endomembrane system	1.50E-04	36	*CEBPA mutation*<br>*poor prognosis*	*CEBPA silenced*<br>*good prognosis*<br>*inv(16)*<br>*MLL fusion gene*<br>*NPM1 mutation*
vacuole	1.70E-04	15	*CD34+CD38- fraction*<br>*CEBPA silenced*<br>*poor prognosis*	*CD34+CD38+ fraction*<br>*CEBPA mutation*<br>*FAB-M4*<br>*FAB-M5*<br>*good prognosis*<br>*inv(16)*<br>*MLL fusion gene*<br>*monocytic*
Golgi membrane	3.00E-04	19	*poor prognosis*	*inv(16)*
Golgi apparatus part	6.50E-04	20	*poor prognosis*	*inv(16)*<br>*MLL fusion gene*
lysosome	8.50E-04	13	*CD34+CD38- fraction*<br>*CEBPA silenced*<br>*poor prognosis*	*CD34+CD38+ fraction*<br>*CEBPA mutation*<br>*FAB-M4*<br>*FAB-M5*<br>*good prognosis*<br>*inv(16)*<br>*MLL fusion gene*<br>*monocytic*
lytic vacuole	9.90E-04	13	*CD34+CD38- fraction*<br>*CEBPA silenced*<br>*poor prognosis*	*CD34+CD38+ fraction*<br>*CEBPA mutation*<br>*FAB-M4*<br>*FAB-M5*<br>*good prognosis*<br>*inv(16)*<br>*MLL fusion gene*<br>*monocytic*
extracellular region part	1.07E-03	31	*CD34+CD38- fraction*	*11q23*<br>*CD34+CD38+ fraction*<br>*FLT3 mutation*<br>*inv(16)*<br>*NPM1 mutation*
extracellular space	1.26E-03	22	*CD34+CD38- fraction*	*11q23*<br>*CD34+CD38+ fraction*<br>*FLT3 mutation*<br>*inv(16)*<br>*NPM1 mutation*
cytoplasmic membrane-bound vesicle	1.60E-03	17		*inv(16)*<br>*MLL fusion gene*
integrin complex	1.72E-03	7		*inv(16)*
membrane-bound vesicle	1.92E-03	17		*inv(16)*<br>*MLL fusion gene*
endoplasmic reticulum	2.19E-03	30	*poor prognosis*	*CEBPA silenced*<br>*MLL fusion gene*
organelle membrane	3.03E-03	43	*poor prognosis*	*MLL fusion gene*
receptor complex	3.78E-03	9		*inv(16)*<br>*MLL fusion gene*
lipid raft	6.43E-03	6		*inv(16)*

Significantly over-represented functional gene ontology (GO) categories of down-regulated genes associated with t(8;21) are presented here. GO categories that are also over-represented in up-regulated genes associated with t(8;21) are not included. Corrected p-value is the Bonferroni multiple hypothesis. Identification tags that are both up-regulated and down-regulated are not included in the 'other tags' columns. Identification tag descriptions can be found in Table S1.
